# Collective Asymmetric Total Syntheses of Musellarins A–E

**DOI:** 10.1021/acs.joc.5c02575

**Published:** 2025-11-28

**Authors:** Yichen Liu, Yanghui Ou, Vincent Chang, Hongliang Yao, Rongbiao Tong

**Affiliations:** † Department of Chemistry, 58207 The Hong Kong University of Science and Technology, Clearwater Bay, Kowloon, Hong Kong, China; ‡ Guangdong Key Laboratory of Animal Conservation and Resource Utilization, Institute of Zoology, 514144 Guangdong Academy of Sciences, Guangzhou, Guangdong 510260, China

## Abstract

Musellarins represent a rare class of diarylheptanoid natural products with cytotoxic activity. While total synthesis of musellarins A–C has been reported, musellarins D and E with *trans*-fused-2,6-*cis*-dihydropyran (DHP) remain synthetically unexplored. An Achmatowicz rearrangement-based synthetic strategy is developed for the collective total synthesis of musellarins A–E, where synthetic musellarins D and E are achieved for the first time and allow us to correct the misassigned configuration of carbon-10b (C_10b_) as a *cis*-fused-2,6-*cis*-THP motif. Notably, palladium-catalyzed arylation of Achmatowicz rearrangement products with boronic acids enables access to both 2,6-*trans*- and 2,6-*cis*-dihydropyrans (DHPs), corresponding to musellarins A–C and musellarins D–E, respectively.

## Graphical abstract

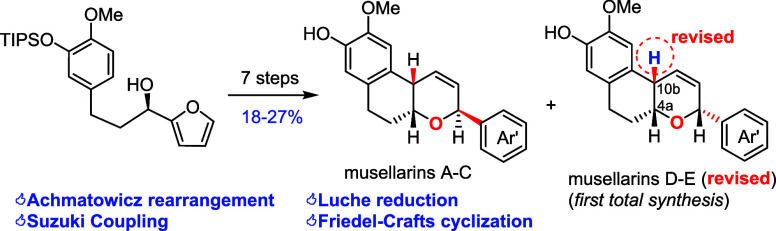

## Introduction

Musellarins A–E are a diarylheptanoid family with a rare bicyclic dihydropyran (DHP) skeleton (Figure 1). Musellarin A was first reported in 2002 by Kinghorn and co-workers from *Musa paradisiaca* in Peru^1^ and subsequently in 2011 isolated again along with its congeners musellarins B–E by Zhao and co-workers from *Musella lasiocarpa* in Yunan, China.^2^ Musellarin A was found to induce the quinone reductase activity, and musellarin B showed moderate cytotoxicity (>18 μM) against several cancer cell lines. Further biological studies were not reported, probably due to the insufficient amounts obtained from natural sources. Structurally, musellarins A–C differ from musellarins D–E by two stereocenters at C_3_ and C_10b_ of DHP ring, which resulted in *cis*-*trans* configuration [(4a,10b)-*cis*, (4a,3)-*trans*] for musellarins A–C and *trans–cis* configuration [(4a,10b)-*trans*, (4a,3)*-cis*] for musellarins D–E. Such stereochemical difference was not rationalized by their possible biosynthetic pathways and might give rise to synthetic challenges.^3,4^

**Figure 1:**
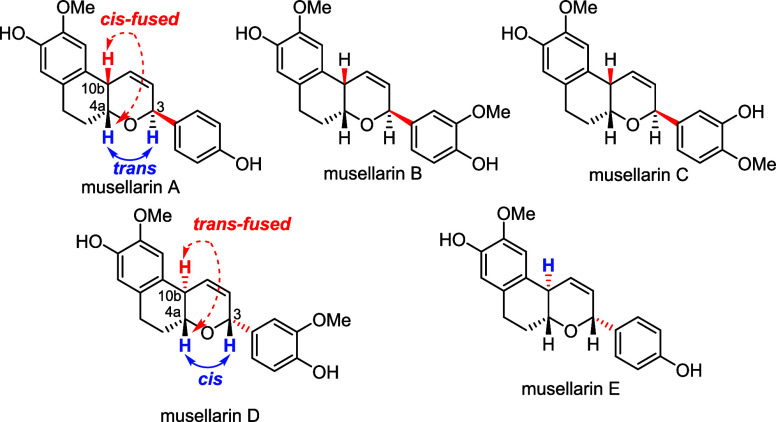
Molecular structures of musellarins A–E.

The potential biological activity, natural paucity, and intriguing molecular structures of musellarins triggered our interest in developing efficient synthetic strategies for their total synthesis. We have achieved the total synthesis of musellarins A–C in both racemic and enantiopure forms^5,6^ by exploiting Achmatowicz rearrangement for the construction of the central DHP core (Scheme 1a). Notably, the unfavorable (4a,3)*-trans-*configuration of DHP was forged by palladium-catalyzed Heck-Matsuda arylation, and the Friedel–Crafts cyclization delivered the tricyclic core with the desired (4a, 10b)-*cis*-fused configuration. Although this strategy was highly efficient and flexible (12 analogues of musellarins were prepared in 10–50 mg for biological studies), it was not applicable to the synthesis of musellarins D and E, which remains synthetically unsolved.

**Scheme 1:**
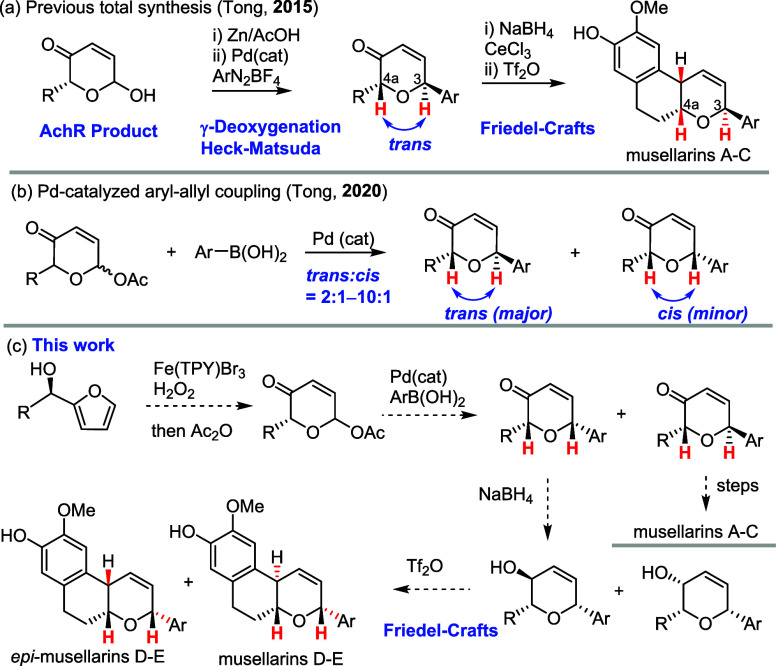
Previous Work and Our Collective Synthetic Strategy for Musellarins A–C and Musellarins D–E

In 2020, our group developed a new palladium-catalyzed C-aryl glycosylation where acetylated AchR products reacted efficiently with arylboronic acids under palladium catalysis (Scheme 1b).^7^ While the major aryl–allyl coupling products were 2,6-*trans*-configured dihydropyranones (when R is alkyl), we observed the minor diastereomeric products with a 2,6-*cis*-configuration. These findings sparked our interest in exploiting this aryl–allyl coupling reaction for collective total synthesis of not only musellarins A–C (from the major *trans*-configuration) but also musellarins D–E (from the minor *cis*-configuration) (Scheme 1c). The potential synthetic challenge for musellarins D–E might be derived from the Friedel–Crafts cyclization, which might result in favorable formation of *cis*-fused tricyclic products corresponding to *epi*-musellarins D–E. However, we believed that the stereochemistry of allylic alcohol might influence the stereochemical outcome of Friedel–Crafts cyclization and might be explored for *trans*-fused (4a, 10b) cyclization as commonly observed in the polyene cyclization.^8–12^

## Results and Discussion

Our synthesis began with the preparation of optically active furfuryl alcohol **1** by our reported 5-step method^6^ (Scheme 2). With enantiopure furfuryl alcohol **1** in hand, we carried out AchR reaction with our oxone/KBr (cat) green condition^13^ to provide AchR products, which was immediately acetylated with acetic anhydride (Ac_2_O) to deliver acetylated AchR product **2** (*dr*, 3:2) in 84% yields (gram scale) over two steps. It should be noted that stoichiometric NBS was employed for the AchR reaction of furfuryl alcohol **1** in our previous synthesis to produce compound **2** in a 90% yield. Subsequently, we found that our newly established system Fe­(TPY)­Br_3_(cat)/H_2_O_2_ ^14^ could affect the AchR reaction with comparable high yield (90%) within 15 min in a gram scale. Notably, only 2 mol % of Fe­(TPY)­Br_3_ was needed as the catalyst and water was the only stoichiometric byproduct, which greatly eased the purification and substantially improved the “greenness” of our synthesis.^15–18^ Next, we set out to examine the allyl–aryl coupling of **2** with aryl boronic acids (**3a**–**3c**) with our previously reported conditions (Pd_2_(dba)_3_/K_2_CO_3_ or PdCl_2_/KF). It was found that the coupling reaction with Pd_2_(dba)_3_/K_2_CO_3_ occurred with better yields (73–78%) in THF/H_2_O, although an inseparable mixture of diastereomers (*dr,* 1:0.9 or 1:0.8) was obtained. Nevertheless, these diastereomers were desirable, because they might be elaborated to musellarins A–C and musellarins D–E, respectively.

**Scheme 2:**
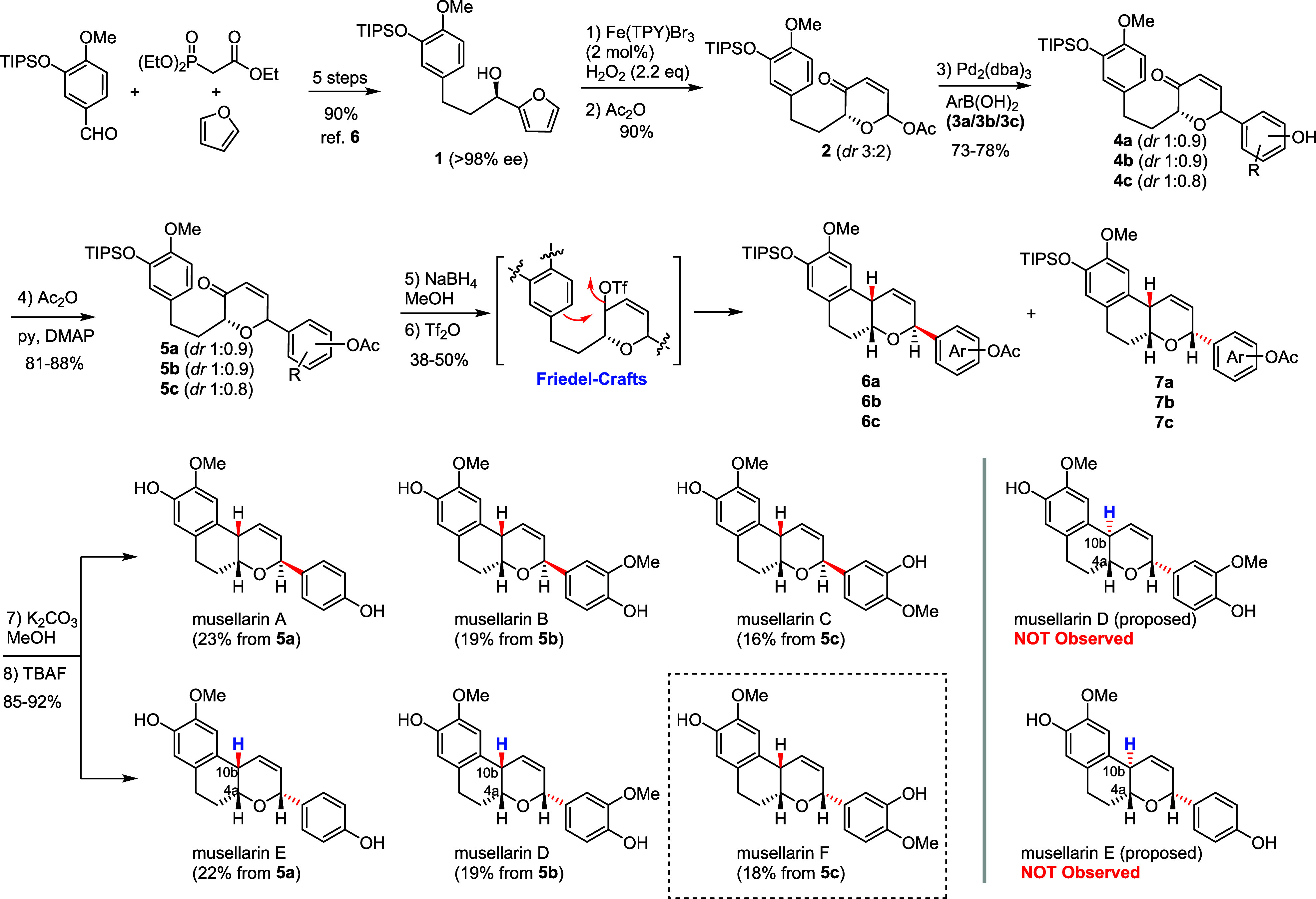
Asymmetric Collective Total Syntheses of Musellarins A–E

Acetylation of the phenolic alcohol of these diastereomers did not result in better separation of these diastereomers. Therefore, we moved forward to reduce the enones **5a**–**5c** with NaBH_4_ in methanol, and without surprise, four anticipated diastereomers were isolated as two inseparable pairs of diastereomers, which complicated their characterization. Luckily, it was unnecessary to separate these four diastereomers, because subsequent Friedel–Crafts cyclization of these diastereomers with triflic anhydride yielded only two separable diastereomers (**6a/7a**, **6b/7b**, and **6c/7c**), which upon global deprotection furnished the corresponding pair of musellarins (A/E, B/D, and C/F). It was noted that musellarin F was not reported in the literature. All spectral data of our synthetic musellarins were consistent with those reported for the natural musellarins by Zhao,^2^ which suggested that we successfully achieved the first total synthesis of musellarins D and E. However, we were skeptical about the relative stereochemistry of the natural musellarins D and E because their *trans-fused* configuration [(4a,10b)-*trans*] would not be favorably generated in the Friedel–Crafts cyclization.^19–21^ We suspected that the relative stereochemistry of musellarins D and E might be misassigned, which prompted us to re-examine their NMR data and designed further experiments to elucidate their relative stereochemistry.^22,23^

We first analyzed the NMR data of natural musellarins A and E and found that both compounds were reported to have the similar NOE enhancement^24^ between protons at C4a (4.2 ppm) and C10b (3.2–3.4 ppm), which was also confirmed by our NOE experiment (4.31% NOE for musellarin A and 5.57% NOE for musellarin E) (Figure 2). This finding suggested that musellarin E might have the identical *cis*-fused configuration [(4a,10b)-*cis*] as musellarin A^25^ (note: X-ray diffraction of a single crystal was reported for musellarin A). In addition, the NOE enhancement (4.18%) between protons at C4a (4.2 ppm) and C3 (5.1 ppm) of musellarin E substantiated the (4a,3)-*cis*-THP configuration.

**Figure 2:**
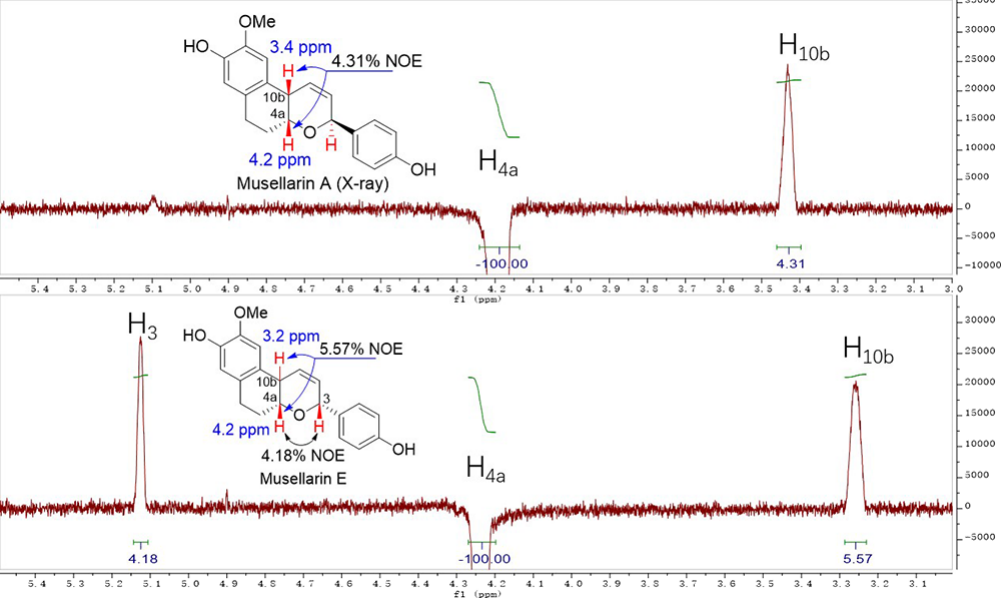
NOE spectra of musellarins A and E.

To further support the stereochemical revision of musellarin E, we carried out two chemical transformations of musellarins A and E, which differed only in C3 stereochemistry (Scheme 3a). First, treatment of either musellarin A or musellarin E with HCl/Et_2_O in methanol at room temperature^26^ for 15 min resulted in a mixture (1:1.8) of musellarins A and E, which were determined by in situ proton NMR. This C3 epimerization result consolidated our stereochemical revision of musellarin E as a C3 epimer of musellarin A. Second, we decided to eliminate the C3 stereochemistry by reductive cleavage of C3–O bond of musellarins A and E. It was discovered that the reductive hydrogenation of both musellarins A and E with a combination of Pd/C and Pd­(OH)_2_/C in HCl/MeOH resulted in efficient C3–O bond cleavage as well as alkene hydrogenation to produce the same reduction product **8**. Since the catalytic hydrogenation condition would be less likely to cause epimerization at C10b, we concluded that musellarins A and E differed only at C3 stereochemistry and therefore the proposed structure of musellarin E should be revised at C10b stereochemistry. Accordingly, the relative stereochemistry of musellarin D should be revised at the C10b as a C3 epimer of musellarin C, which was supported by our similar experiments (NOE, C3 epimerization, and reductive cleavage of C3–O bond) (see details in the SI). All synthetic musellarins A–F were subjected to cytotoxicity and anti-inflammatory studies. While they were not cytotoxic (>25 μM) toward RAW 264.7 cells, their anti-inflammatory activity was only moderate (>10 μM) with the LPS model (NO, IL-6, IL-1b, and TNF-a) (see details in the SI).

**Scheme 3:**
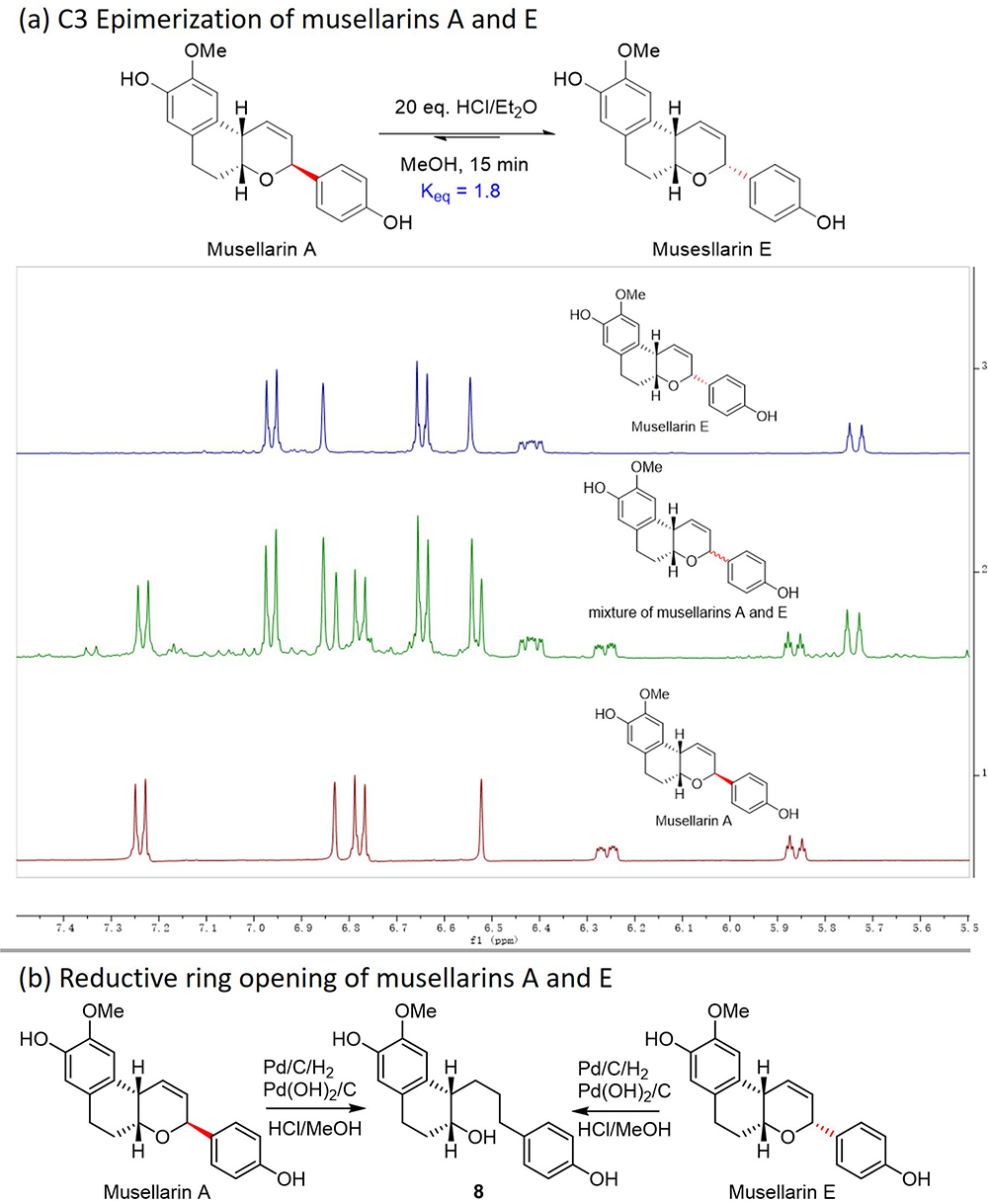
Chemical Transformations of Musellarins A and E

## Conclusions

In summary, we have developed a new synthetic strategy for collective total synthesis of musellarins A–E in 8 steps from known furfuryl alcohol **1** (13 steps from commercially available materials), featuring green Fe­(TPY)­Br_3_-catalyzed AchR to construct DHP ring, palladium-catalyzed arylation of AchR products with aryl boronic acids, and Friedel–Crafts cyclization. Notably, the total syntheses of musellarins D and E are achieved for the first time, which enables us to recognize the stereochemical misassignments and revise their stereochemistry. Three experiments (NOE, C3 epimerization, and reductive cleavage of the C3–O bond) were designed and performed to support our revision of C10b stereochemistry for musellarins D and E. Additionally, this work represents the first synthetic applications of two new methodologies established in our lab: Fe­(TPY)­Br_3_-catalyzed AchR and palladium-catalyzed arylation of AchR products with aryl boronic acids, in the total synthesis of natural products. All synthetic musellarins are not cytotoxic, with moderate anti-inflammatory effects.

## Supplementary Material



## Data Availability

The data underlying this study are available in the published article and its online Supporting Information.

## Notes

Experimental details, procedures, characterization data, and NMR spectra of all new compounds (PDF)
