# Supplementary material for: Collective Asymmetric Total Syntheses of Musellarins A–E
Source: J Org Chem. 2025 Nov 28;90(49):17528–32. doi: 10.1021/acs.joc.5c02575 (PMC12706788; doi:10.1021/acs.joc.5c02575)
Supplement: Supplementary file 1 [file jo5c02575_si_001.pdf]

*Supporting Information*

# Collective Asymmetric Total Syntheses of Musellarins A-E

Yichen Liu[a], Yanghui Ou[b], Vincent Chang[a], Hongliang Yao[b]*, and Rongbiao Tong[a]*

[a] *Department of Chemistry, The Hong Kong University of Science and Technology, Clearwater Bay, Kowloon, Hong Kong, China*

[b] *Guangdong Key Laboratory of Animal Conservation and Resource Utilization, Institute of Zoology, Guangdong Academy of Sciences, Guangzhou, Guangdong, 510260, China*

## Table of content


| | |
|---|---|
| 1. General Information | S-2 |
| 2. Achamatowicz Rearrangement and Acetylation | S-3 |
| 3. Arylation of Achmatowicz Rearrangement Product with Arylboronic Acids | S-4 |
| 4. Acetylation of Arylation products | S-5 |
| 5. Ketone reduction and Friedel-Crafts Cyclization | S-7 |
| 6. Deacetylation with K2CO3/MeOH and Desilylation with TBAF | S-10 |
| 7. Spectroscopic Data Comparison of Our Synthetic Musellarin E with Those Reported for Natural Musellarin E | S-12 |
| 8. Spectroscopic Data Comparison of Our Synthetic Musellarin D with Those Reported for Natural Musellarin D | S-14 |
| 9. Hydrogenation of Musellarins | S-15 |
| 10. Epimerization of Musellarins | S-16 |
| 11. NOE spectra comparison of Musellarins A and E, B and D | S-18 |
| 12. Synthesis of dibenzofuran analogues | S-19 |
| 13. Anti-inflammatory tests of Musellarins A-F and analogues | S-20 |
| 14. $^{1}H$ and $^{13}C\{^{1}H\}$ NMR spectra | S-23 |

**General Information:** Reactions were carried out in oven or flame-dried glassware under a nitrogen atmosphere, unless otherwise noted. Tetrahydrofuran (THF) was freshly distilled before use from sodium using benzophenone as indicator. Dichloromethane (DCM) was freshly distilled before use from calcium hydride ($CaH_2$). All other anhydrous solvents were dried over 3Å or 4Å molecular sieves. Solvents used in workup, extraction and column chromatography were used as received from commercial suppliers without prior purification. Reactions were magnetically stirred and monitored by thin layer chromatography (TLC, 0.25 mm) on Merck pre coated silica gel plates. Flash chromatography was performed with silica gel 60 (particle size 0.040–0.062 mm) supplied by Grace. Infrared spectra were collected on a Bruker model TENSOR27 spectrophotometer. $^{1}H$ and $^{13}C$ NMR spectra were recorded on a Bruker AV-400 spectrometer (400 MHz for $^{1}H$, 100 MHz for $^{13}C$). Chemical shifts are reported in parts per million (ppm) as values relative to the internal chloroform (7.26 ppm for $^{1}H$ and 77.16 ppm for $^{13}C$), benzene (7.16 ppm for $^{1}H$ and 128.06 ppm for 13C), methanol (3.31 ppm for $^{1}H$ and 49.00 ppm for $^{13}C$), acetone (2.09 ppm for $^{1}H$ and 30.60 ppm for $^{13}C$). Abbreviations for signal coupling are as follows: s, singlet; d, doublet; t, triplet; q, quartet; m, multiplet. Optical rotations were measured on a JASCO Perkin-Elmer model P-2000 polarimeter. High resolution mass spectra were measured at the Hong Kong University of Science and Technology Mass Spectrometry Service Center on either an Agilent GC/MS 5975C System or an API QSTAR XL System.

## EXPERIMENTAL SECTION

### Achamatowicz Rearrangement and Acetylation

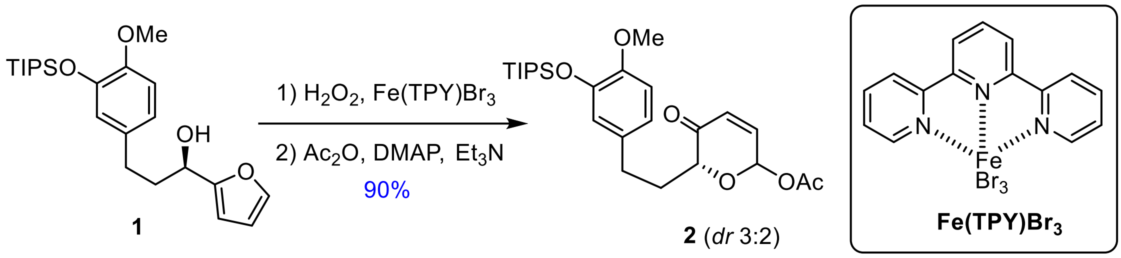


To a stirred solution of compound **1** (prepared as literature[1], 2 g, 4.94 mmol, 1.0 equiv) in THF/$H_2O$ (3/1, 25 mL) were added Fe(TPY)$Br_3$ (prepared as literature[2], 52.3 mg, 99 µmol, 2 mol%) and $H_2O_2$ (30 wt%, 0.849 mL, 10.8 mmol, 2.2 equiv) at rt. After completion of the addition, the reaction mixture was allowed to stir at rt for 15 min. The reaction was quenched by dilute aqueous $Na_2S_2O_3$ solution (0.1 M, 50 mL) and ethyl acetate (50 mL). The organic fractions were collected, and the aqueous phase was extracted with ethyl acetate (2 × 50 mL). The combined organic fractions were washed with brine, dried over $Na_2SO_4$, filtered, and concentrated under reduced pressure. The crude product was used for next step without further purification.

To a stirred solution of crude product obtained above in $CH_2Cl_2$ (10 mL) were added acetic anhydride ($Ac_2O$, 0.757 g, 7.41 mmol, 1.5 equiv), $Et_3N$ (1.00 g, 9.88 mmol, 2 equiv) and 4-dimethylaminopyridine (DMAP, 0.121 g, 0.988 mmol, 0.2 equiv) at 0 °C. The reaction mixture was stirred at 0 °C for 15 min. The reaction was quenched by addition of saturated aqueous $NH_4Cl$ (10 mL) and extracted with $CH_2Cl_2$ (3 x 10 mL). The combined organic fractions were washed with brine, dried over $Na_2SO_4$, filtered and concentrated under reduced pressure. The resulting residue was purified by flash column chromatography on silica gel (hexane/EtOAc = 8:1) to afford the desired product **2** (2.06 g, 4.45 mmol, 90% yield over two steps) as a 3:2 diastereomeric mixture.

$[\alpha]_D^{20}$ = +37.8 (c 1, MeOH).

**$^1$H NMR** for the major isomer (400 MHz, $C_6D_6$) δ 6.96 – 6.94 (m, 1H), 6.73 (dd, J = 8.4, 2.4 Hz, 1H), 6.56 (d, J = 8.0 Hz, 1H), 6.36 – 6.32 (m, 1H), 6.08 – 6.03 (m, 1H), 5.77 (d, J = 10.4 Hz, 1H), 4.00 (dd, J = 9.6, 4.4 Hz, 1H), 3.36 (s, 3H), 2.72 – 2.64 (m, 2H), 2.23 – 1.93 (m, 2H), 1.62 (s, 3H), 1.33 – 1.24 (m, 3H), 1.17 – 1.14 (m, 18H).

**$^{13}$C{$^1$H} NMR** for the major isomer (100 MHz, $C_6D_6$) δ 194.8, 168.6, 149.8, 146.0, 143.0, 141.3, 133.7, 128.5, 121.8, 112.6, 88.1, 78.4, 55.1, 34.4, 30.7, 20.5, 18.3 (6 x C), 13.4 (3 x C).

**$^1$H NMR** for the minor isomer (400 MHz, $C_6D_6$) δ 6.96 – 6.94 (m, 1H), 6.70 (dd, J = 8.0, 2.0 Hz, 1H), 6.52 (d, J = 8.4 Hz, 1H), 6.36 – 6.32 (m, 1H), 6.08 – 6.03 (m, 1H), 5.77 (d, J = 10.4 Hz, 1H), 4.29 (dd, J = 8.4, 3.2 Hz, 1H), 3.35 (s, 3H), 2.72 – 2.64 (m, 2H), 2.42 – 2.33 (m, 1H), 2.23 – 1.93 (m, 1H), 1.66 (s, 3H), 1.33 – 1.24 (m, 3H), 1.17 – 1.14 (m, 18H).

**$^{13}$C{$^1$H} NMR** for the minor isomer (100 MHz, $C_6D_6$) δ 195.0, 168.8, 149.7, 146.0, 141.3, 133.70, 128.4, 121.3, 112.5, 86.9, 74.6, 55.1, 31.6, 30.2, 20.42, 18.2 (6 x C), 13.4 (3 x C).

**HRMS** (TOF, ESI$^+$) *m/z* calculated for $C_{25}H_{38}O_6SiNa$, $[M+Na]^+$ 485.2335, found 485.2341.

---

[1] Li, Z.; Ip, F. C. F.; Ip, N. Y.; Tong, R. *Chem. Eur. J.* **2015**, *21* (31), 11152-11157.

[2] Zhao, G.; Dong, H.; Xue, K.; Lou, S.; Qi, R.; Zhang, X.; Cao, Z.; Qin, Q.; Yi, B.; Lei, H.; et al. *Sci. Adv.* **2024**, *10* (49), eadq0028.

**Arylation of Achmatowicz Rearrangement Product with Arylboronic Acids**

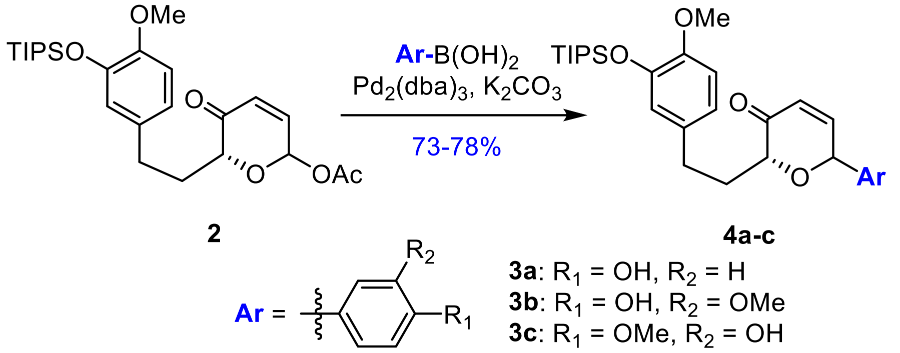


To a stirred solution of pyranulose acetate **2** (200 mg, 0.432 mmol, 1.0 equiv) in 2.5 mL of dry tetrahydrofuran (THF) under $N_2$ were added $ArB(OH)_2$ **3a**, **3b**, or **3c** (1.728 mmol, 4.0 equiv) and $K_2CO_3$ (239 mg, 1.728 mmol, 4.0 equiv) at rt. After the mixture was stirred for 5 min, $Pd_2(dba)_3$ (9.9 mg, 0.0108 mmol, 2.5 mol %) was added at rt. The reaction mixture was allowed to stir at rt for 6 - 12 h until completion of the reaction as determined by TLC analysis. The reaction mixture was then filtered through a silica pad with EtOAc. The filtrate collected was concentrated under reduced pressure. The resulting residue was purified by flash column chromatography (hexane/EtOAc = 8:1 to 4:1) to afford the desired product **4a-c**.

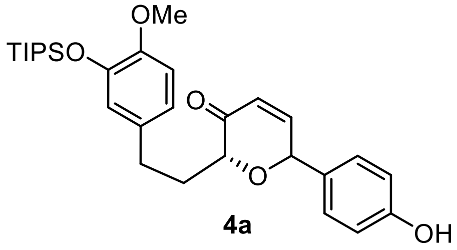


**Compound 4a** was obtained in 75% yield (162 mg, 0.325 mmol, *dr* 1:0.9) from **2** and **3a** as a yellowish oil. **$^1$H NMR** for the *cis* isomer (400 MHz, $C_6D_6$) δ 7.08 (d, J = 8.5 Hz, 2H), 6.94 (d, J = 2.0 Hz, 1H), 6.77 (d, J = 8.5 Hz, 2H), 6.72 (dd, J = 8.2, 2.0 Hz, 1H), 6.57 (d, J = 8.3 Hz, 1H), 6.36 (dd, J = 10.4, 3.3 Hz, 1H), 5.91 (dd, J = 10.2, 2.5 Hz, 1H), 4.76 (q, J = 2.1 Hz, 1H), 3.83 (ddd, J = 8.5, 3.9, 2.2 Hz, 1H), 3.40 (s, 3H), 2.83 – 2.69 (m, 2H), 2.53 – 2.43 (m, 1H), 2.20 – 2.05 (m, 1H), 1.31 – 1.22 (m, 3H), 1.16 – 1.13 (m, 18H).

**$^{13}$C{$^1$H} NMR** for the *cis* isomer (100 MHz, $C_6D_6$) δ 196.9, 157.3, 151.5, 149.7, 146.0, 134.5, 131.5, 130.0, 128.9 (2 x C), 126.5, 121.7, 121.4, 115.9 (2 x C), 112.7, 79.6, 76.6, 55.3, 31.9, 30.6, 18.3 (6 x C), 13.4 (3 x C).

**$^1$H NMR** for the *trans* isomer (400 MHz, $C_6D_6$) δ 6.97 (d, J = 8.4 Hz, 2H), 6.96 (dd, J = 2.0 Hz, 1H), 6.67 (d, J = 8.8 Hz, 2H), 6.57 (dd, J = 8.4, 2.0 Hz, 1H), 6.50 (d, J = 8.2 Hz, 1H), 6.31 (dd, J = 10.2, 1.6 Hz, 1H), 5.97 (dd, J = 10.4, 1.9 Hz, 1H), 5.05 (t, J = 2.4 Hz, 1H), 4.08 (dd, J = 9.1, 3.9 Hz, 1H), 3.38 (s, 3H), 2.78 – 2.71 (m, 1H), 2.64 – 2.57 (m, 1H), 2.29 – 2.14 (m, 1H), 2.22 – 2.06 (m, 1H), 1.33 – 1.25 (m, 3H), 1.17 (d, J = 2.4 Hz, 18H).

**$^{13}$C{$^1$H} NMR** for the *trans* isomer (100 MHz, $C_6D_6$) δ 196.6, 157.1, 149.62, 149.58, 145.9, 134.2, 130.0 (2 x C), 128.5, 126.2, 121.8, 121.2, 115.9 (2 x C), 112.7, 75.6, 72.7, 55.3, 31.8, 31.0, 18.3 (6 x C), 13.4 (3 x C).

**HRMS** (TOF, ESI$^-$) *m/z* calculated for $C_{29}H_{39}O_5Si$, $[M-H]^-$ 495.2567, found 495.2566.

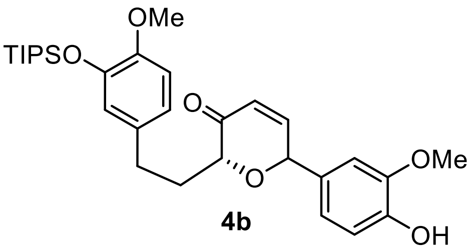


**4b**

**Compound 4b** was obtained in 78% yield (177 mg, 0.335 mmol, *dr* 1:0.9) from **2** and **3b** as a yellowish oil.
**$^1$H NMR** (400 MHz, $C_6D_6$) δ 7.01 (s, 1H), 7.00 (d, J = 6.2 Hz, 1H), 6.95 (d, J = 2.2 Hz, 1H), 6.93 (d, J = 8.1 Hz, 1H), 6.74 (dd, J = 8.2, 2.2 Hz, 1H), 6.71 (d, J = 1.9 Hz, 1H), 6.68 (dd, J = 6.9, 1.9 Hz, 1H), 6.67 (s, 1H), 6.59 – 6.54 (m, 3H), 6.48 (d, J = 8.2 Hz, 1H), 6.38 (dd, J = 10.4, 3.3 Hz, 1H), 6.33 (dd, J = 10.2, 1.6 Hz, 1H), 6.00 (dd, J = 10.3, 1.9 Hz, 1H), 5.96 (dd, J = 10.2, 2.5 Hz, 1H), 5.70 (s, 1H), 5.68 (s, 1H), 5.08 (dd, J = 3.4, 1.9 Hz, 1H), 4.78 (q, J = 2.1 Hz, 1H), 4.12 (dd, J = 9.1, 3.9 Hz, 1H), 3.88 (ddd, J = 8.2, 3.8, 1.9 Hz, 1H), 3.37 (s, 3H), 3.37 (s, 3H), 3.17 (s, 3H), 3.17 (s, 3H), 2.89 – 2.73 (m, 3H), 2.67 – 2.50 (m, 2H), 2.30 – 2.08 (m, 3H), 1.32 – 1.22 (m, 6H), 1.16 (d, J = 2.9 Hz, 18H), 1.15 (d, J = 2.1 Hz, 18H).
**$^{13}$C{$^1$H} NMR** (100 MHz, $C_6D_6$) δ 195.7, 195.5, 150.9, 149.7, 149.6, 148.9, 147.23, 147.19, 146.8, 146.7, 146.0 (2 x C), 134.5, 134.2, 131.7, 128.9, 126.6, 126.4, 121.72, 121.66, 121.6, 121.4, 121.1, 120.7, 114.8, 114.6, 112.7, 112.4, 111.0, 109.9, 79.8, 77.0, 75.8, 72.9, 55.4, 55.3, 55.23, 55.18, 32.0, 31.8, 31.1, 30.7, 18.27 (6 x C), 18.26 (6 x C), 13.40 (3 x C), 13.39 (3 x C).
**HRMS** (TOF, ESI$^+$) *m/z* calculated for $C_{30}H_{42}O_6SiNa$, $[M+Na]^+$ 549.2648, found 549.2656.

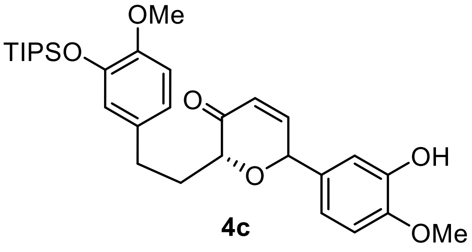


**4c**

**Compound 4c** was obtained in 73% yield (166 mg, 0.315 mmol, *dr* 1:0.8) from **2** and **3c** as a yellowish oil.
**$^1$H NMR** (400 MHz, $C_6D_6$) δ 7.05 (d, J = 2.1 Hz, 1H), 7.02 (d, J = 2.1 Hz, 1H), 7.01 (d, J = 2.1 Hz, 1H), 6.97 (d, J = 2.2 Hz, 1H), 6.80 (dd, J = 8.3, 2.1 Hz, 1H), 6.73 (dd, J = 8.2, 2.2 Hz, 1H), 6.69 (dd, J = 8.3, 2.2 Hz, 1H), 6.63 (dd, J = 8.1, 2.2 Hz, 1H), 6.56 (d, J = 8.2 Hz, 1H), 6.49 (d, J = 8.3 Hz, 1H), 6.43 (d, J = 8.3 Hz, 1H), 6.39 (d, J = 8.2 Hz, 1H), 6.31 (dd, J = 10.3, 3.3 Hz, 1H), 6.28 (dd, J = 10.2, 1.5 Hz, 1H), 5.94 (dd, J = 10.4, 2.0 Hz, 1H), 5.88 (dd, J = 10.2, 2.6 Hz, 1H), 5.49 (s, 1H), 5.46 (s, 1H), 5.04 (t, J = 2.4 Hz, 1H), 4.75 (q, J = 2.1 Hz, 1H), 4.13 (dd, J = 9.0, 4.0 Hz, 1H), 3.84 (ddd, J = 8.3, 3.8, 2.0 Hz, 1H), 3.37 (s, 3H), 3.36 (s, 3H), 3.15 (s, 3H), 3.12 (s, 3H), 2.85 – 2.74 (m, 3H), 2.68 – 2.59 (m, 1H), 2.57 – 2.47 (m, 1H), 2.28 – 2.06 (m, 3H), 1.34 – 1.25 (m, 6H), 1.18 (d, J = 4.7 Hz, 18H), 1.17 (d, J = 4.4 Hz, 18H).
**$^{13}$C{$^1$H} NMR** (100 MHz, $C_6D_6$) δ 195.9, 195.7, 150.8, 149.60, 149.58, 149.0, 147.2, 147.0, 146.6, 146.6, 145.9 (2 x C), 134.5, 134.3, 133.2, 130.6, 126.6, 126.3, 121.8, 121.7, 121.4, 121.2, 119.9, 118.8, 115.2, 114.1, 112.7, 112.5, 111.0, 110.8, 79.6, 76.5, 75.9, 72.5, 55.50, 55.48, 55.25, 55.23, 32.0, 31.8, 31.0, 30.6, 18.28 (6 x C), 18.26 (6 x C), 13.40 (3 x C), 13.38 (3 x C).
**HRMS** (TOF, ESI$^-$) *m/z* calculated for $C_{30}H_{41}O_6Si$, $[M-H]^-$ 525.2672, found 525.2667.

**Acetylation of Arylation products**

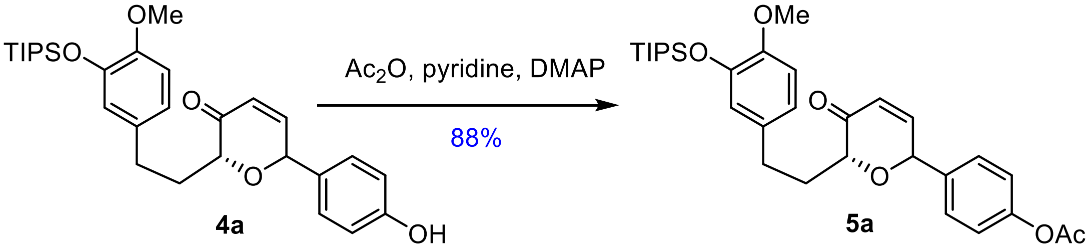

To a stirred solution of **4a** (149 mg, 0.3 mmol, 1.0 equiv) in $CH_2Cl_2$ (10 mL) were added acetic anhydride ($Ac_2O$, 36.8 mg, 0.36 mmol, 1.2 equiv), pyridine (26.1 mg, 0.33 mmol, 1.1 equiv) and 4-dimethylaminopyridine (DMAP, 3.67 mg, 30 μmol, 0.1 equiv) at 0 °C. The reaction mixture was stirred at rt for 0.5 h. The reaction was quenched by addition of saturated aqueous $NH_4Cl$ (10 mL) and extracted with $CH_2Cl_2$ (3 × 5 mL). The combined organic fractions were washed with brine, dried over $Na_2SO_4$, filtered and concentrated under reduced pressure. The resulting crude acetylated product **5a** were used without purification for next step, or purified by flash column chromatography (hexane/EtOAc = 8:1 to 6:1).

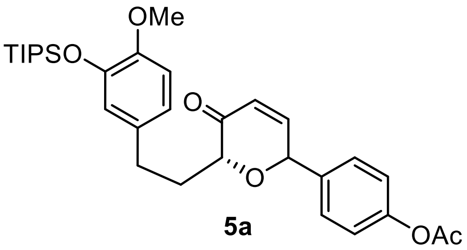

**5a**

**Compound 5a** was obtained in 88% yield (141 mg, 0.263 mmol, *dr* 1:0.9) from **4a** as a yellowish oil.
**$^1$H NMR** (400 MHz, $CDCl_3$) δ 7.43 (d, J = 8.6 Hz, 2H), 7.41 (d, J = 8.5 Hz, 2H), 7.15 (d, J = 8.6 Hz, 2H), 7.14 (d, J = 8.5 Hz, 2H), 7.10 (dd, J = 10.4, 3.0 Hz, 1H), 6.98 (dd, J = 10.2, 1.6 Hz, 1H), 6.77 – 6.72 (m, 3H), 6.71 (d, J = 2.2 Hz, 1H), 6.68 (d, J = 8.2 Hz, 1H), 6.57 (dd, J = 8.2, 2.1 Hz, 1H), 6.21 (dd, J = 10.4, 2.1 Hz, 1H), 6.17 (dd, J = 10.2, 2.6 Hz, 1H), 5.49 (t, J = 2.6 Hz, 1H), 5.32 (q, J = 2.1 Hz, 1H), 4.11 – 4.07 (m, 1H), 4.05 (ddd, J = 8.5, 3.7, 2.0 Hz, 1H), 3.77 (s, 3H), 3.75 (s, 3H), 2.77 – 2.62 (m, 3H), 2.62 – 2.52 (m, 1H), 2.32 (s, 3H), 2.32 (s, 3H), 2.35 – 2.25 (m, 1H), 2.10 – 1.95 (m, 3H), 1.30 – 1.18 (m, 6H), 1.09 (d, J = 3.0 Hz, 18H), 1.08 (d, J = 2.7 Hz, 18H).
**$^{13}$C{$^1$H} NMR** (100 MHz, $CDCl_3$) δ 196.4, 196.2, 169.5, 169.4, 151.0, 150.8, 150.4, 149.3, 149.2, 148.9, 145.5, 145.4, 136.8, 134.8, 133.9, 133.6, 129.2 (2 x C), 128.3 (2 x C), 127.0, 126.5, 122.11 (2 x C), 122.08 (2 x C), 121.35, 121.26, 121.1, 120.9, 112.3, 112.2, 79.5, 76.4, 76.2, 71.9, 55.7 (2 x C), 31.3, 31.2, 30.7, 30.2, 21.22, 21.21, 18.1 (6 x C), 18.0 (6 x C), 13.02 (3 x C), 13.00 (3 x C).
**HRMS** (TOF, ESI$^+$) *m/z* calculated for $C_{31}H_{42}O_6SiNa$, $[M+Na]^+$ 561.2648, found 561.2646.

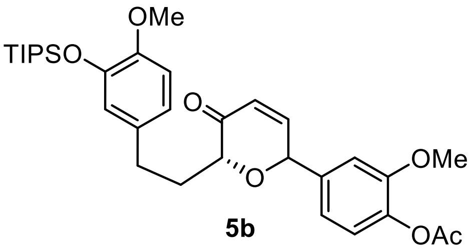

**5b**

**Compound 5b** was obtained in 85% yield (146 mg, 0.256 mmol) from **4b** as a yellowish oil, following the identical procedure for the synthesis of **5a**.
**$^1$H NMR** for the *cis* isomer (400 MHz, $C_6D_6$) δ 7.03 – 6.97 (m, 2H), 6.81 (s, 1H), 6.79 – 6.71 (m, 2H), 6.58 (d, J = 7.9 Hz, 1H), 6.21 (dd, J = 10.1, 1.5 Hz, 1H), 5.92 (dd, J = 10.4, 1.5 Hz, 1H), 4.77 (br s, 1H), 3.88 (ddd, J = 8.3, 3.8, 1.8 Hz, 1H), 3.37 (s, 3H), 3.30 (s, 3H), 2.91 – 2.75 (m, 2H), 2.67 – 2.52 (m, 1H), 2.27 – 2.16 (m, 1H), 1.91 (s, 3H), 1.33 – 1.23 (m, 3H), 1.16 (d, J = 7.3 Hz, 18H).
**$^{13}$C{$^1$H} NMR** for the *cis* isomer (100 MHz, $C_6D_6$) δ195.1, 168.1, 152.1, 150.3, 149.7, 146.0, 140.6, 138.4, 134.4, 126.7, 123.4, 121.7, 121.4, 119.3, 112.7, 111.2, 79.8, 76.6, 55.4, 55.2, 32.0, 30.7, 20.3, 18.3 (6 x C), 13.4 (3 x C).
**$^1$H NMR** for the *trans* isomer (400 MHz, $C_6D_6$) δ 6.97 – 6.92 (m, 2H), 6.78 (s, 1H), 6.61 (dd, J = 8.4, 1.6 Hz, 1H), 6.58 (s, 2H), 6.31 (dd, J = 10.4, 3.2 Hz, 1H), 5.97 (dd, J = 10.4, 1.9 Hz, 1H), 5.05 (t, J = 2.7 Hz, 1H), 4.12 (dd, J = 9.2, 3.9 Hz, 1H), 3.41 (s, 3H), 3.30 (s, 3H), 2.78 – 2.71 (m, 1H), 2.65 – 2.57 (m, 1H), 2.23 – 2.15 (m, 1H), 2.12 – 2.03 (m, 1H), 1.91 (s, 3H), 1.34 – 1.23 (m, 3H), 1.16 (d, J = 7.3 Hz, 18H).
**$^{13}$C{$^1$H} NMR** for the *trans* isomer (100 MHz, $C_6D_6$) δ 195.3, 168.1, 152.2, 149.7, 148.3, 146.0,

140.8, 136.3, 134.1, 126.4, 123.2, 121.8, 121.1, 120.3, 112.6, 112.3, 76.2, 72.4, 55.4, 55.2, 31.8, 31.0, 20.3, 18.3 (6 x C), 13.4 (3 x C).

**HRMS** (TOF, ESI$^+$) *m/z* calculated for $C_{32}H_{44}O_7SiNa$, $[M+Na]^+$ 591.2754, found 591.2752.

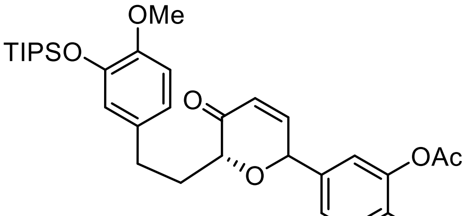


**5c**

**Compound 5c** was obtained in 81% yield (138 mg, 0.242 mmol) from **4c** as a yellowish oil, following the identical procedure for the synthesis of **5a**.

**$^1$H NMR** for the *cis* isomer (400 MHz, $C_6D_6$) δ 7.09 (d, J = 2.2 Hz, 1H), 7.03 – 7.00 (m, 1H), 6.73 (dd, J = 8.2, 2.1 Hz, 1H), 6.57 (d, J = 8.3 Hz, 1H), 6.54 (d, J = 8.3 Hz, 1H), 6.21 (dd, J = 10.2, 1.6 Hz, 1H), 5.89 (dd, J = 10.2, 2.6 Hz, 1H), 4.76 (q, J = 2.1 Hz, 1H), 3.85 (ddd, J = 8.2, 3.8, 1.9 Hz, 1H), 3.37 (s, 3H), 3.27 (s, 3H), 2.87 – 2.72 (m, 2H), 2.58 – 2.48(m, 1H), 2.23 – 2.08 (m, 1H), 1.92 (s, 3H), 1.33 – 1.22 (m, 3H), 1.16 (d, J = 3.0 Hz, 18H).

**$^{13}$C{$^1$H} NMR** for the *cis* isomer (100 MHz, $C_6D_6$) δ 195.2, 168.1, 151.9, 150.1, 149.6, 148.2, 140.7, 134.5, 132.5, 126.8, 125.4, 122.2, 121.7, 121.4, 112.7, 112.5, 79.7, 76.1, 55.5, 55.2, 32.0, 30.7, 20.3, 18.3 (6 x C), 13.4 (3 x C).

**$^1$H NMR** for the *trans* isomer (400 MHz, $C_6D_6$) δ 7.03 (d, J = 2.2 Hz, 1H), 6.96 (d, J = 2.2 Hz, 1H), 6.91 (dd, J = 8.4, 2.2 Hz, 1H), 6.61 (dd, J = 8.2, 2.1 Hz, 1H), 6.52 (d, J = 8.5 Hz, 1H), 6.50 (d, J = 8.5 Hz, 1H), 6.27 (dd, J = 10.4, 3.3 Hz, 1H), 5.94 (dd, J = 10.4, 1.9 Hz, 1H), 5.02 (t, J = 2.6 Hz, 1H), 4.10 (dd, J = 9.1, 3.9 Hz, 1H), 3.39 (s, 3H), 3.29 (s, 3H), 2.80 – 2.73 (m, 1H), 2.69 – 2.57 (m, 1H), 2.28 – 2.19 (m, 1H), 2.17 – 2.01 (m, 1H), 1.92 (s, 3H), 1.34 – 1.23 (m, 3H), 1.18 (d, J = 3.3 Hz, 31H).

**$^{13}$C{$^1$H} NMR** for the *trans* isomer (100 MHz, $C_6D_6$) δ 195.4, 168.1, 152.0, 149.7, 148.2, 145.9, 140.7, 134.2, 129.9, 126.55, 126.48, 123.4, 121.8, 121.1, 112.6, 112.3, 75.9, 72.1, 55.5, 55.2, 31.7, 31.0, 20.3, 18.3 (6 x C), 13.4 (3 x C).

**HRMS** (TOF, ESI$^+$) *m/z* calculated for $C_{32}H_{44}O_7SiNa$, $[M+Na]^+$ 591.2754, found 591.2750.

**Ketone reduction and Friedel-Crafts Cyclization**

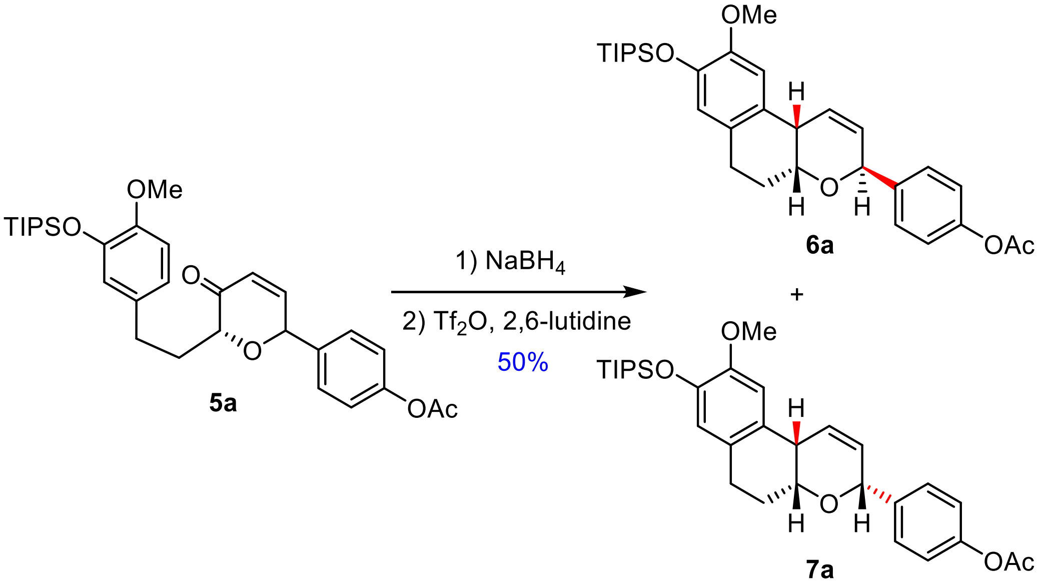

To a stirred solution of enone 5**a** (162 mg, 0.3 mmol, 1.0 equiv) in methanol (5 mL) at 0 °C was added $NaBH_4$ (13.6 mg, 0.36 mmol, 1.2 equiv). The reaction mixture was stirred at rt for 1 h. The reaction was quenched by addition of saturated aqueous $NH_4Cl$ (10 mL) and extracted with $CH_2Cl_2$ (3 × 5 mL). The combined organic fractions were washed with brine, dried over $Na_2SO_4$, filtered and concentrated under reduced pressure.

To a stirred solution of the crude reduction product in anhydrous $CH_2Cl_2$ (5 mL) were added 2,6-lutidine (161 mg, 1.5 mmol, 5.0 equiv), trifluoromethanesulfonic anhydride ($Tf_2O$, 254 mg, 0.9 mmol, 3.0 equiv) at -78 °C. The reaction mixture was stirred at -78 °C for 4 h. The reaction was quenched by addition of saturated aqueous $NH_4Cl$ (10 mL) and extracted with $CH_2Cl_2$ (3 × 5 mL). The combined organic fractions were washed with brine, dried over $Na_2SO_4$, filtered and concentrated under reduced pressure. The resulting crude cyclized products **6a** and **7a** were used without purification for next step, or separated by flash column chromatography (hexane/dichloromethane = 1:1).

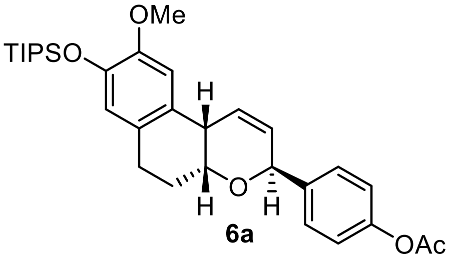

**6a**

**Compound 6a** was obtained in 26% yield (40 mg, 76.5 μmol) from **5a** as a yellowish oil.

$[\alpha]_D^{20}$ = -102.0 (c 0.20, MeOH).

**$^1$H NMR** (400 MHz, $CDCl_3$) δ 7.45 (d, J = 8.5 Hz, 2H), 7.09 (d, J = 8.6 Hz, 2H), 6.70 (s, 1H), 6.59 (s, 1H), 6.19 (ddd, J = 10.3, 3.8, 2.1 Hz, 1H), 5.89 (dt, J = 10.3, 2.5 Hz, 1H), 5.19 (q, J = 2.4 Hz, 1H), 4.24 (ddd, J = 8.3, 5.0, 3.0 Hz, 1H), 3.79 (s, 3H), 3.47 (br s, 1H), 2.89 – 2.82 (m, 1H), 2.68 – 2.58 (m, 1H), 2.31 (s, 3H), 2.17 – 2.07 (m, 1H), 1.93 – 1.84 (m, 1H), 1.30 – 1.20 (m, 3H), 1.09 (d, J = 7.1 Hz, 18H).

**$^{13}$C{$^1$H} NMR** (100 MHz, $CDCl_3$) δ 169.6, 150.3, 149.6, 144.0, 139.0, 130.1, 129.6, 129.0, 128.5, 127.3, 121.6, 120.2, 112.4, 71.8, 69.0, 56.0, 37.2, 26.3, 26.1, 21.3, 18.1 (6 x C), 13.1 (3 x C).

**HRMS** (TOF, ESI$^+$) *m/z* calculated for $C_{31}H_{42}O_5SiNa$, $[M+Na]^+$ 545.2699, found 545.2701.

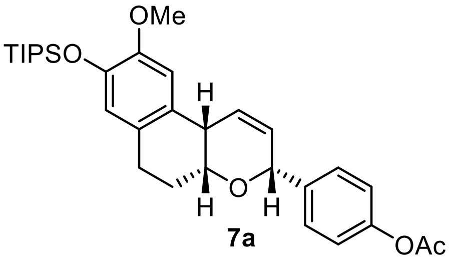

**7a**

**Compound 7a** was obtained in 24% yield (37 mg, 70.7 μmol) from **5a** as a yellowish oil.

$[\alpha]_D^{20}$ = -247.4 (c 0.23, MeOH).

**$^1$H NMR** (400 MHz, $CDCl_3$) δ 7.13 (d, J = 8.5 Hz, 2H), 6.93 (d, J = 8.5 Hz, 2H), 6.72 (s, 1H), 6.63 (s, 1H), 6.36 (ddd, J = 10.0, 6.0, 2.1 Hz, 1H), 5.75 (dt, J = 10.1, 1.6 Hz, 1H), 5.22 (q, J = 2.3 Hz, 1H), 4.24 (q, J = 3.5 Hz, 1H), 3.78 (s, 3H), 3.27 (br s, 1H), 3.00 (ddd, J = 16.7, 12.4, 5.2 Hz, 1H), 2.48 (ddd, J = 16.0, 5.6, 2.9 Hz, 1H), 2.26 (s, 3H), 2.16 – 2.09 (m, 1H), 1.94 – 1.84 (m, 1H), 1.32 – 1.21 (m, 3H), 1.11 (d, J = 6.9 Hz, 18H).

**$^{13}$C{$^1$H} NMR** (100 MHz, $CDCl_3$) δ 169.6, 150.2, 149.3, 143.5, 139.5, 130.4, 130.1, 128.5, 128.2 (2 x C), 127.6, 121.5 (2 x C), 120.5, 111.9, 76.8, 70.8, 55.9, 36.3, 28.6, 23.9, 21.3, 18.1 (6 x C), 13.0 (3 x C).

**HRMS** (TOF, ESI$^+$) *m/z* calculated for $C_{31}H_{42}O_5SiNa$, $[M+Na]^+$ 545.2699, found 545.2701.

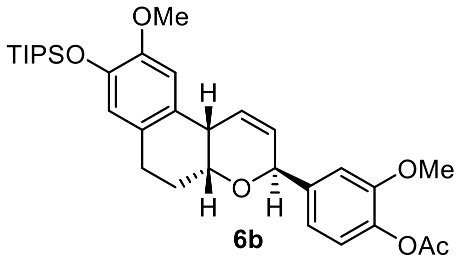


**Compound 6b** was obtained in 21% yield (17 mg, 31.4 μmol) from **5b** (85 mg, 149.4 μmol) as a yellowish oil, following the identical procedure for the synthesis of **6a** and **7a**, separated by flash column and then preparative TLC (hexane/dichloromethane = 1:2).

$[\alpha]_D^{20}$ = -73.2 (c 0.25, MeOH).

**$^1$H NMR** (400 MHz, $CDCl_3$) δ 7.08 – 6.98 (m, 3H), 6.71 (s, 1H), 6.59 (s, 1H), 6.18 (ddd, J = 10.3, 3.6, 2.1 Hz, 1H), 5.90 (dt, J = 10.2, 2.5 Hz, 1H), 5.17 (q, J = 2.4 Hz, 1H), 4.26 (ddd, J = 8.1, 4.8, 3.1 Hz, 1H), 3.87 (s, 3H), 3.79 (s, 3H), 3.48 (br s, 1H), 2.90 – 2.82 (m, 1H), 2.68 – 2.59 (m, 1H), 2.31 (s, 3H), 2.18 – 2.08 (m, 1H), 1.94 – 1.86 (m, 1H), 1.29 – 1.21 (m, 3H), 1.10 (d, J = 7.2 Hz, 18H).

**$^{13}$C{$^1$H} NMR** (100 MHz, $CDCl_3$) δ 169.2, 151.3, 149.6, 144.0, 140.3, 139.4, 130.0, 129.7, 128.5, 127.3, 122.7, 120.2, 120.1, 112.4, 112.0, 72.1, 69.2, 56.1, 56.0, 37.2, 26.3, 26.0, 20.8, 18.1 (6 x C), 13.1 (3 x C).

**HRMS** (TOF, ESI$^+$) *m/z* calculated for $C_{32}H_{44}O_6SiNa$, $[M+Na]^+$ 575.2805, found 575.2802.

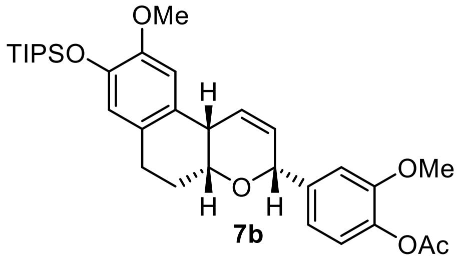


**Compound 7b** was obtained in 21% yield (17 mg, 31.4 μmol) from **5b** (85 mg, 149.4 μmol) as a yellowish oil, following the identical procedure for the synthesis of **6a** and **7a**, separated by flash column and then preparative TLC (hexane/dichloromethane = 1:2).

$[\alpha]_D^{20}$ = -83.0 (c 0.27, MeOH).

**$^1$H NMR** (400 MHz, $CDCl_3$) δ 6.87 (d, J = 7.8 Hz, 1H), 6.74 – 6.66 (m, 3H), 6.62 (s, 1H), 6.40 – 6.34 (m, 1H), 5.76 (d, J = 10.2 Hz, 1H), 5.21 (s, 1H), 4.24 (q, J = 3.5 Hz, 1H), 3.77 (s, 3H), 3.56 (s, 3H), 3.27 (br s, 1H), 3.07 – 2.96 (m, 1H), 2.54 – 2.45 (m, 1H), 2.26 (s, 3H), 2.17 – 2.09 (m, 1H), 1.95 – 1.85 (m, 1H), 1.29 – 1.20 (m, 3H), 1.09 (d, J = 7.4 Hz, 18H).

**$^{13}$C{$^1$H} NMR** (101 MHz, CDCl3) δ 169.2, 151.1, 149.3, 143.6, 140.9, 139.2, 130.5, 130.2, 128.5, 127.5, 122.5, 120.3, 119.1, 112.1, 111.1, 77.0, 70.7, 55.9, 55.7, 36.4, 28.6, 24.0, 20.8, 18.1 (6 x C), 13.1 (3 x C).

**HRMS** (TOF, ESI$^+$) *m/z* calculated for $C_{32}H_{44}O_6SiNa$, $[M+Na]^+$ 575.2805, found 575.2802.

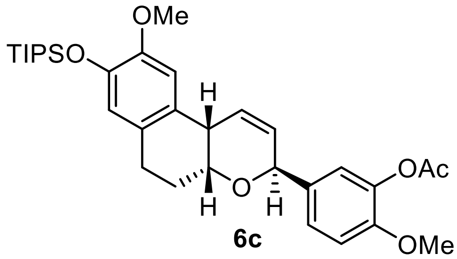


**Compound 6c** was obtained in 19% yield (13 mg, 23.4 μmol) from **5c** (70 mg, 123.0 μmol) as a yellowish oil, following the identical procedure for the synthesis of **6a** and **7a**, separated by flash column and then preparative TLC (hexane/dichloromethane = 1:2).

$[\alpha]_D^{20}$ = -189.2 (c 0.25, MeOH).

**$^1$H NMR** (400 MHz, $CDCl_3$) δ 7.27 (dd, J = 8.5 Hz, 1.8 Hz, 1H), 7.13 (s, 1H), 6.96 (d, J = 8.5 Hz, 1H), 6.70 (s, 1H), 6.58 (s, 1H), 6.20 (ddd, J = 10.3, 3.9, 2.1 Hz, 1H), 5.89 (dt, J = 10.3, 2.5 Hz, 1H), 5.14 (d, J = 2.4 Hz, 1H), 4.20 (m, 1H), 3.84 (s, 3H), 3.79 (s, 3H), 3.42 (br s, 1H), 2.90 – 2.80 (m, 1H), 2.66 – 2.55 (m, 1H), 2.32 (s, 3H), 2.14 – 2.05 (m, 1H), 1.91 – 1.82 (m, 1H), 1.25 (m, 3H), 1.09 (d, J = 7.3 Hz, 18H).

**$^{13}$C{$^1$H} NMR** (100 MHz, $CDCl_3$) δ 169.1, 150.8, 149.6, 144.0, 139.8, 134.1, 130.0, 129.7, 128.5,

127.3, 126.4, 122.6, 120.3, 112.4, 112.3, 71.7, 68.6, 56.2, 56.0, 37.1, 26.2, 26.1, 20.8, 18.1 (6 x C), 13.1 (3 x C).

**HRMS** (TOF, ESI$^+$) *m/z* calculated for $C_{32}H_{44}O_6SiNa$, [M+Na]$^+$ 575.2805, found 575.2806.

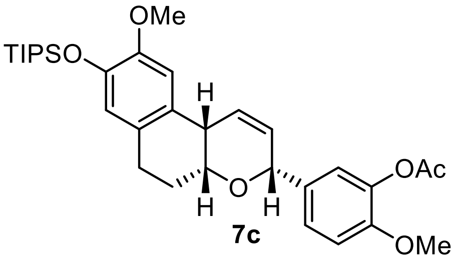


7c

**Compound 7c** was obtained in 20% yield (14 mg, 24.5 μmol) from **5c** (70 mg, 123.0 μmol) as a yellowish oil, following the identical procedure for the synthesis of **6a** and **7a**, separated by flash column and then preparative TLC (hexane/dichloromethane = 1:2).

$[\alpha]_D^{20}$ = -60.7 (c 0.27, MeOH).

**$^1$H NMR** (400 MHz, $CDCl_3$) δ 6.97 (d, J = 8.1 Hz, 1H), 6.81 (d, J = 7.7 Hz, 1H), 6.80 (s, 1H), 6.72 (s, 1H), 6.62 (s, 1H), 6.34 (m, 1H), 5.76 (d, J = 10.2 Hz, 1H), 5.15 (s, 1H), 4.21 (q, J = 3.6 Hz, 1H), 3.78 (s, 3H), 3.77 (s, 3H), 3.24 (br s, 1H), 2.98 (ddd, J = 16.8, 12.4, 5.0 Hz, 1H), 2.45 (dt, J = 16.5, 4.5 Hz, 1H), 2.26 (s, 3H), 2.11 (m, 1H), 1.89 (m, 1H), 1.29 – 1.23 (m, 3H), 1.10 (d, J = 7.3 Hz, 18H).

**$^{13}$C{$^1$H} NMR** (100 MHz, $CDCl_3$) δ 169.1, 150.7, 149.3, 143.5, 139.7, 134.6, 130.4, 130.1, 128.6, 127.7, 125.5, 121.7, 120.5, 112.4, 112.0, 76.5, 71.0, 56.1, 55.9, 36.4, 28.6, 24.0, 20.8, 18.1 (6 x C), 13.1 (3 x C).

**HRMS** (TOF, ESI$^+$) *m/z* calculated for $C_{32}H_{44}O_6SiNa$, [M+Na]$^+$ 575.2805, found 575.2805.

**Deacetylation with $K_2CO_3$/MeOH and Desilylation with TBAF**

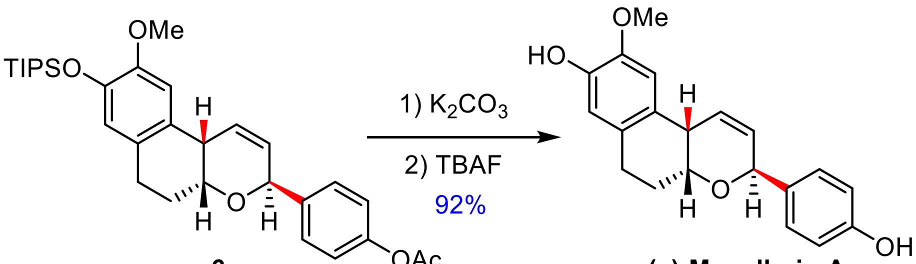


6a (–)-Musellarin A

To a stirred solution of Friedel-Crafts cyclization product **6a** (40 mg, 76.5 μmol) in methanol (5 mL) was added $K_2CO_3$ (23.2 mg, 0.168 mmol, 2.2 equiv) at rt. The reaction mixture was stirred for 3 h and then the solvent was removed under reduced pressure. The resulting residue was diluted with $CH_2Cl_2$ and quenched by addition of saturated aqueous $NH_4Cl$ (10 mL) and acidified by 1 M HCl. The organic phase was collected and the aqueous phase was extracted with extracted with $CH_2Cl_2$ (3 × 5 mL). The combined organic fractions were washed with brine, dried over $Na_2SO_4$, filtered and concentrated under reduced pressure. The resulting crude products were used without purification for next step, or separated by flash column chromatography (hexane/dichloromethane = 1:2, if 2 diastereomers were not separated in the last step).

To a THF solution (5 mL) of the crude product from deacetylation was added TBAF (1.0 M in THF, 91.8 μL, 91.8 μmol, 1.2 equiv) at rt. The reaction mixture was stirred for 15 min and then the solvent was removed under reduced pressure. The resulting residue was purified by flash column chromatography on silica gel (hexane/EtOAc = 4:1 to 3:1) to afford **(–)-musellarin A** in 92% yield (22.7 mg, 70.0 μmol) as a colorless oil.

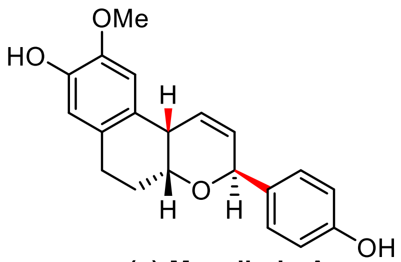


**(–)-Musellarin A**

$[\alpha]_D^{20}$ = -243.8 (c 0.81, MeOH).

**$^1$H NMR** (400 MHz, $CD_3OD$) δ 7.24 (d, J = 8.5 Hz, 2H), 6.83 (s, 1H), 6.78 (d, J = 8.5 Hz, 2H), 6.52 (s, 1H), 6.26 (ddd, J = 10.4, 4.0, 2.1 Hz, 1H), 5.86 (dt, J = 10.3, 2.5 Hz, 1H), 5.08 (d, J = 2.4 Hz, 1H), 4.18 (ddd, J = 8.0, 4.9, 3.1 Hz, 1H), 3.84 (s, 3H), 3.42 (s, 1H), 2.84 (ddd, J = 16.1, 7.7, 5.1 Hz, 1H), 2.59 (ddd, J = 16.0, 7.1, 5.2 Hz, 1H), 2.10 – 2.01 (m, 1H), 1.89 – 1.80 (m, 1H).

**$^{13}$C{$^1$H} NMR** (100 MHz, $CD_3OD$) δ 158.4, 147.8, 145.6, 133.0, 130.5, 130.3, 129.9, 129.7, 128.4, 116.0, 115.9, 112.7, 73.7, 69.7, 56.5, 38.2, 27.2, 26.9.

**HRMS** (TOF, ESI$^-$) *m/z* calculated for $C_{20}H_{19}O_4$, $[M-H]^-$ 323.1283, found 323.1286.

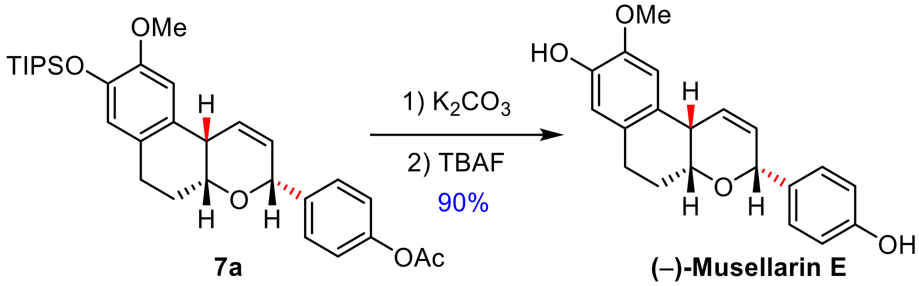


**7a** **(–)-Musellarin E**

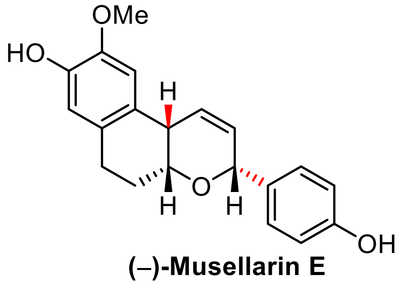


**(–)-Musellarin E**

**(–)-Musellarin E** was obtained in 90% yield (20.6 mg, 63.6 μmol) from **7a** (37 mg, 70.7 μmol) as a colorless oil, following the identical procedure for the synthesis of **(–)-musellarin A**.

$[\alpha]_D^{20}$ = -87.6 (c 0.84, MeOH).

**$^1$H NMR** (400 MHz, $CD_3OD$) δ 6.96 (d, J = 8.5 Hz, 2H), 6.85 (s, 1H), 6.65 (d, J = 8.5 Hz, 2H), 6.55 (s, 1H), 6.42 (ddd, J = 10.2, 5.9, 2.2 Hz, 1H), 5.74 (dt, J = 10.1, 1.6 Hz, 1H), 5.11 (br s), 4.22 (q, J = 3.5 Hz, 1H), 3.83 (s, 3H), 3.24 (br s, 1H), 2.93 (ddd, J = 16.8, 12.4, 5.1 Hz, 1H), 2.43 (ddd, J = 15.9, 5.6, 3.1 Hz, 1H), 2.05 (m, 1H), 1.87 (m, 1H).

**$^{13}$C{$^1$H} NMR** (100 MHz, $CD_3OD$) δ 158.1, 147.5, 145.2, 133.9, 131.5, 129.9, 129.8, 129.5 (2 x C), 128.5, 116.1, 116.0 (2 x C), 112.4, 78.3, 72.4, 56.6, 37.5, 29.5, 24.8.

**HRMS** (TOF, ESI$^-$) *m/z* calculated for $C_{20}H_{19}O_4$, $[M-H]^-$ 323.1283, found 323.1285.

**Spectroscopic Data Comparison of Our Synthetic Musellarin E with Those Reported for Natural Musellarin E**

**Table S-1. NMR Comparison**

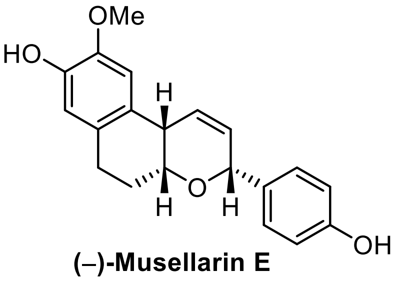


(–)-Musellarin E

| NO. | $^{13}C\{^1H\}$ NMR ($CD_3OD$) | | $^1H$ NMR ($CD_3OD$) | |
|---|---|---|---|---|
| | Natural (-)-Musellarin E $^{13}C\{^1H\}$ NMR (100 MHz) | Synthetic (-)-Musellarin E $^{13}C\{^1H\}$ NMR (100 MHz) | Natural (-)-Musellarin E $^1H$ NMR (400 MHz) | Synthetic (-)-Musellarin E $^1H$ NMR (400 MHz) |
| 1 | 128.5, CH | 128.5, CH | 6.42, ddd (10.0, 6.0, 1.8) | 6.42 (ddd, 10.2, 5.9, 2.2) |
| 2 | 131.5, CH | 131.5, CH | 5.74, dt (10.0, 1.8) | 5.74 (dt, 10.1, 1.6) |
| 3 | 78.4, CH | 78.3, CH | 5.11, br s | 5.11, br s |
| 4a | 72.5, CH | 72.4, CH | 4.22, dd (6.8, 3.8) | 4.22 (q, 3.5) |
| 5 | 29.6, CH2 | 29.5, CH2 | 1.87, m (α); 2.04, m (β) | 1.87, m (α); 2.05, m (β) |
| 6 | 24.8, CH2 | 24.8, CH2 | 2.43, ddd (16.0, 5.5, 3.0, α); 2.93, ddd (16.0, 12.5, 4.5, β) | 2.43, ddd (15.9, 5.6, 3.1, α); 2.93, ddd (16.8, 12.4, 5.1, β) |
| 6a | 129.9, C | 129.8, C | | |
| 7 | 116.1, CH | 116.1, CH | 6.54, s | 6.55, s |
| 8 | 145.3, C | 145.2, C | | |
| 9 | 147.6, C | 147.5, C | | |
| 10 | 112.5, CH | 112.4, CH | 6.86, s | 6.85, s |
| 10a | 129.9, C | 129.9, C | | |
| 10b | 37.5, CH | 37.5, CH | 3.25, br s | 3.24, br s |
| 1’ | 134.0, C | 133.9, C | | |
| 2’ | 129.5, CH | 129.5, CH | 6.96, d (8.5) | 6.96, d (8.5) |
| 3’ | 116.0, CH | 116.0, CH | 6.64, d (8.5) | 6.65, d (8.5) |
| 4’ | 158.1, C | 158.1, C | | |
| 5’ | 116.0, CH | 116.0, CH | 6.64, d (8.5) | 6.65, d (8.5) |
| 6’ | 129.5, CH | 129.5, CH | 6.96, d (8.5) | 6.96, d (8.5) |
| 9-$OCH_3$ | 56.6, CH3 | 56.6, CH3 | 3.83, s | 3.83, s |

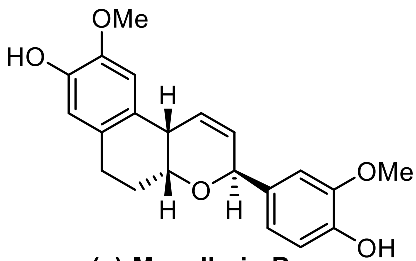

(–)-Musellarin B

**(–)-Musellarin B** was obtained in 89% yield (9.1 mg, 28.1 μmol) from **6b** (17 mg, 31.4 μmol) as a colorless oil, following the identical procedure for the synthesis of **(–)-musellarin A**.

$[\alpha]_D^{20}$ = -171.4 (c 0.21, MeOH).

**$^1$H NMR** (400 MHz, acetone-d6) δ 7.58 (s, 1H), 7.34 (s, 1H), 7.02 (d, J = 1.6 Hz, 1H), 6.90 (s, 1H), 6.87 (dd, J = 8.0, 1.6 Hz, 1H), 6.81 (d, J = 8.4 Hz, 1H), 6.55 (s, 1H), 6.29 (ddd, J = 10.4, 4.0, 2.0 Hz, 1H), 5.91 (dt, J = 10.4, 2.4 Hz, 1H), 5.06 (q, J = 2.4 Hz, 1H), 4.15 (ddd, J = 7.5, 4.8, 2.9 Hz, 1H), 3.85 (s, 3H), 3.83 (s, 3H), 3.37 br (s, 1H), 2.90 – 2.84 (m, 1H), 2.56 (dt, J = 16.0, 6.0 Hz, 1H), 2.04 – 1.95 (m, 1H), 1.80 (tdd, J = 8.4, 5.4, 3.2 Hz, 1H).

**$^{13}$C{$^1$H} NMR** (100 MHz, acetone-d6) δ 148.2, 147.13, 147.05, 145.6, 133.8, 129.9, 129.5, 129.4, 128.5, 121.4, 115.5, 115.4, 112.5, 112.3, 73.1, 68.2, 56.4, 56.2, 37.6, 27.2, 26.3.

**HRMS** (TOF, ESI$^-$) *m/z* calculated for $C_{21}H_{21}O_5$, [M-H]$^-$ 353.1389, found 353.1393.

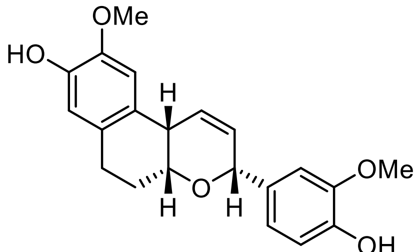

(–)-Musellarin D

**(–)-Musellarin D** was obtained in 88% yield (9.0 mg, 27.8 μmol) from **7b** (17 mg, 31.4 μmol) as a colorless oil, following the identical procedure for the synthesis of **(–)-musellarin A**.

$[\alpha]_D^{20}$ = -44.5 (c 0.20, MeOH).

**$^1$H NMR** (400 MHz, $CD_3OD$) δ 6.86 (s, 1H), 6.64 (d, J = 8.0 Hz, 1H), 6.63 (dd, J = 8.1, 1.8 Hz, 1H), 6.56 (s, 1H), 6.52 (d, J = 1.6 Hz, 1H), 6.44 (ddd, J = 10.1, 6.3, 2.0 Hz, 1H), 5.73 (dt, J = 10.1, 1.4 Hz, 1H), 5.12 (q, J = 2.2 Hz, 1H), 4.22 (q, J = 3.4 Hz, 1H), 3.82 (s, 3H), 3.51 (s, 3H), 3.26 (br s, 1H), 2.96 (ddd, J = 17.1, 12.4, 5.5 Hz, 1H), 2.48 (ddd, J = 16.1, 5.8, 2.8 Hz, 1H), 2.06 (m, 1H), 1.89 (m, 1H).

**$^{13}$C{$^1$H} NMR** (100 MHz, $CD_3OD$) δ 148.9, 147.6, 147.1, 145.4, 134.9, 131.6, 130.3, 129.8, 128.3, 120.7, 116.1, 115.5, 112.7, 111.4, 78.4, 72.0, 56.6, 55.9, 37.6, 29.3, 24.8.

**HRMS** (TOF, ESI$^-$) *m/z* calculated for $C_{21}H_{21}O_5$, [M-H]$^-$ 353.1389, found 353.1394.

**<u>Spectroscopic Data Comparison of Our Synthetic Musellarin D with Those Reported for Natural Musellarin D</u>**

**Table S-2. NMR Comparison**

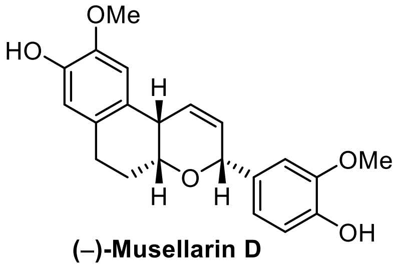


**(–)-Musellarin D**

| NO. | $^{13}C\{^1H\}$ NMR ($CD_3OD$) | | $^1H$ NMR ($CD_3OD$) | |
|---|---|---|---|---|
| | Natural (-)-Musellarin D $^{13}C\{^1H\}$ NMR (100 MHz) | Synthetic (-)-Musellarin D $^{13}C\{^1H\}$ NMR (100 MHz) | Natural (-)-Musellarin D $^1H$ NMR (400 MHz) | Synthetic (-)-Musellarin D $^1H$ NMR (400 MHz) |
| 1 | 128.3, CH | 128.3, CH | 6.45, ddd (10.0, 6.4, 1.8) | 6.44 (ddd, 10.1, 6.3, 2.0) |
| 2 | 131.6, CH | 131.6, CH | 5.73, br d (10.0) | 5.73 (dt, 10.1, 1.4) |
| 3 | 78.4, CH | 78.4, CH | 5.13, d (1.9) | 5.12 (q, 2.2) |
| 4a | 72.0, CH | 72.0, CH | 4.23, dd (5.6, 3.2) | 4.22 (q, 3.4) |
| 5 | 29.3, CH2 | 29.3, CH2 | 1.90, m (α); 2.07, m (β) | 1.89, m (α); 2.06, m (β) |
| 6 | 24.8, CH2 | 24.8, CH2 | 2.49, ddd (16.6, 5.6, 2.4, α); 2.96, ddd (16.6, 12.8, 4.8, β) | 2.48 (ddd, 16.1, 5.8, 2.8); 2.96 (ddd, 17.1, 12.4, 5.5) |
| 6a | 129.7, C | 129.8, C | | |
| 7 | 116.0, CH | 116.1, CH | 6.56, s | 6.56, s |
| 8 | 145.4, C | 145.4, C | | |
| 9 | 147.6, C | 147.6, C | | |
| 10 | 112.6, CH | 112.7, CH | 6.87, s | 6.86, s |
| 10a | 130.3, C | 130.3, C | | |
| 10b | 37.6, CH | 37.6, CH | 3.27, br s | 3.26, br s |
| 1’ | 134.8, C | 134.9, C | | |
| 2’ | 111.4, CH | 111.4, CH | 6.52, d (1.2) | 6.52, d (1.6) |
| 3’ | 148.9, C | 148.9, C | | |
| 4’ | 147.1, C | 147.1, C | | |
| 5’ | 115.5, CH | 115.5, CH | 6.64, overlap | 6.64 (d, 8.0) |
| 6’ | 120.7, CH | 120.7, CH | 6.63, overlap | 6.63 (dd, 8.1, 1.8) |
| 9-$OCH_3$ | 56.5, CH3 | 56.6, CH3 | 3.82, s | 3.82, s |
| 3’-$OCH_3$ | 55.9, CH3 | 55.9, CH3 | 3.50, s | 3.51, s |

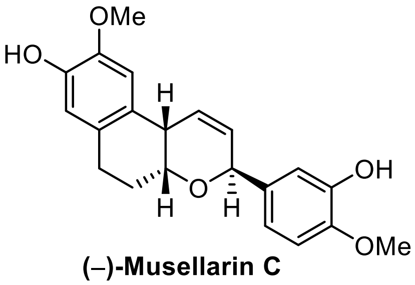


**(–)-Musellarin C**

<u>**(–)-Musellarin C**</u> was obtained in 85% yield (7.0 mg, 19.8 µmol) from **6c** (13 mg, 23.4 µmol) as a colorless oil, following the identical procedure for the synthesis of **(–)-musellarin A**.

$[\alpha]_D^{20}$ = -244.5 (c 0.20, MeOH).

**$^1$H NMR** (400 MHz, $CD_3OD$) δ 6.90 (d, J = 8.4 Hz, 1H), 6.90 (s, 1H), 6.85 (dd, J = 8.0, 2.0 Hz, 1H), 6.83 (s, 1H), 6.52 (s, 1H), 6.26 (ddd, J = 10.4, 4.0, 2.0 Hz, 1H), 5.87 (dt, J = 10.4, 2.4 Hz, 1H), 5.05 (q, J = 2.4 Hz, 1H), 4.18 (ddd, J = 7.9, 4.9, 3.1 Hz, 1H), 3.85 (s, 3H), 3.84 (s, 3H), 3.41 (br s, 1H), 2.84 (ddd, J = 15.8, 7.6, 5.4 Hz, 1H), 2.58 (ddd, J = 16.1, 7.3, 5.4 Hz, 1H), 2.10 – 2.01 (m, 1H), 1.89 – 1.80 (m, 1H).

**$^{13}$C{$^1$H} NMR** (100 MHz, $CD_3OD$) δ 148.8, 147.8, 147.5, 145.6, 135.1, 130.2, 129.8, 129.7, 128.4, 120.5, 116.1, 115.9, 112.7, 112.4, 73.7, 69.7, 56.5, 56.4, 38.1, 27.3, 26.8.

**HRMS** (TOF, ESI$^-$) *m/z* calculated for $C_{21}H_{21}O_5$, $[M\text{-}H]^-$ 353.1389, found 353.1380.

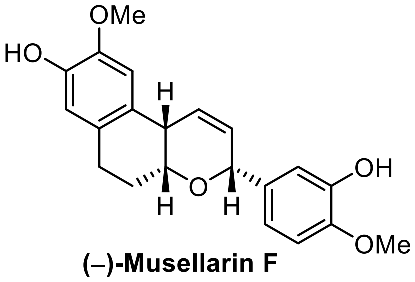


**(–)-Musellarin F**

<u>**(–)-Musellarin F**</u> was obtained in 88% yield (7.6 mg, 21.5 µmol) from **7c** (14 mg, 24.5 µmol) as a colorless oil, following the identical procedure for the synthesis of **(–)-musellarin A**.

$[\alpha]_D^{20}$ = -90.5 (c 0.20, MeOH).

**$^1$H NMR** (400 MHz, $CD_3OD$) δ 6.86 (s, 1H), 6.79 (d, J = 8.2 Hz, 1H), 6.64 (dd, J = 8.2, 2.1 Hz, 1H), 6.61 (d, J = 2.0 Hz, 1H), 6.55 (s, 1H), 6.41 (ddd, J = 10.1, 5.9, 2.2 Hz, 1H), 5.73 (dt, J = 10.1, 1.7 Hz, 1H), 5.08 (br s, 1H), 4.22 (q, J = 3.6 Hz, 1H), 3.83 (s, 3H), 3.79 (s, 3H), 3.25 (br s, 1H), 2.95 (ddd, J = 16.8, 12.5, 5.1 Hz, 1H), 2.44 (ddd, J = 15.9, 5.5, 3.0 Hz, 1H), 2.10 – 2.03 (m, 1H), 1.92 – 1.83 (m, 1H).

**$^{13}$C{$^1$H} NMR** (100 MHz, $CD_3OD$) δ 148.7, 147.6, 147.4, 145.3, 136.0, 131.4, 129.9, 129.8, 128.5, 119.7, 116.1, 115.2, 112.4, 112.4, 78.4, 72.5, 56.6, 56.4, 37.5, 29.5, 24.8.

**HRMS** (TOF, ESI$^-$) *m/z* calculated for $C_{21}H_{21}O_5$, $[M\text{-}H]^-$ 353.1389, found 353.1380.

**Hydrogenation of Musellarins**

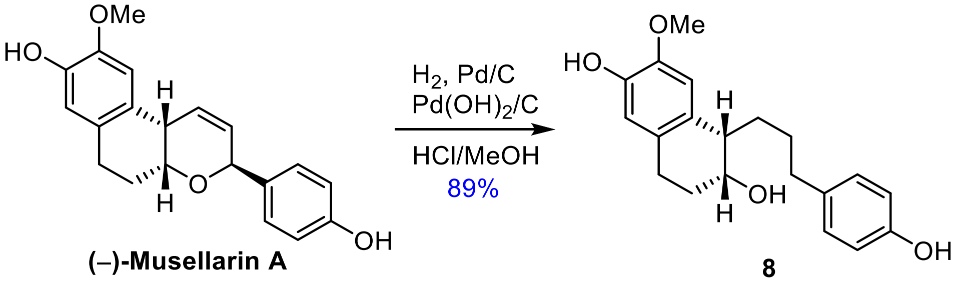


**(–)-Musellarin A** **8**

To a stirred solution of **(–)-musellarin A** (4.6 mg, 14.1 µmol) in MeOH (0.5 mL) was added Pd/C (5% Pd loaded, 3.0 mg, 1.41 µmol, 0.1 equiv.) at rt. The reaction mixture under hydrogen gas atmosphere (balloon, 1 atm) was allowed to stir for 4 h. After TLC (PMA stained) showed that all SM was consumed, hydrochloric acid solution (HCl, 8.0 M in methanol, 8.8 µL, 70.5 mmol, 5.0 equiv.) and $Pd(OH)_2/C$ (20% $Pd(OH)_2$ loaded, 1.0 mg, 1.41 µmol, 0.1 equiv.) were added at rt. The reaction mixture was allowed to stir for another 12 h. Then the reaction mixture was filtered through

filtration membrane and concentrated under reduced pressure. The resulting residue was purified by flash column chromatography on silica gel (hexane/EtOAc = 3:1) to afford the desired product **8** in 89% yield (4.1 mg, 12.5 μmol) from **(–)-musellarin A** or **E** as a white solid.

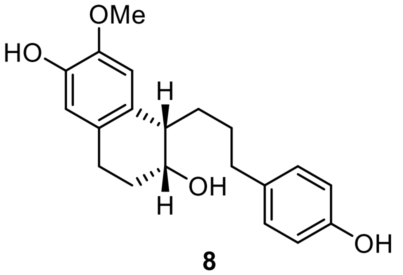


**8**

$[\alpha]_D^{20}$ = +22.0 (c 0.41, MeOH).

**¹H NMR** (400 MHz, Methanol-d4) δ 6.95 (d, J = 8.5 Hz, 2H), 6.64 (d, J = 8.5 Hz, 2H), 6.55 (s, 1H), 6.48 (s, 1H), 4.03 (dt, J = 9.9, 4.1 Hz, 1H), 3.74 (s, 3H), 2.82 (ddd, J = 16.9, 6.8, 4.9 Hz, 1H), 2.71 – 2.45 (m, 4H), 1.93 – 1.67 (m, 5H), 1.49 – 1.38 (m, 1H).

**¹³C{¹H} NMR** (100 MHz, MeOD) δ 158.1, 147.4, 147.3, 133.7, 130.9, 130.2 (2 x C), 128.8, 116.7 (2 x C), 116.6, 113.5, 70.4, 56.5, 45.1, 36.5, 31.7, 31.1, 28.2, 27.5.

**HRMS** (TOF, ESI⁻) *m/z* calculated for $C_{20}H_{23}O_4$, $[M-H]^-$ 327.1596, found 327.1581.

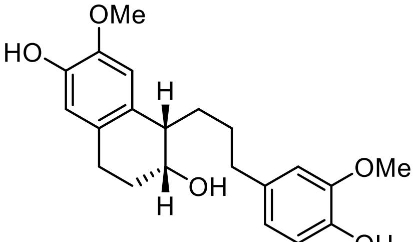


**8-OMe**

**<u>Compound 8-OMe</u>** was obtained in 85% yield (4.3 mg, 12.0 μmol) from **(–)-musellarin B** or **D** (4.6 mg, 14.1 μmol) as a white solid.

$[\alpha]_D^{20}$ = +27.7 (c 0.26, MeOH).

**¹H NMR** (400 MHz, Methanol-d4) δ 6.73 (d, J = 1.9 Hz, 1H), 6.68 (d, J = 8.0 Hz, 1H), 6.61 (dd, J = 8.0, 1.9 Hz, 1H), 6.58 (s, 1H), 6.50 (s, 1H), 4.04 (dt, J = 9.5, 4.3 Hz, 1H), 3.80 (s, 3H), 3.76 (s, 3H), 2.83 (ddd, J = 16.8, 6.6, 4.7 Hz, 1H), 2.73 – 2.49 (m, 4H), 1.94 – 1.71 (m, 5H), 1.50 – 1.40 (m, 1H).

**¹³C{¹H} NMR** (100 MHz, MeOD) δ 148.8, 146.9, 145.7, 145.4, 135.6, 132.0, 128.9, 121.8, 116.1, 116.0, 113.6, 113.1, 70.3, 56.5, 56.3, 45.1, 36.8, 31.4, 31.0, 28.1, 27.5.

**HRMS** (TOF, ESI⁻) *m/z* calculated for $C_{21}H_{25}O_5$, $[M-H]^-$ 357.1702, found 357.1702.

**Epimerization of Musellarins**

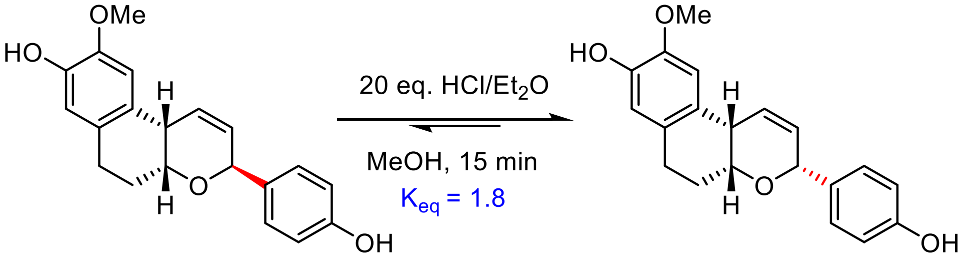


**(–)-Musellarin A** **(–)-Musellarin E**

To a stirred solution of **(–)-musellarin A** or **E** (4.6 mg, 14.1 μmol) in MeOH (0.5 mL) was added hydrochloric acid solution (HCl, 1.0 M in ether, 0.28 mL, 0.28 mmol, 20 equiv.) dropwise at rt. The reaction mixture was stirred at rt for 15 min. The reaction was quenched by addition of saturated aqueous $NaHCO_3$ (1 mL) and extracted with $CH_2Cl_2$ (3 × 1 mL). The combined organic fractions were washed with brine, dried over $Na_2SO_4$, filtered and concentrated under reduced pressure. The resulting residue was purified by flash column chromatography on silica gel (hexane/EtOAc = 4:1 to 3:1) to afford the inseparable epimerized mixture **(–)-musellarin A** and **E** (ratio 1:1.8).

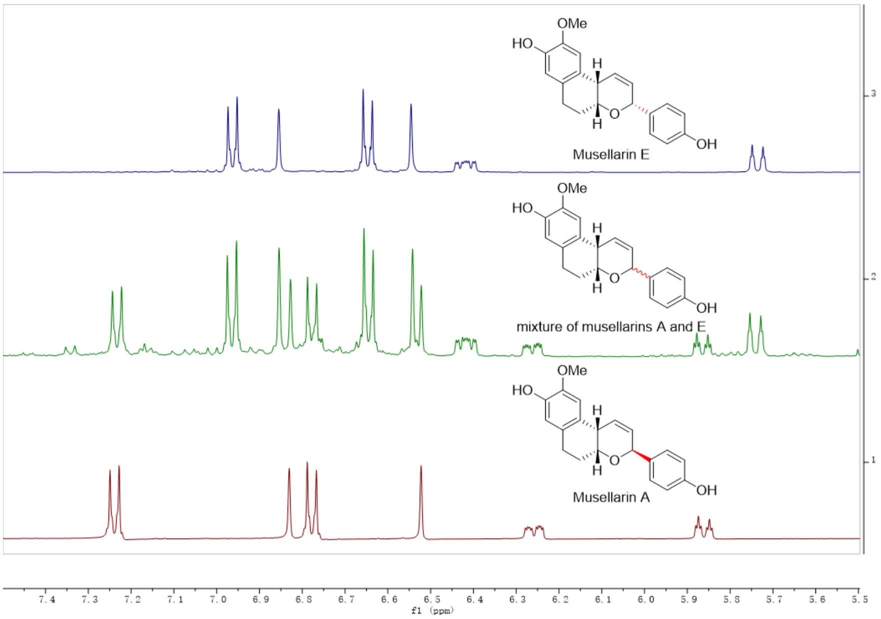


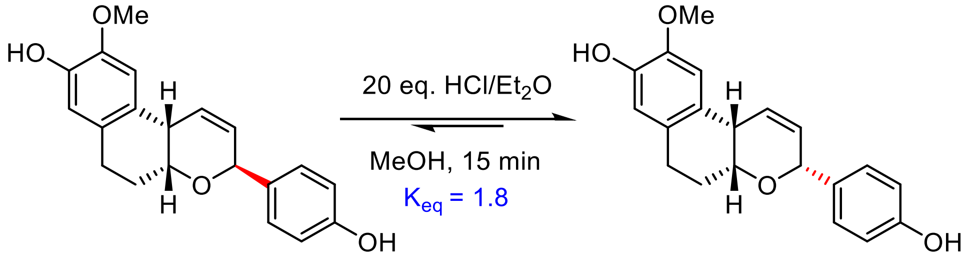


**(–)-Musellarin A** **(–)-Musellarin E**

The inseparable epimerized mixture **(–)-musellarin B** and **D** (ratio 1:2.2) was obtained, following the identical procedure of epimerization of **(–)-musellarin A** or **E**.

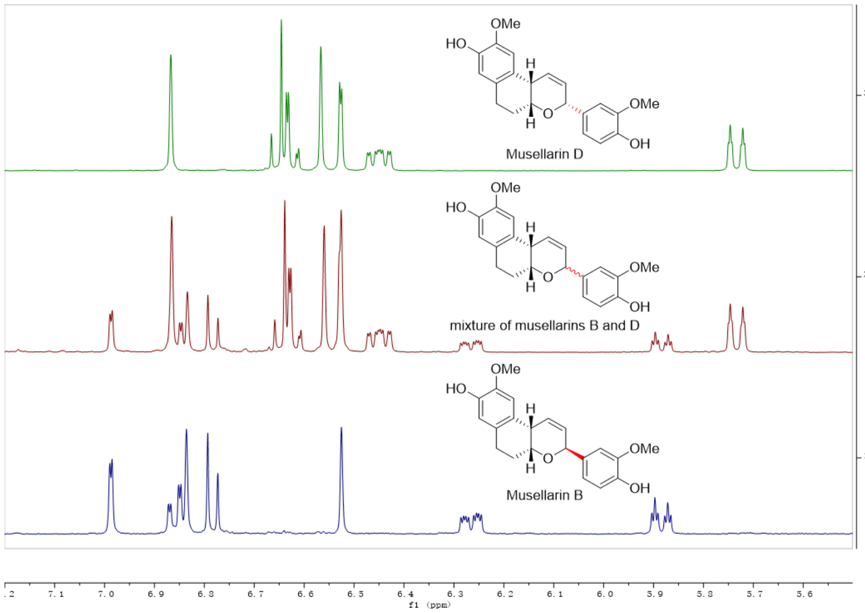

## NOE spectra comparison of Musellarins A and E

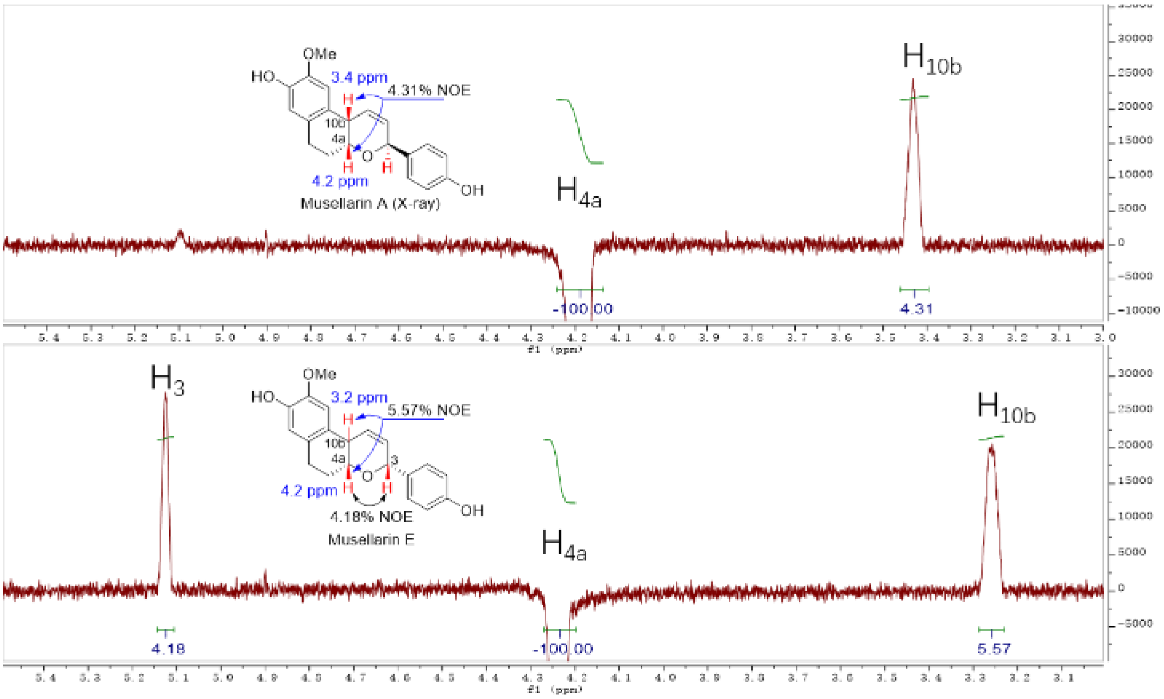


## NOE spectra comparison of Musellarins B and D

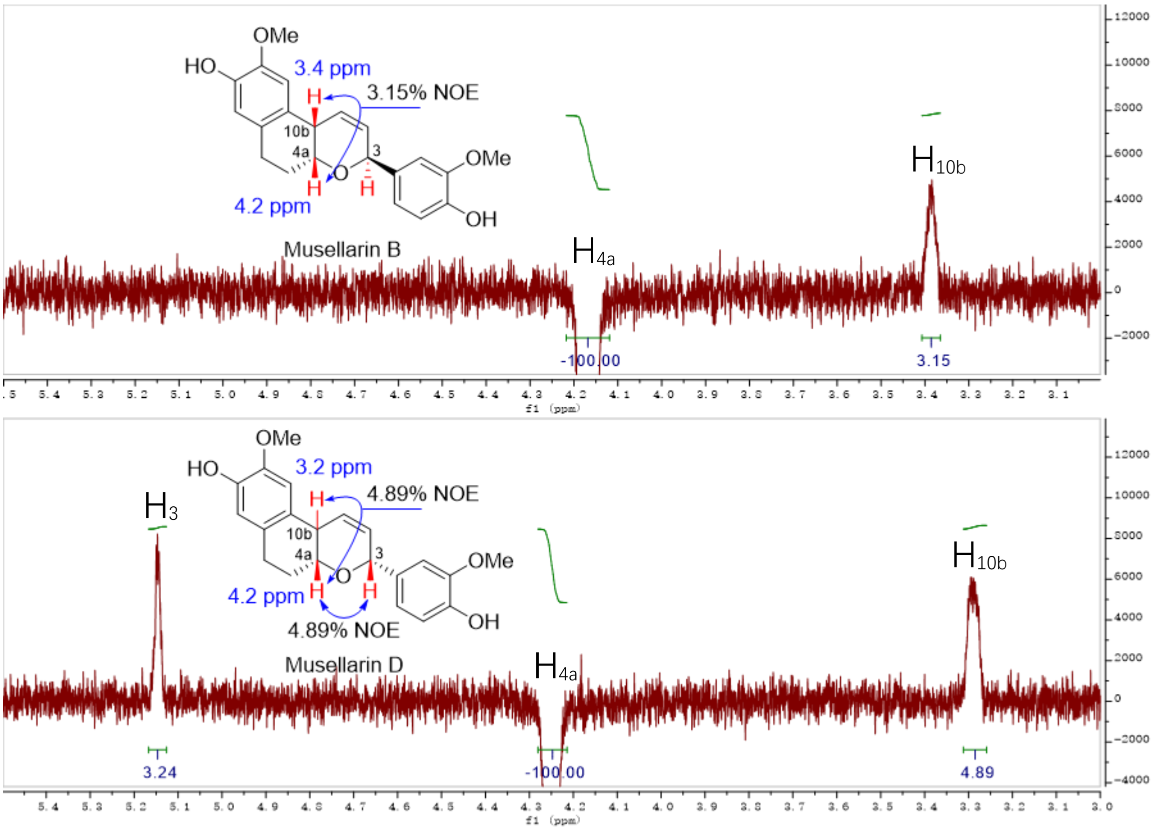

**Synthesis of dibenzofuran analogues**

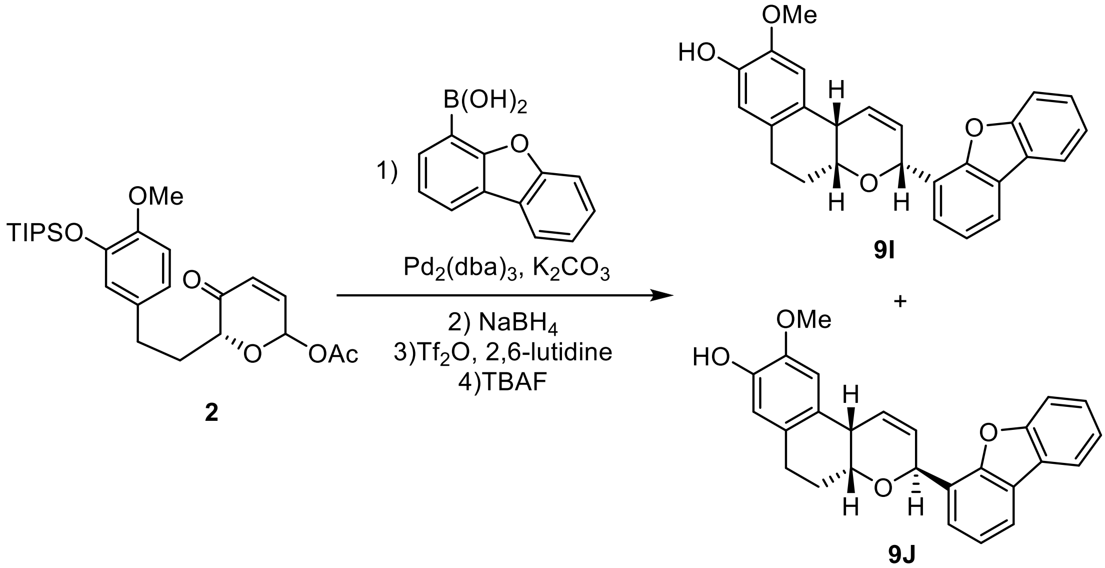


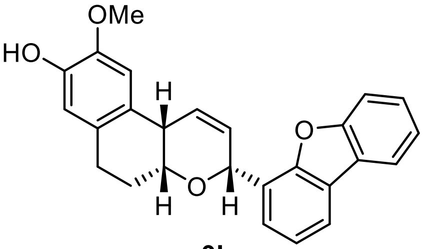

9I

**Compound 9I** was obtained in 7% yield (5.6 mg, 14.1 μmol) over 4 steps from **2** (100 mg, 0.216 mmol) as a white solid, following the identical procedure of **Arylation of Achmatowicz Rearrangement Product with Arylboronic Acids, Ketone reduction and Friedel-Crafts Cyclization, Desilylation with TBAF**.

$[\alpha]_D^{20}$ = -15.0 (c 1, MeOH).

**$^1$H NMR** (400 MHz, $CDCl_3$) δ 7.92 (d, J = 7.7 Hz, 1H), 7.80 (d, J = 7.6 Hz, 1H), 7.50 (d, J = 8.3 Hz, 1H), 7.43 (t, J = 7.7 Hz, 1H), 7.33 (d, J = 7.5 Hz, 1H), 7.30 (d, J = 7.0 Hz, 1H), 7.24 – 7.19 (m, 1H), 6.78 (s, 1H), 6.74 (s, 1H), 6.44 – 6.36 (m, 1H), 6.02 (d, J = 10.0 Hz, 1H), 5.97 (s, 1H), 5.48 (s, 1H), 4.42 – 4.37 (m, 1H), 3.86 (s, 3H), 3.35 (br s, 1H), 3.20 – 3.08 (m, 1H), 2.60 – 2.51 (m, 1H), 2.25 – 2.18 (m, 1H), 2.10 – 1.89 (m, 1H).

**$^{13}$C{$^1$H} NMR** (100 MHz, $CDCl_3$) δ 156.2, 153.3, 145.3, 143.6, 129.6, 129.5, 128.9, 127.6, 127.2, 125.7, 125.1, 124.5, 124.2, 123.2, 122.8, 120.8, 119.7, 114.5, 111.8, 110.2, 71.5, 71.1, 56.2, 36.4, 28.8, 24.1.

**HRMS** (TOF, ESI$^+$) *m/z* calculated for $C_{26}H_{22}O_4Na$, $[M+Na]^+$ 421.1416, found 421.1414.

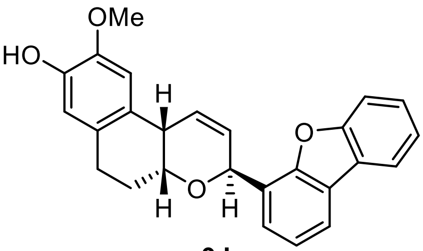

9J

**Compound 9J** was obtained in 8% yield (7.1 mg, 17.8 μmol) over 4 steps from **2** (100 mg, 0.216 mmol) as a white solid, following the identical procedure of **Arylation of Achmatowicz Rearrangement Product with Arylboronic Acids, Ketone reduction and Friedel-Crafts Cyclization, Desilylation with TBAF**.

$[\alpha]_D^{20}$ = -151.4 (c 1, MeOH).

**$^1$H NMR** (400 MHz, $CDCl_3$) δ 7.96 (dd, J = 7.9, 1.2 Hz, 1H), 7.91 (dd, J = 7.6, 1.3 Hz, 1H), 7.61 (d, J = 8.3 Hz, 1H), 7.60 – 7.57 (m, 1H), 7.49 – 7.43 (m, 1H), 7.39 – 7.32 (m, 2H), 6.77 (s, 1H), 6.70 (s, 1H), 6.25 (ddd, J = 10.2, 3.8, 2.2 Hz, 1H), 6.12 (dt, J = 10.3, 2.4 Hz, 1H), 5.91 (q, J = 2.4 Hz, 1H), 5.50 (br s, 1H), 4.42 (ddd, J = 8.4, 5.1, 3.2 Hz, 1H), 3.91 (s, 3H), 3.58 (br s, 1H), 2.98 (ddd, J = 16.3, 7.1, 5.4 Hz, 1H), 2.70 (ddd, J = 16.2, 8.1, 5.4 Hz, 1H), 2.24 (dtd, J = 13.2, 8.2, 5.1 Hz, 1H),

1.99 (dddd, J = 12.7, 7.2, 5.5, 3.2 Hz, 1H).
**$^{13}C\{^1H\}$ NMR** (100 MHz, $CDCl_3$) δ 156.3, 154.0, 145.5, 144.0, 129.3, 129.3, 129.2, 127.3, 127.0, 125.7, 125.2, 124.6, 124.4, 122.9, 122.8, 120.8, 120.2, 114.4, 112.0, 110.7, 69.4, 67.7, 56.2, 37.2, 26.5, 26.0.
**HRMS** (TOF, $ESI^+$) *m/z* calculated for $C_{26}H_{22}O_4Na$, $[M+Na]^+$ 421.1416, found 421.1414.

**Anti-inflammatory tests of Musellarins A-F and analogues**

**Materials and Methods**

Cell Culture

RAW264.7 macrophages were cultured in DMEM supplemented with 10% fetal bovine serum and 1% penicillin–streptomycin (Procell, China) at 37 °C in a humidified incubator with 5% $CO_2$. For inflammatory stimulation, cells were treated with lipopolysaccharide (LPS, 1 μg/mL, Sigma) for 4 h (for RT-qPCR) or 24 h (for NO and ELISA assays), with or without the indicated compounds.

Cytotoxicity Assay

Cell viability was assessed using the CCK-8 assay. RAW264.7 cells were seeded into 96-well plates at $2 \times 10^4$ cells/well and incubated overnight. After exposure to compounds at the indicated concentrations for 24 h, the medium was replaced with DMEM containing 10% CCK-8 solution (Abbkine, USA) and incubated for 30 min at 37 °C. Absorbance at 450 nm was measured using a Infinite M Plex microplate reader (Tecan, Switzerland).

Quantitative Real-Time PCR (RT-qPCR)

Total RNA was extracted from RAW264.7 cells after 4 h of treatment using the FastPure Cell/Tissue Total RNA Isolation Kit V2 (Vazyme, China). Reverse transcription was performed with the TransScript Uni All-in-One First-Strand cDNA Synthesis SuperMix (TransGen, China), and quantitative PCR was conducted using PerfectStart Visual Green qPCR SuperMix (TransGen, China) on a LightCycler 480 Instrument II (Roche, Switzerland). Cycling conditions were: 95 °C for 1 min, followed by 40 cycles of 95 °C for 5 s, 60 °C for 15 s, and 72 °C for 10 s. Relative gene expression was analyzed using the $2^{-\Delta\Delta Ct}$ method, with ACTIN as the internal control. Primer sequences are listed in Table S3.

**Table S-3 List of the primers.**

| Primer name | Primer sequence (5'→3') |
|---|---|
| IL-6-F | TACTCGGCAAACCTAGTGCG |
| IL-6-R | GTGTCCCAACATTCATATTGTCAGT |
| IL-1β-F | TCGTGCTGTCGGACCCATAT |
| IL-1β-R | GTCGTTGCTTGGTTCTCCTTGT |
| TNF-α-F | CGAGGCTCCAGTGAATTCGG |
| TNF-α-R | GGGGATTATGGCTCAGGGTC |
| Actin-F | GGCTGTATTCCCCTCCATCG |
| Actin-R | CCAGTTGGTAACAATGCCATGT |

Nitric Oxide (NO) Assay

The effect of compounds on NO production was determined using a Nitric Oxide Assay Kit

(Beyotime, China). RAW264.7 cells were stimulated with LPS (1 μg/mL) for 24 h in the presence or absence of compounds, and the supernatants were collected for NO quantification according to the manufacturer’s instructions.

Enzyme-Linked Immunosorbent Assay (ELISA)

Levels of IL-6 and TNF-α in culture supernatants were quantified using ELISA kits (JONLNBIO, China). RAW264.7 cells were treated with LPS (1 μg/mL) for 24 h in the presence or absence of compounds, and cytokine concentrations were measured following the manufacturer’s instructions.

**Results**

Except for musellarin **F**, all compounds had minimal impact on RAW264.7 cell viability (Figure S1A). Therefore, musellarin **F** was excluded from subsequent screening of anti-inflammatory activity. Upon LPS stimulation, IL-6, IL-1β, and TNF-α increased markedly (Figure S1B-D; dotted lines indicate the model baseline). Relative to the LPS group, most musellarin treatments exhibited inhibitory effects on these mediators, with the strongest reductions observed for musellarin **A**, **B**, **C**, **9I**, and **9J**.

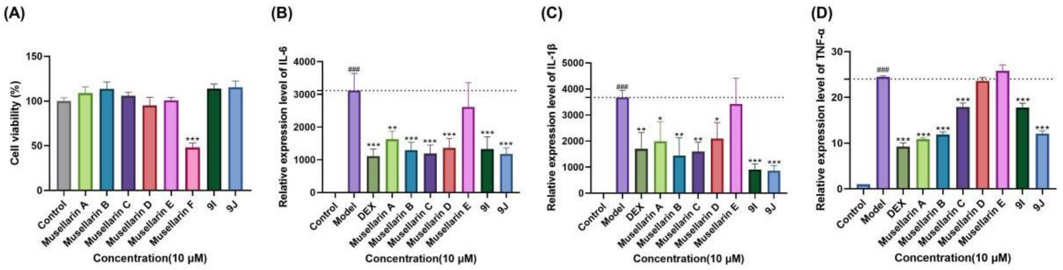


Figure S1. Effects of musellarin **A-F, 9I** and **9J** on cell viability and inflammatory mediators in LPS-induced RAW264.7 macrophages. (A) Cell viability was determined by CCK-8 assay after 24 h treatment with musellarin **A-F, 9I** and **9J**. Statistical significance was determined versus the Control group (***p < 0.001). (B-D) The effects of musellarin **A-F**, **9I** and **9J** on LPS-induced IL-6, IL-1β and TNF-α expression, respectively. Data are presented as mean ± SD (n = 3). Statistical significance was determined versus the Control group (###p < 0.001), and the LPS group (*p < 0.05, **p < 0.01, ***p < 0.001).

Across 0.625-25 μM, musellarin **A, B, C, 9I**, and **9J** maintained RAW264.7 cell viability at ≥ 90% (Figure S2A), indicating no significantly cytotoxicity within the tested range. Musellarin **A-C** showed only modest effects, with $IC_{50}$ values exceeding 25 μM for NO, IL-6, and TNF-α. On contrast, compounds **9I**, and **9J** suppressed these production in a dose-dependent manner. The $IC_{50}$ values were 13.2 ± 1.95 μM (NO), 18.4 ± 4.82 μM (IL-6), and 17.74± 4.48 μM (TNF-α) for **9I**, and 9.13 ± 1.33 μM (NO), 13.21 ± 1.41 μM (IL-6), and 10.68 ± 2.2 μM (TNF-α) for **9J**.

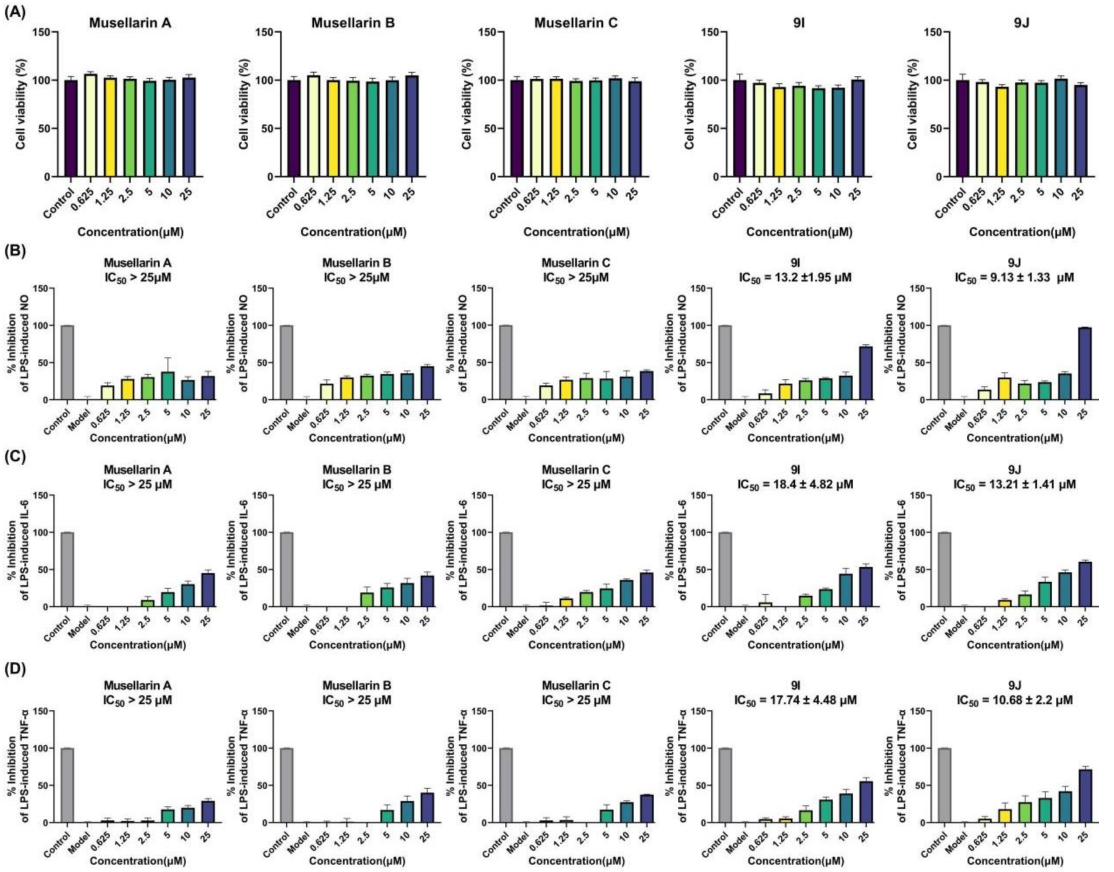


Figure S2. Effects of musellarin **A-C**, **9I**, and **9J** on cell viability and LPS-induced inflammatory mediator production in RAW264.7 macrophages. (A) Cell viability of RAW264.7 cells treated with musellarin **A-C**, **9I**, and **9J** at different concentrations (0.625-25 μM). (B-D) Inhibitory effects of musellarin **A-C**, **9I**, and **9J** on LPS-induced NO, IL-6, and TNF-α production in RAW264.7 macrophages. Data are expressed as mean ± SD (n = 3).

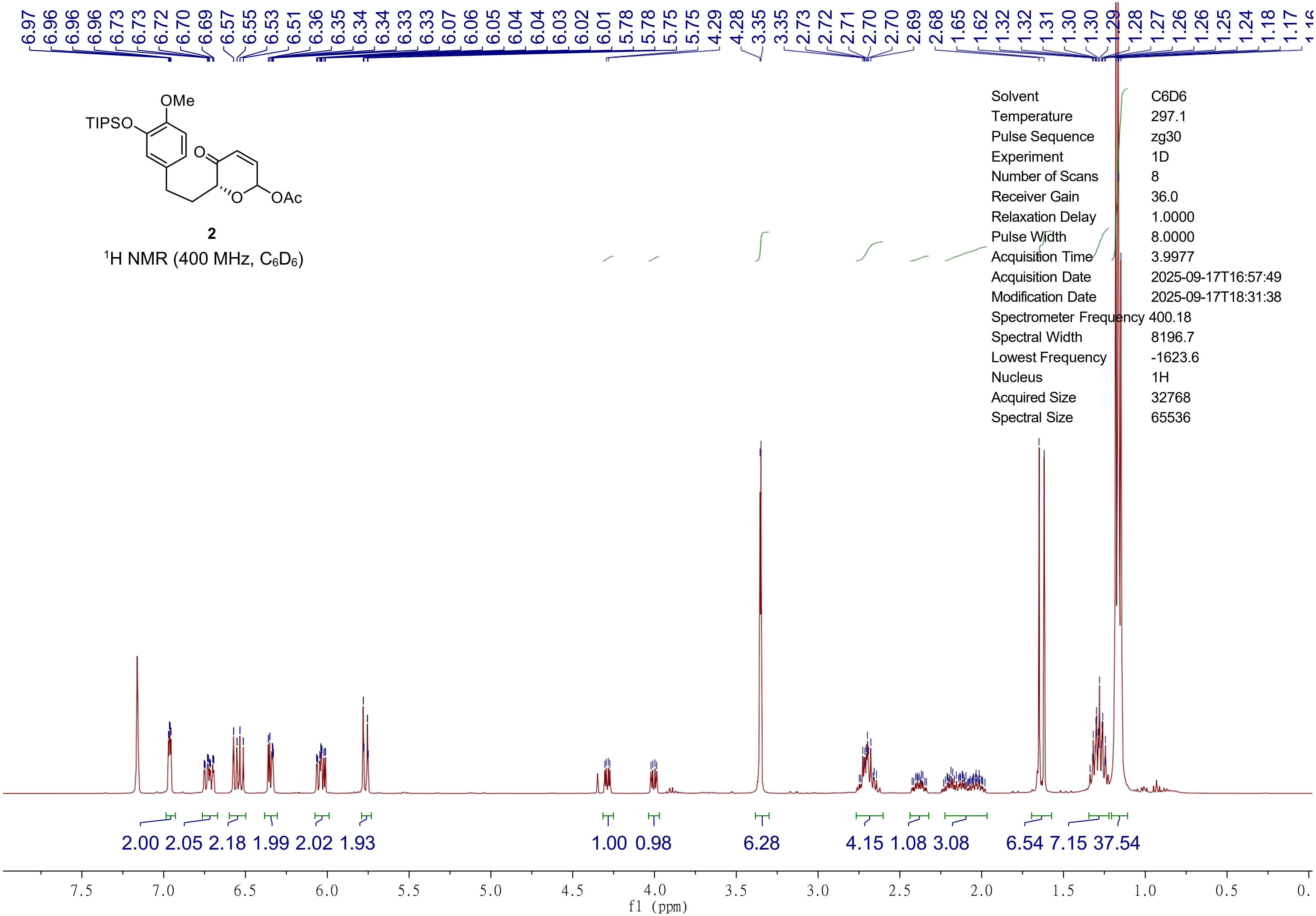


**2**

$^1$H NMR (400 MHz, $C_6D_6$)

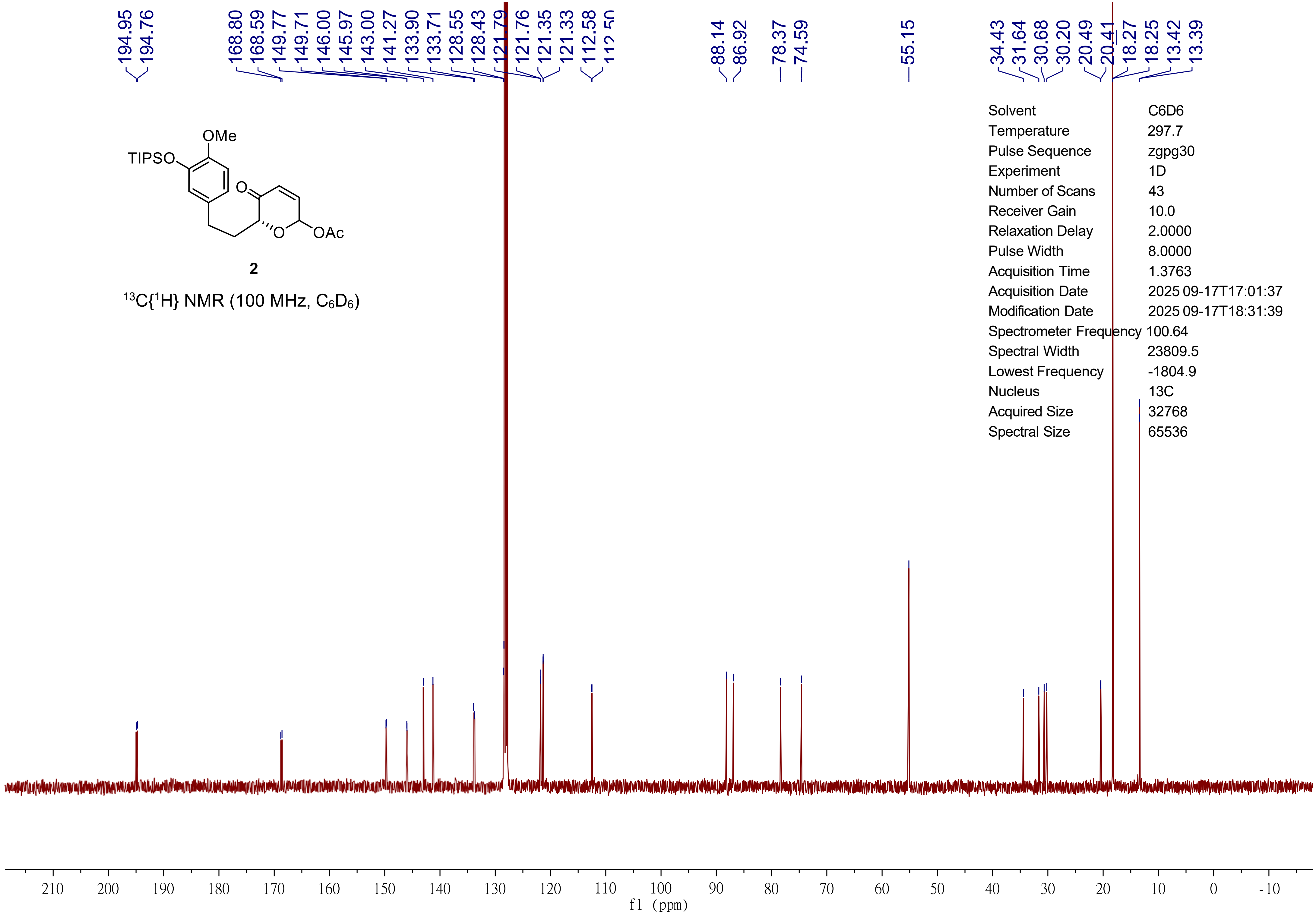

194.95
194.76
168.80
168.59
149.77
149.71
146.00
145.97
143.00
141.27
133.90
133.71
128.55
128.43
121.79
121.76
121.35
121.33
112.58
88.14
86.92
78.37
74.59
55.15
34.43
31.64
30.68
30.20
20.49
20.41
18.27
18.25
13.42
13.39
OMe
TIPSO
O
O
OAc
2
13C{1H} NMR (100 MHz, C6D6)
Solvent C6D6
Temperature 297.7
Pulse Sequence zgpg30
Experiment 1D
Number of Scans 43
Receiver Gain 10.0
Relaxation Delay 2.0000
Pulse Width 8.0000
Acquisition Time 1.3763
Acquisition Date 2025 09-17T17:01:37
Modification Date 2025 09-17T18:31:39
Spectrometer Frequency 100.64
Spectral Width 23809.5
Lowest Frequency -1804.9
Nucleus 13C
Acquired Size 32768
Spectral Size 65536
210 200 190 180 170 160 150 140 130 120 110 100 90 80 70 60 50 40 30 20 10 0 -10
f1 (ppm)

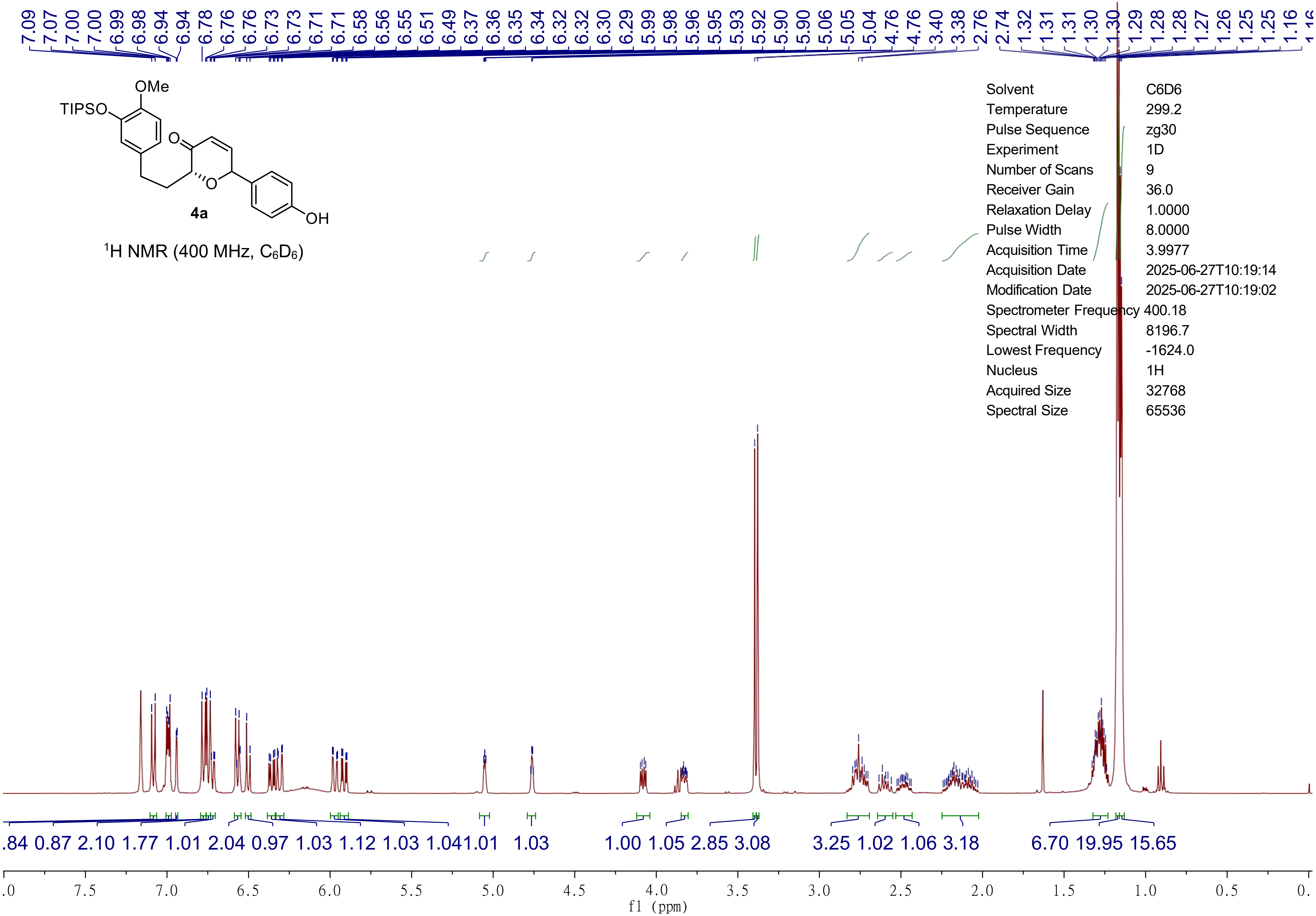


$^1$H NMR (400 MHz, $C_6D_6$)

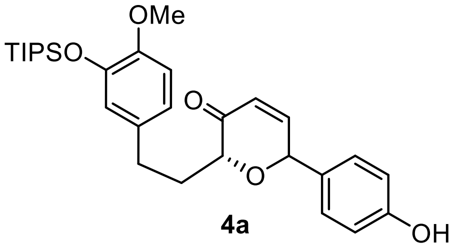

**4a**

$^{13}C\{^1H\}$ NMR (100 MHz, $C_6D_6$)

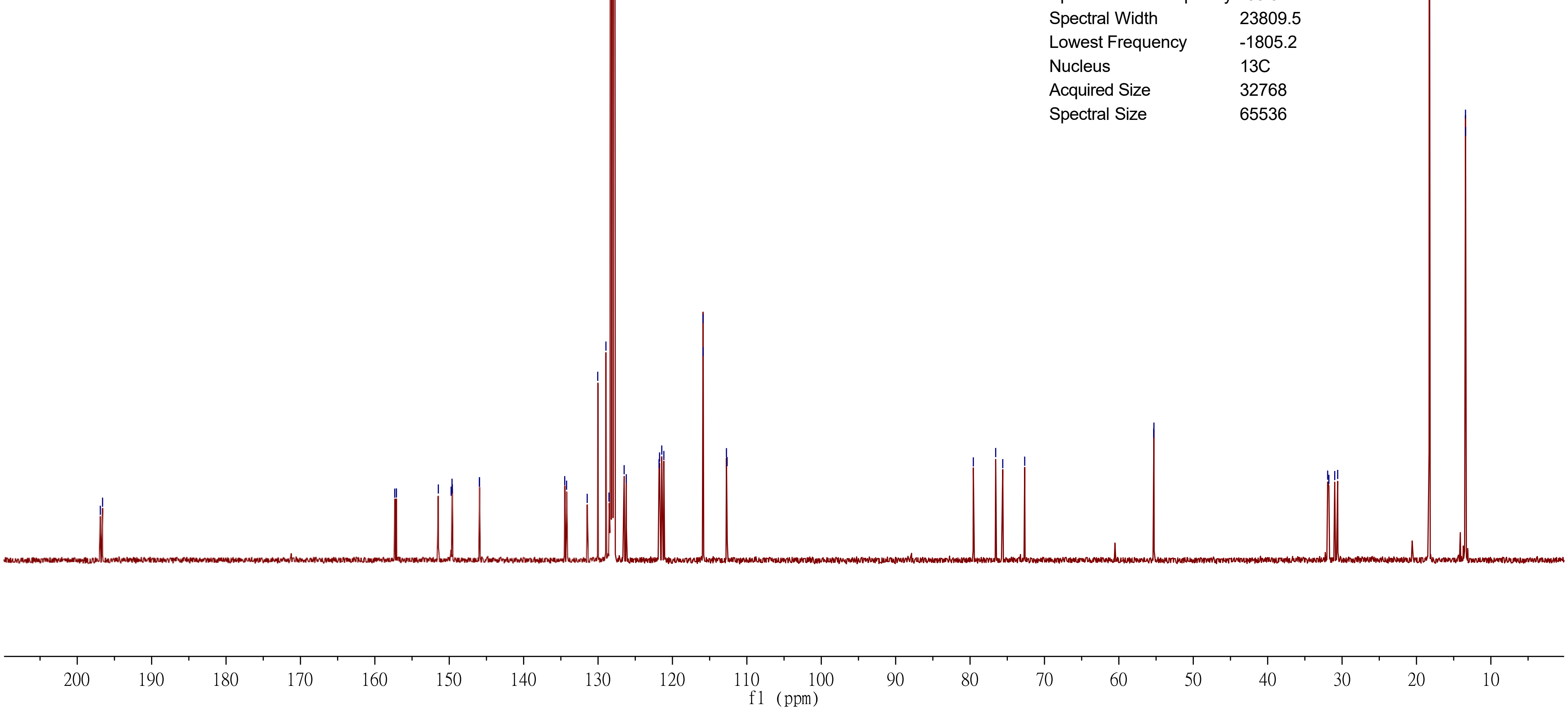


| | |
|---|---|
| Solvent | C6D6 |
| Temperature | 300.1 |
| Pulse Sequence | zgpg30 |
| Experiment | 1D |
| Number of Scans | 400 |
| Receiver Gain | 10.0 |
| Relaxation Delay | 2.0000 |
| Pulse Width | 8.0000 |
| Acquisition Time | 1.3763 |
| Acquisition Date | 2025-06-27T17:49:23 |
| Modification Date | 2025-06-27T17:49:12 |
| Spectrometer Frequency | 100.64 |
| Spectral Width | 23809.5 |
| Lowest Frequency | -1805.2 |
| Nucleus | 13C |
| Acquired Size | 32768 |
| Spectral Size | 65536 |

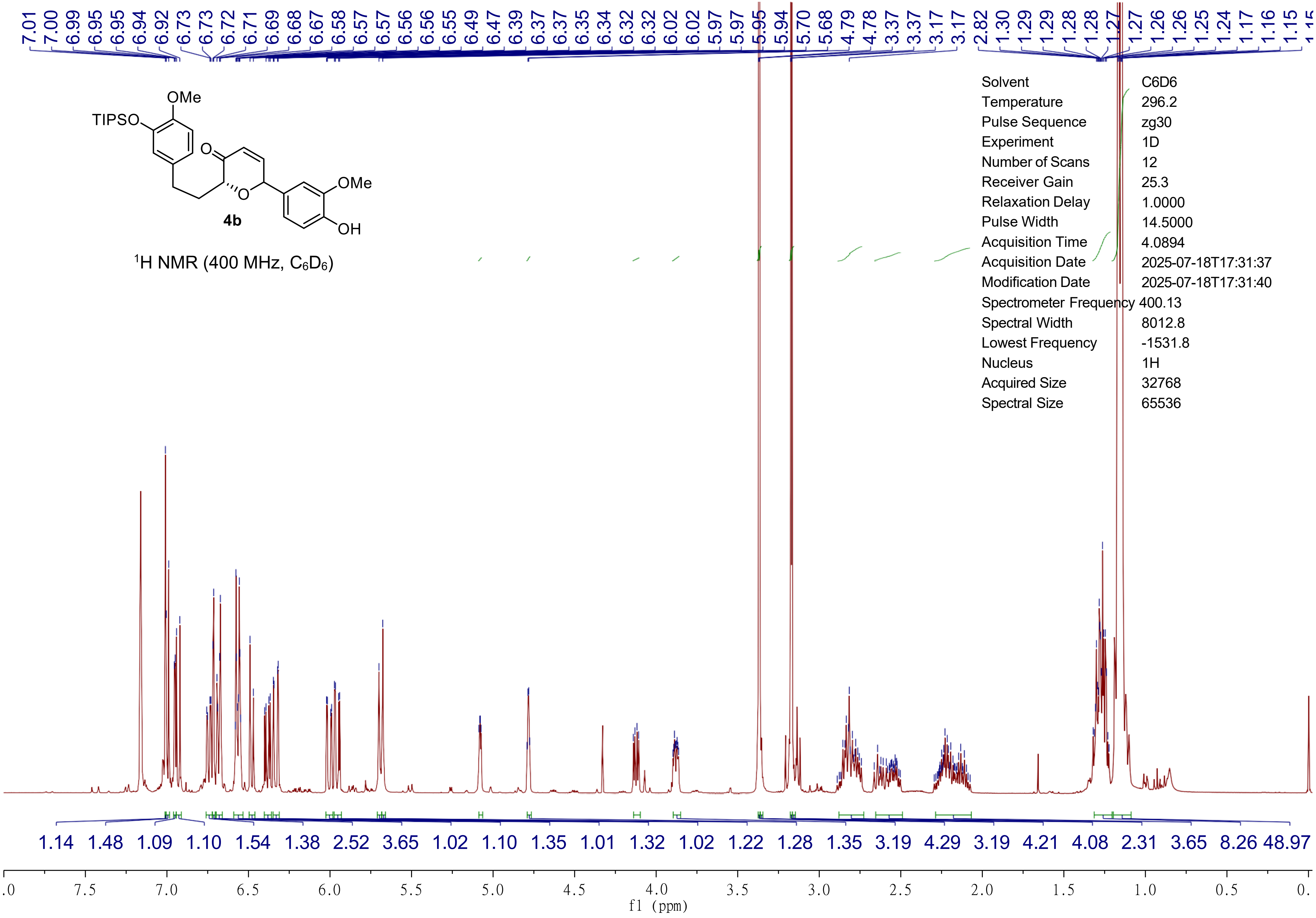

TIPSO
OMe
O
OMe
O
OH
4b
$^{1}$H NMR (400 MHz, $C_6D_6$)
Solvent C6D6
Temperature 296.2
Pulse Sequence zg30
Experiment 1D
Number of Scans 12
Receiver Gain 25.3
Relaxation Delay 1.0000
Pulse Width 14.5000
Acquisition Time 4.0894
Acquisition Date 2025-07-18T17:31:37
Modification Date 2025-07-18T17:31:40
Spectrometer Frequency 400.13
Spectral Width 8012.8
Lowest Frequency -1531.8
Nucleus 1H
Acquired Size 32768
Spectral Size 65536
1.14 1.48 1.09 1.10 1.54 1.38 2.52 3.65 1.02 1.10 1.35 1.01 1.32 1.02 1.22 1.28 1.35 3.19 4.29 3.19 4.21 4.08 2.31 3.65 8.26 48.97
f1 (ppm)

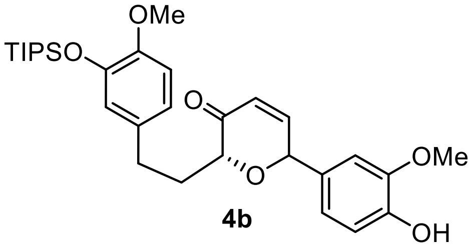


**4b**

$^{13}C\{^{1}H\}$ NMR (100 MHz, $C_6D_6$)

| | |
|---|---|
| Solvent | C6D6 |
| Temperature | 297.4 |
| Pulse Sequence | zgpg30 |
| Experiment | 1D |
| Number of Scans | 466 |
| Receiver Gain | 196.9 |
| Relaxation Delay | 2.0000 |
| Pulse Width | 9.7000 |
| Acquisition Time | 1.3631 |
| Acquisition Date | 2025-07-18T17:59:43 |
| Modification Date | 2025-07-18T17:59:46 |
| Spectrometer Frequency | 100.62 |
| Spectral Width | 24038.5 |
| Lowest Frequency | -1920.7 |
| Nucleus | 13C |
| Acquired Size | 32768 |
| Spectral Size | 65536 |

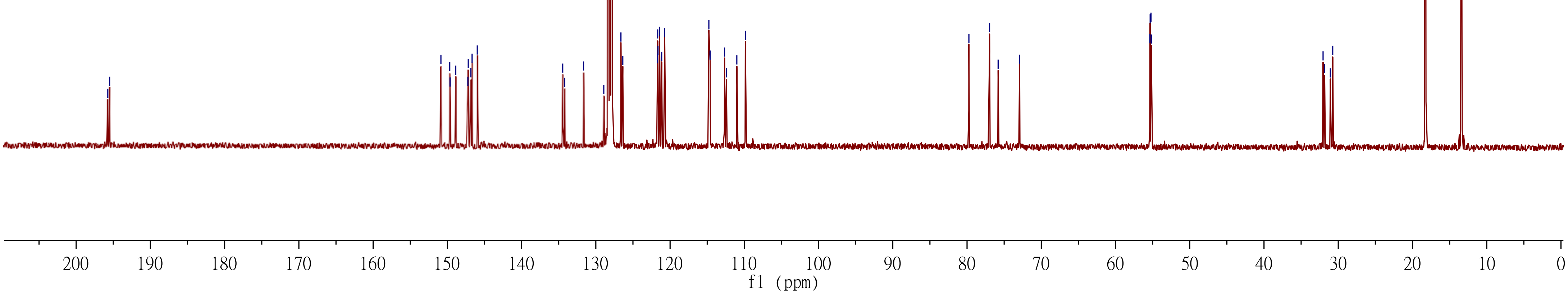

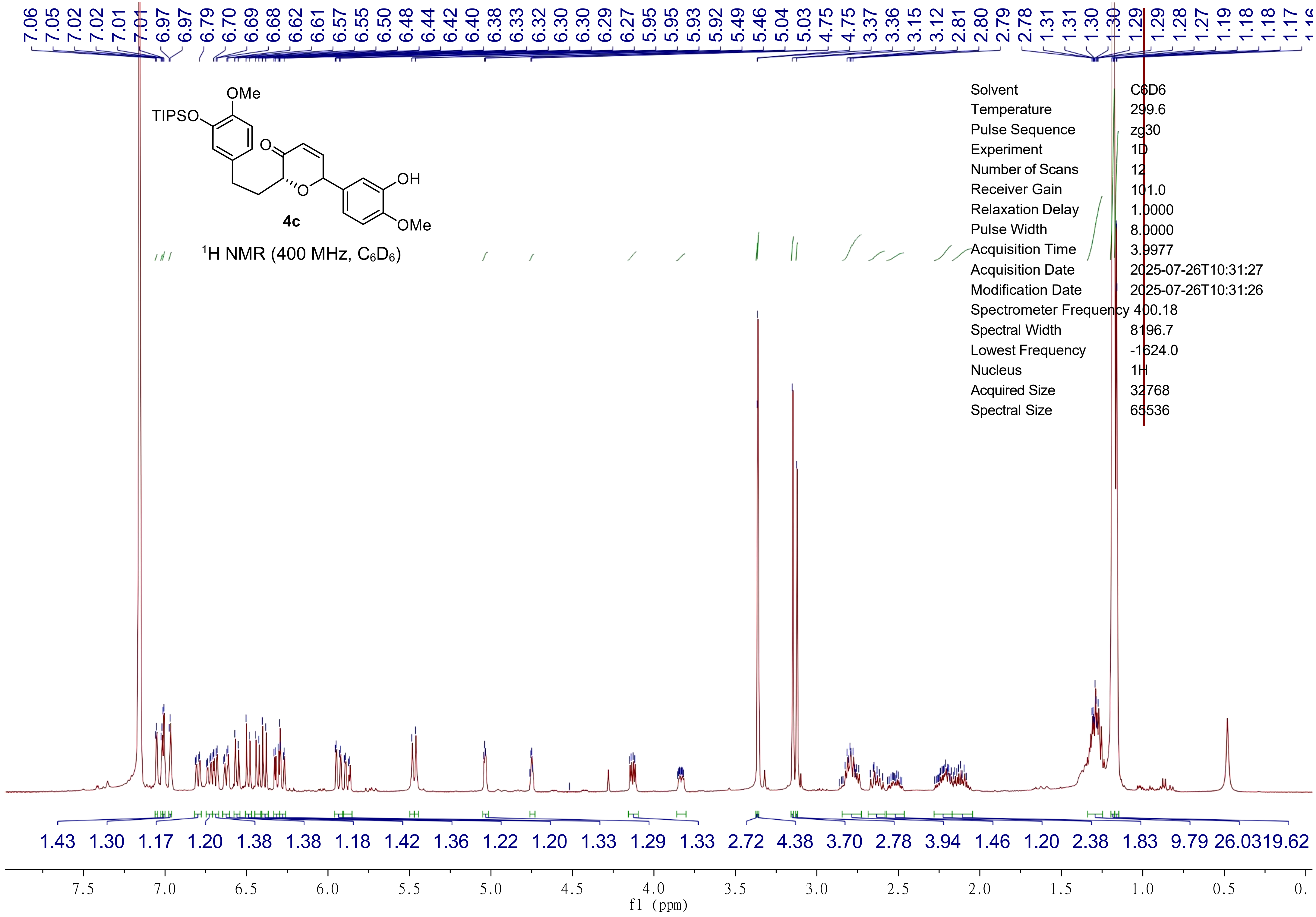

TIPSO
OMe
O
OH
O
OMe
4c
$^{1}$H NMR (400 MHz, $C_6D_6$)
Solvent C6D6
Temperature 299.6
Pulse Sequence zg30
Experiment 1D
Number of Scans 12
Receiver Gain 101.0
Relaxation Delay 1.0000
Pulse Width 8.0000
Acquisition Time 3.9977
Acquisition Date 2025-07-26T10:31:27
Modification Date 2025-07-26T10:31:26
Spectrometer Frequency 400.18
Spectral Width 8196.7
Lowest Frequency -1624.0
Nucleus 1H
Acquired Size 32768
Spectral Size 65536
7.06 7.05 7.02 7.02 7.01 7.01 6.97 6.97 6.79 6.70 6.69 6.68 6.62 6.61 6.57 6.55 6.50 6.48 6.44 6.42 6.40 6.38 6.33 6.32 6.30 6.30 6.29 6.27 5.95 5.95 5.93 5.92 5.49 5.46 5.04 5.03 4.75 4.75 3.37 3.36 3.15 3.12 2.81 2.80 2.79 2.78 1.31 1.31 1.30 1.30 1.29 1.29 1.28 1.27 1.19 1.18 1.18 1.17
1.43 1.30 1.17 1.20 1.38 1.38 1.18 1.42 1.36 1.22 1.20 1.33 1.29 1.33 2.72 4.38 3.70 2.78 3.94 1.46 1.20 2.38 1.83 9.79 26.03 19.62
7.5 7.0 6.5 6.0 5.5 5.0 4.5 4.0 3.5 3.0 2.5 2.0 1.5 1.0 0.5
f1 (ppm)

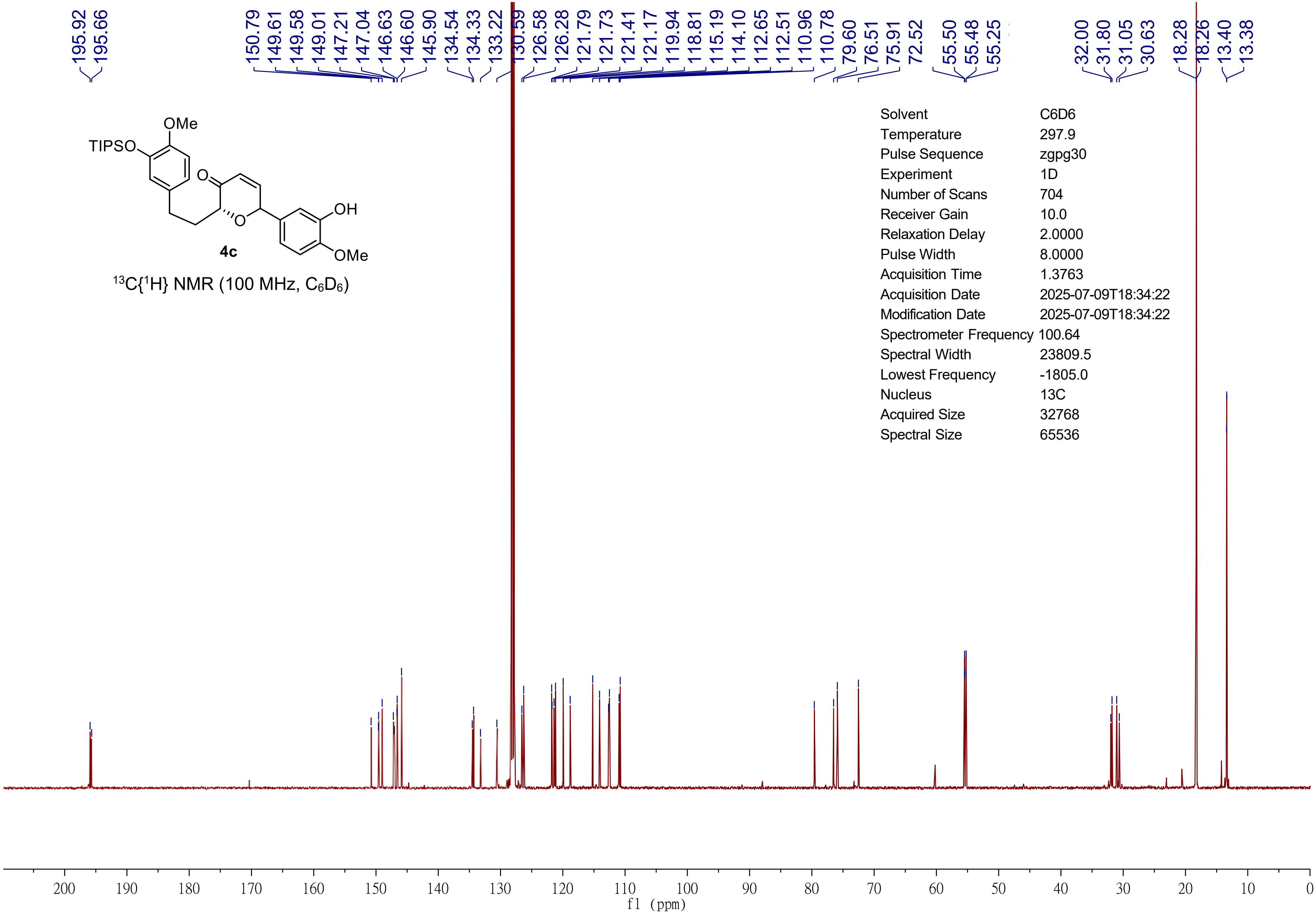
195.92
195.66
150.79
149.61
149.58
149.01
147.21
147.04
146.63
146.60
145.90
134.54
134.33
133.22
130.59
126.58
126.28
121.79
121.73
121.41
121.17
119.94
118.81
115.19
114.10
112.65
112.51
110.96
110.78
79.60
76.51
75.91
72.52
55.50
55.48
55.25
32.00
31.80
31.05
30.63
18.28
18.26
13.40
13.38
OMe
TIPSO
O
OH
O
OMe
4c
$^{13}C\{^{1}H\}$ NMR (100 MHz, $C_6D_6$)
Solvent C6D6
Temperature 297.9
Pulse Sequence zgpg30
Experiment 1D
Number of Scans 704
Receiver Gain 10.0
Relaxation Delay 2.0000
Pulse Width 8.0000
Acquisition Time 1.3763
Acquisition Date 2025-07-09T18:34:22
Modification Date 2025-07-09T18:34:22
Spectrometer Frequency 100.64
Spectral Width 23809.5
Lowest Frequency -1805.0
Nucleus 13C
Acquired Size 32768
Spectral Size 65536
200 190 180 170 160 150 140 130 120 110 100 90 80 70 60 50 40 30 20 10 0
f1 (ppm)

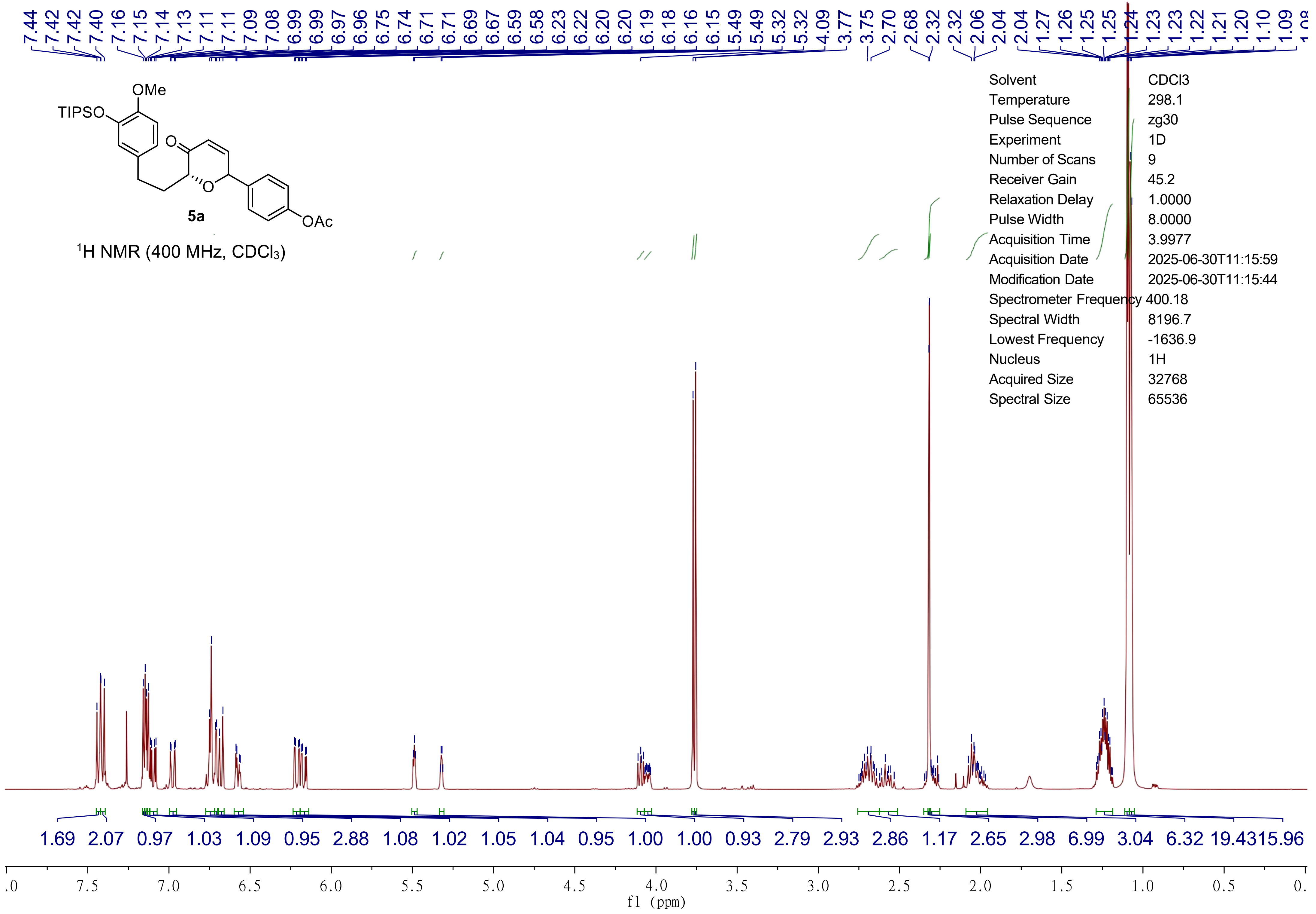

OMe
TIPSO
O
O
OAc
5a
$^{1}$H NMR (400 MHz, CDCl$_3$)
Solvent CDCl3
Temperature 298.1
Pulse Sequence zg30
Experiment 1D
Number of Scans 9
Receiver Gain 45.2
Relaxation Delay 1.0000
Pulse Width 8.0000
Acquisition Time 3.9977
Acquisition Date 2025-06-30T11:15:59
Modification Date 2025-06-30T11:15:44
Spectrometer Frequency 400.18
Spectral Width 8196.7
Lowest Frequency -1636.9
Nucleus 1H
Acquired Size 32768
Spectral Size 65536
7.44 7.42 7.42 7.40 7.16 7.15 7.14 7.13 7.11 7.11 7.09 7.08 6.99 6.99 6.97 6.96 6.75 6.74 6.71 6.71 6.69 6.67 6.59 6.58 6.23 6.22 6.20 6.20 6.19 6.18 6.16 6.15 5.49 5.49 5.32 5.32 4.09 3.77 3.75 2.70 2.68 2.32 2.32 2.06 2.04 2.04 1.27 1.26 1.25 1.25 1.24 1.23 1.23 1.22 1.21 1.20 1.10 1.09
1.69 2.07 0.97 1.03 1.09 0.95 2.88 1.08 1.02 1.05 1.04 0.95 1.00 1.00 0.93 2.79 2.93 2.86 1.17 2.65 2.98 6.99 3.04 6.32 19.43 15.96
7.5 7.0 6.5 6.0 5.5 5.0 4.5 4.0 3.5 3.0 2.5 2.0 1.5 1.0 0.5
f1 (ppm)

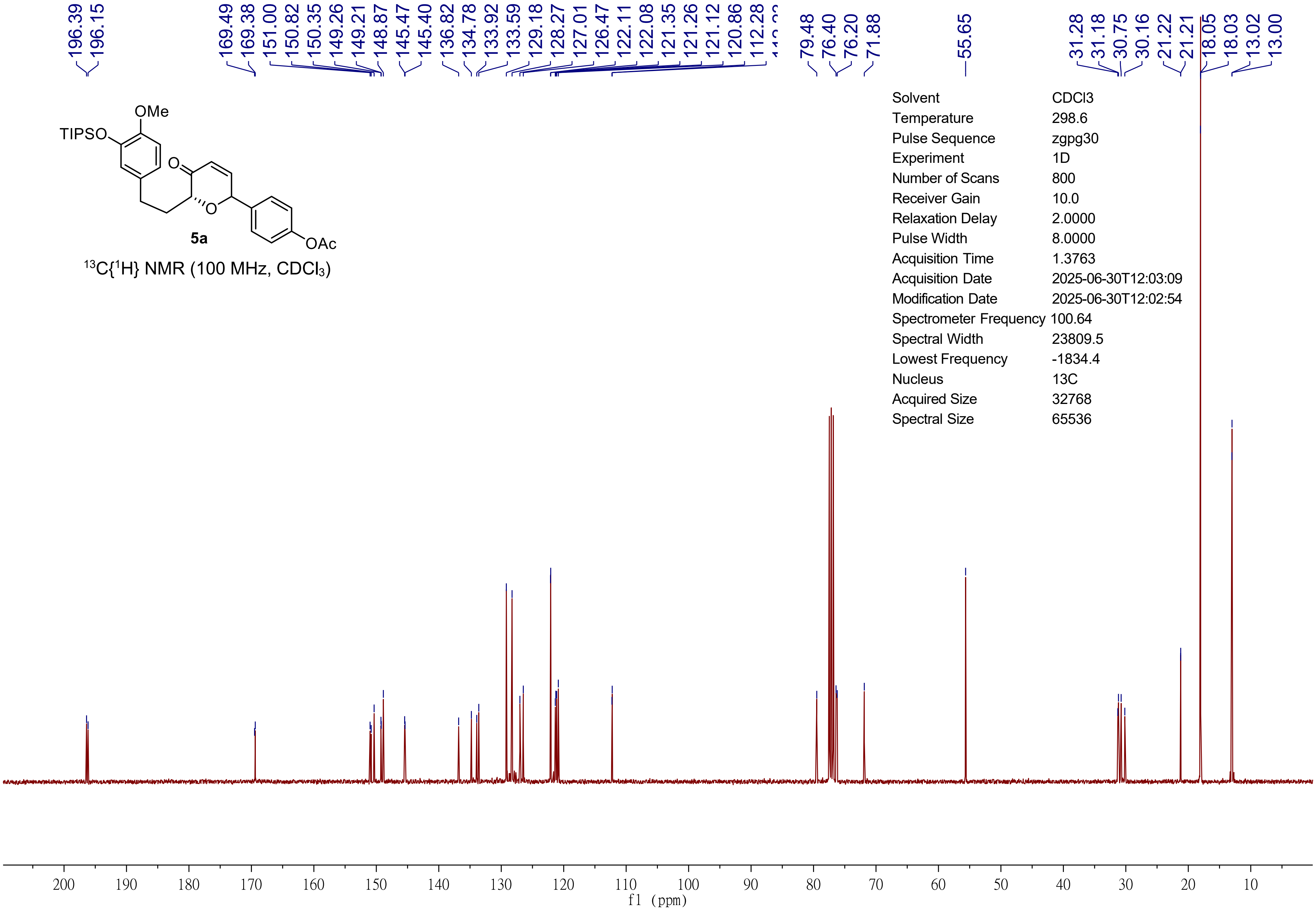
196.39
196.15
169.49
169.38
151.00
150.82
150.35
149.26
149.21
148.87
145.47
145.40
136.82
134.78
133.92
133.59
129.18
128.27
127.01
126.47
122.11
122.08
121.35
121.26
121.12
120.86
112.28
79.48
76.40
76.20
71.88
55.65
31.28
31.18
30.75
30.16
21.22
21.21
18.05
18.03
13.02
13.00
OMe
TIPSO
O
O
OAc
5a
13C{1H} NMR (100 MHz, CDCl3)
Solvent CDCl3
Temperature 298.6
Pulse Sequence zgpg30
Experiment 1D
Number of Scans 800
Receiver Gain 10.0
Relaxation Delay 2.0000
Pulse Width 8.0000
Acquisition Time 1.3763
Acquisition Date 2025-06-30T12:03:09
Modification Date 2025-06-30T12:02:54
Spectrometer Frequency 100.64
Spectral Width 23809.5
Lowest Frequency -1834.4
Nucleus 13C
Acquired Size 32768
Spectral Size 65536
200 190 180 170 160 150 140 130 120 110 100 90 80 70 60 50 40 30 20 10
f1 (ppm)

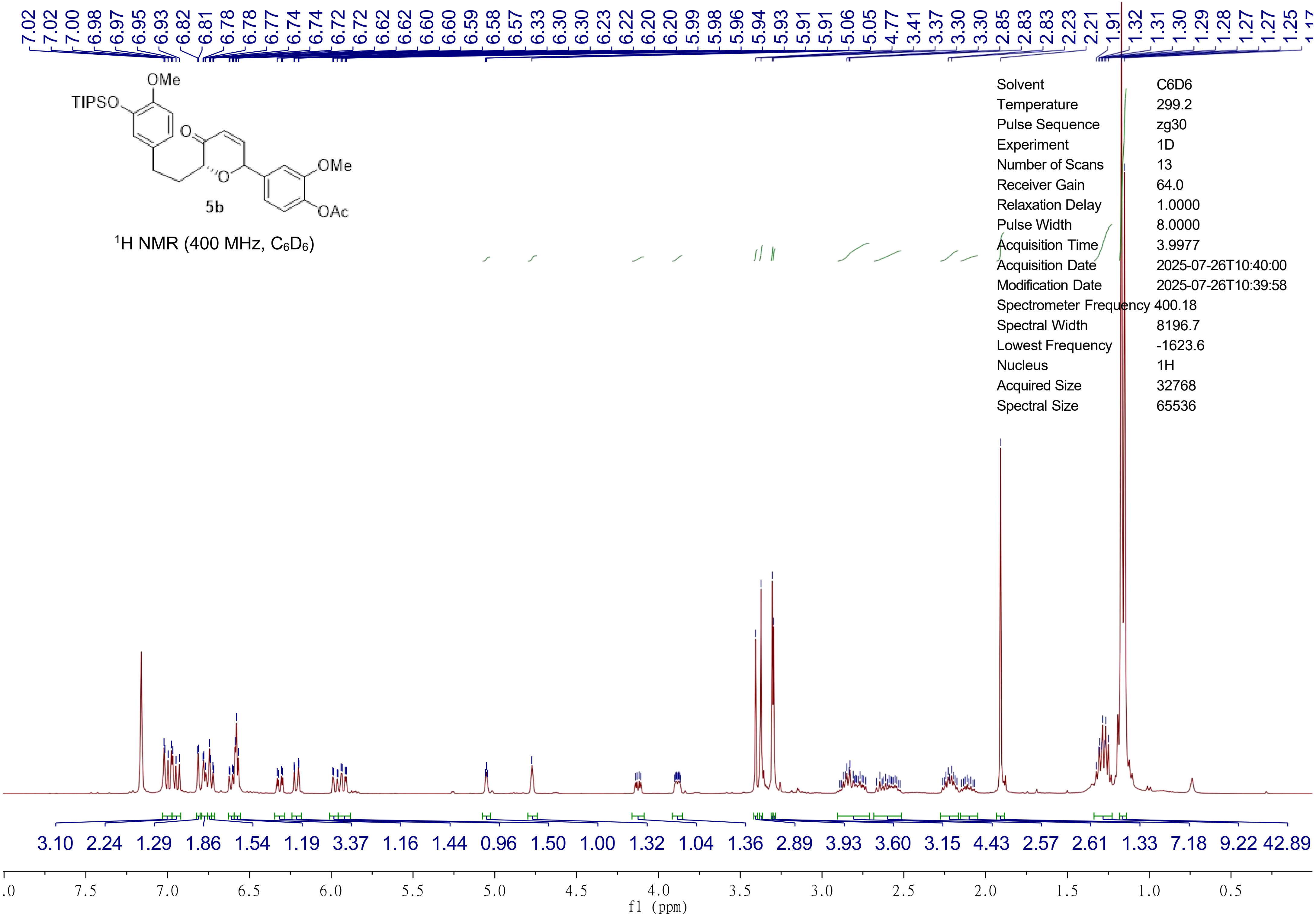


$^{1}$H NMR (400 MHz, $C_6D_6$)

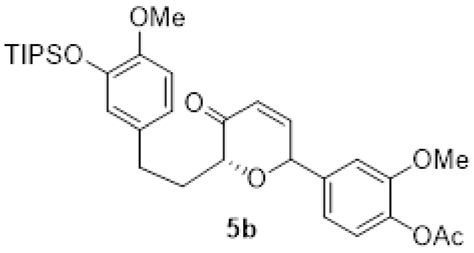


5b

$^{13}C\{^{1}H\}$ NMR (100 MHz, $C_6D_6$)

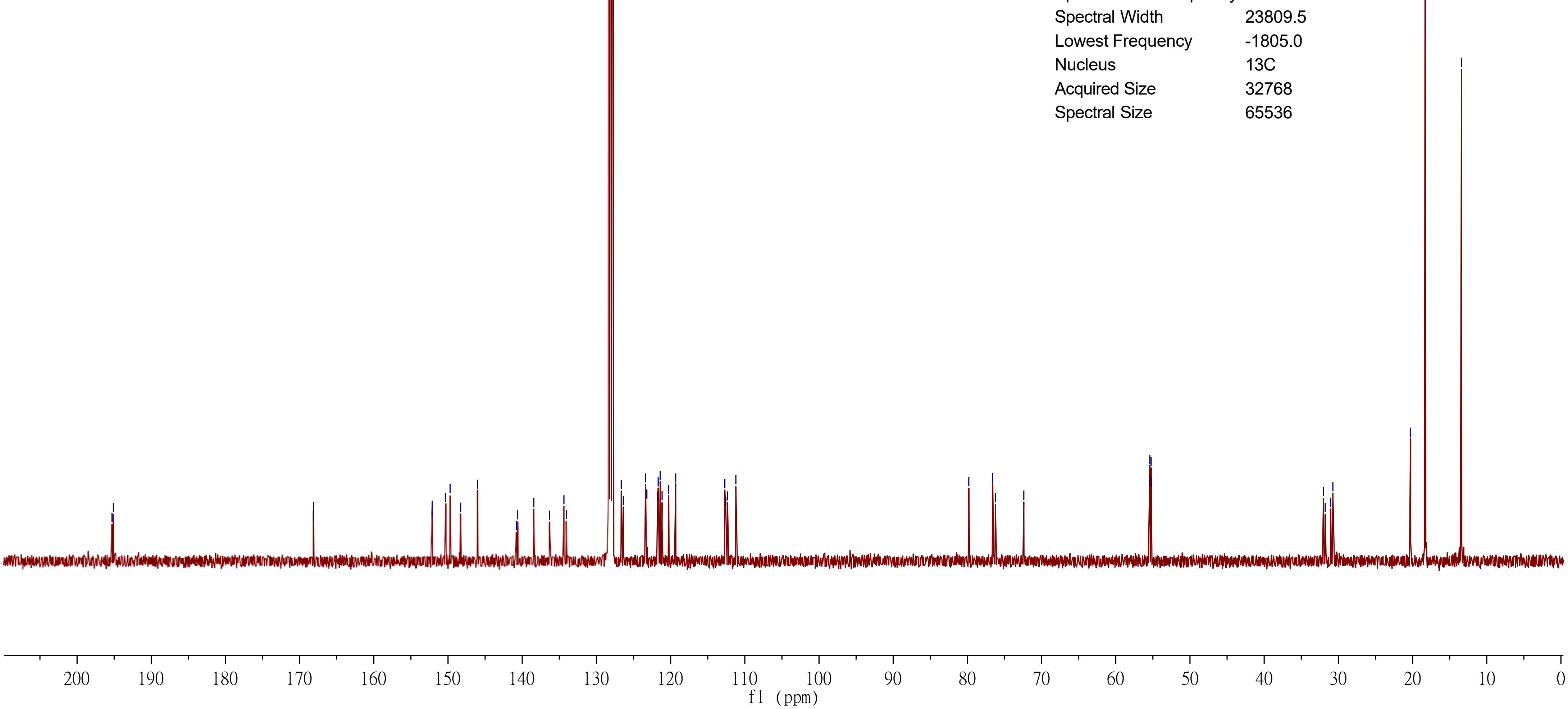


| | |
|---|---|
| Solvent | C6D6 |
| Temperature | 300.1 |
| Pulse Sequence | zgpg30 |
| Experiment | 1D |
| Number of Scans | 101 |
| Receiver Gain | 10.0 |
| Relaxation Delay | 2.0000 |
| Pulse Width | 8.0000 |
| Acquisition Time | 1.3763 |
| Acquisition Date | 2025-07-26T10:47:10 |
| Modification Date | 2025-07-26T10:47:08 |
| Spectrometer Frequency | 100.64 |
| Spectral Width | 23809.5 |
| Lowest Frequency | -1805.0 |
| Nucleus | 13C |
| Acquired Size | 32768 |
| Spectral Size | 65536 |

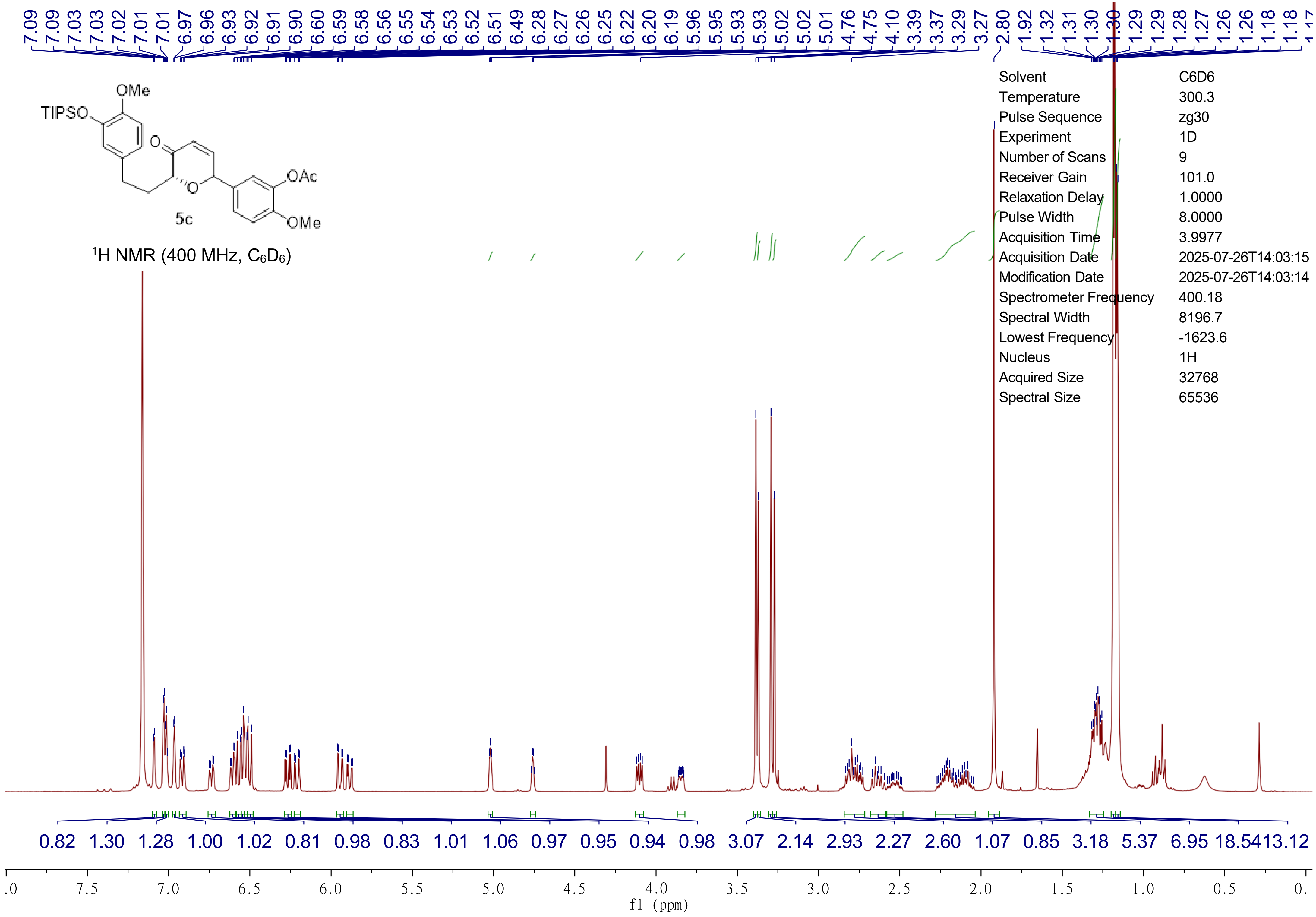

TIPSO
OMe
OAc
OMe
5c
$^1$H NMR (400 MHz, $C_6D_6$)
Solvent C6D6
Temperature 300.3
Pulse Sequence zg30
Experiment 1D
Number of Scans 9
Receiver Gain 101.0
Relaxation Delay 1.0000
Pulse Width 8.0000
Acquisition Time 3.9977
Acquisition Date 2025-07-26T14:03:15
Modification Date 2025-07-26T14:03:14
Spectrometer Frequency 400.18
Spectral Width 8196.7
Lowest Frequency -1623.6
Nucleus 1H
Acquired Size 32768
Spectral Size 65536
f1 (ppm)

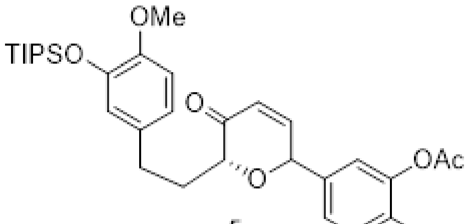

5c

$^{13}C\{^{1}H\}$ NMR (100 MHz, $C_6D_6$)

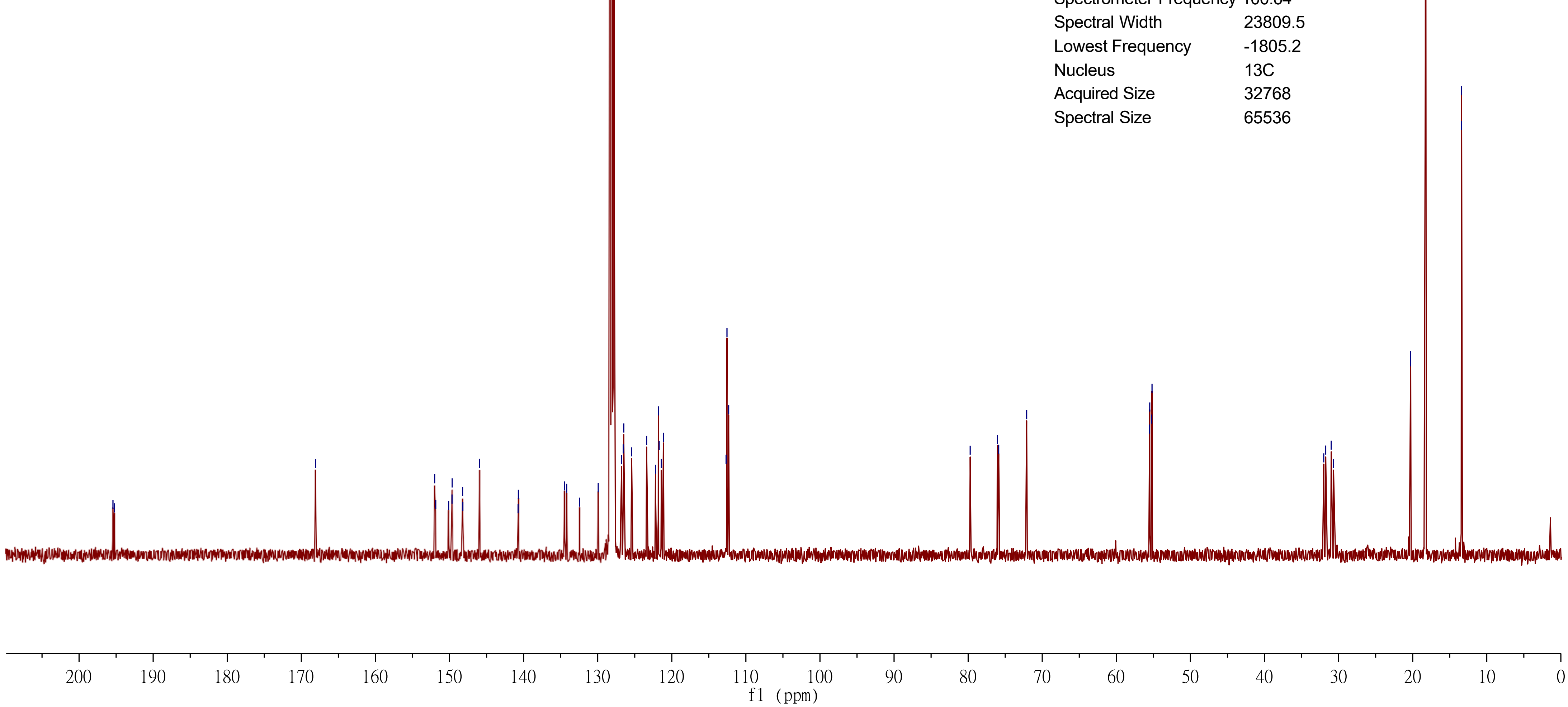


| | |
|---|---|
| Solvent | C6D6 |
| Temperature | 298.2 |
| Pulse Sequence | zgpg30 |
| Experiment | 1D |
| Number of Scans | 805 |
| Receiver Gain | 10.0 |
| Relaxation Delay | 2.0000 |
| Pulse Width | 8.0000 |
| Acquisition Time | 1.3763 |
| Acquisition Date | 2025-07-10T20:00:54 |
| Modification Date | 2025-07-10T20:00:54 |
| Spectrometer Frequency | 100.64 |
| Spectral Width | 23809.5 |
| Lowest Frequency | -1805.2 |
| Nucleus | 13C |
| Acquired Size | 32768 |
| Spectral Size | 65536 |

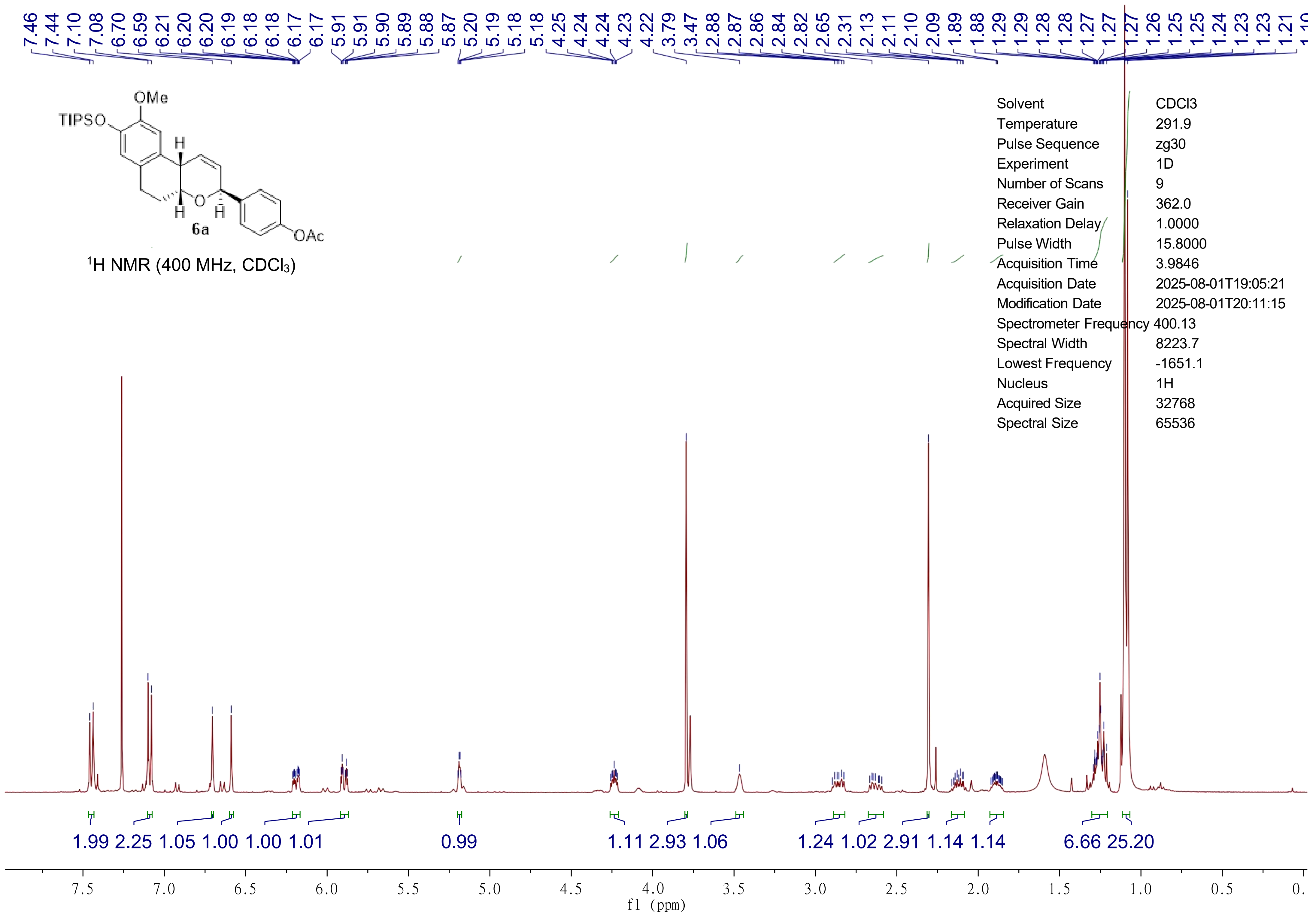

7.46 7.44 7.10 7.08 6.70 6.59 6.21 6.20 6.20 6.19 6.18 6.18 6.17 6.17 5.91 5.91 5.90 5.89 5.88 5.87 5.20 5.19 5.18 5.18 4.25 4.24 4.24 4.23 4.22 3.79 3.47 2.88 2.87 2.86 2.84 2.82 2.65 2.31 2.13 2.11 2.10 2.09 1.89 1.88 1.29 1.29 1.28 1.28 1.27 1.27 1.27 1.26 1.25 1.25 1.24 1.23 1.23 1.21
OMe
TIPSO
H
H
H
O
OAc
6a
1H NMR (400 MHz, CDCl3)
Solvent CDCl3
Temperature 291.9
Pulse Sequence zg30
Experiment 1D
Number of Scans 9
Receiver Gain 362.0
Relaxation Delay 1.0000
Pulse Width 15.8000
Acquisition Time 3.9846
Acquisition Date 2025-08-01T19:05:21
Modification Date 2025-08-01T20:11:15
Spectrometer Frequency 400.13
Spectral Width 8223.7
Lowest Frequency -1651.1
Nucleus 1H
Acquired Size 32768
Spectral Size 65536
1.99 2.25 1.05 1.00 1.00 1.01 0.99 1.11 2.93 1.06 1.24 1.02 2.91 1.14 1.14 6.66 25.20
7.5 7.0 6.5 6.0 5.5 5.0 4.5 4.0 3.5 3.0 2.5 2.0 1.5 1.0 0.5
f1 (ppm)

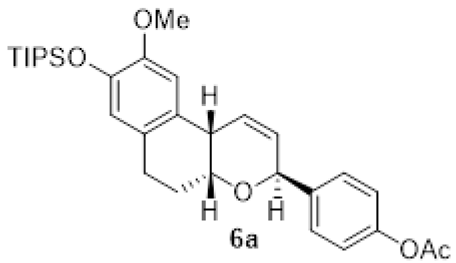


6a

$^{13}C\{^{1}H\}$ NMR (100 MHz, $CDCl_3$)

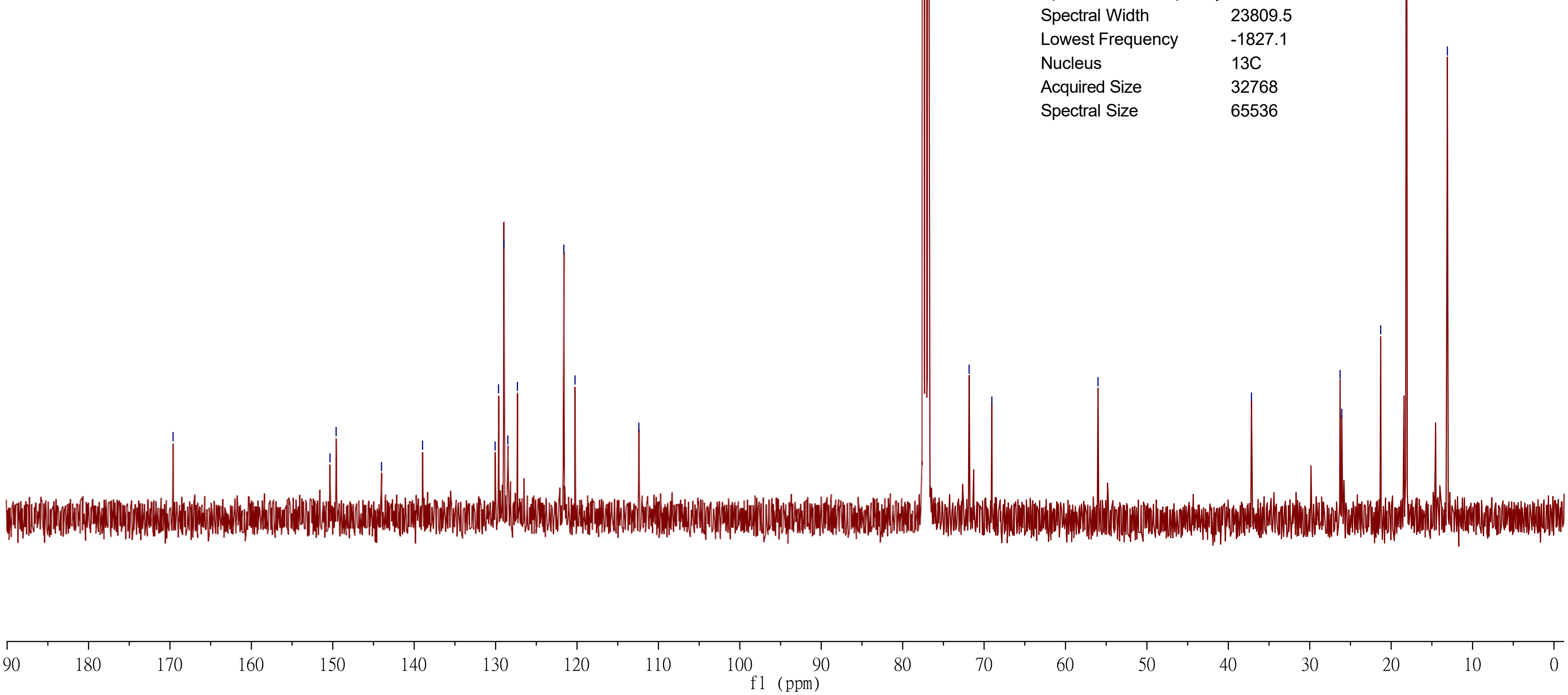


| | |
|---|---|
| Solvent | CDCl3 |
| Temperature | 302.6 |
| Pulse Sequence | zgpg30 |
| Experiment | 1D |
| Number of Scans | 5000 |
| Receiver Gain | 16.0 |
| Relaxation Delay | 2.0000 |
| Pulse Width | 8.0000 |
| Acquisition Time | 1.3763 |
| Acquisition Date | 2025-08-11T08:44:23 |
| Modification Date | 2025-08-29T15:25:45 |
| Spectrometer Frequency | 100.64 |
| Spectral Width | 23809.5 |
| Lowest Frequency | -1827.1 |
| Nucleus | 13C |
| Acquired Size | 32768 |
| Spectral Size | 65536 |

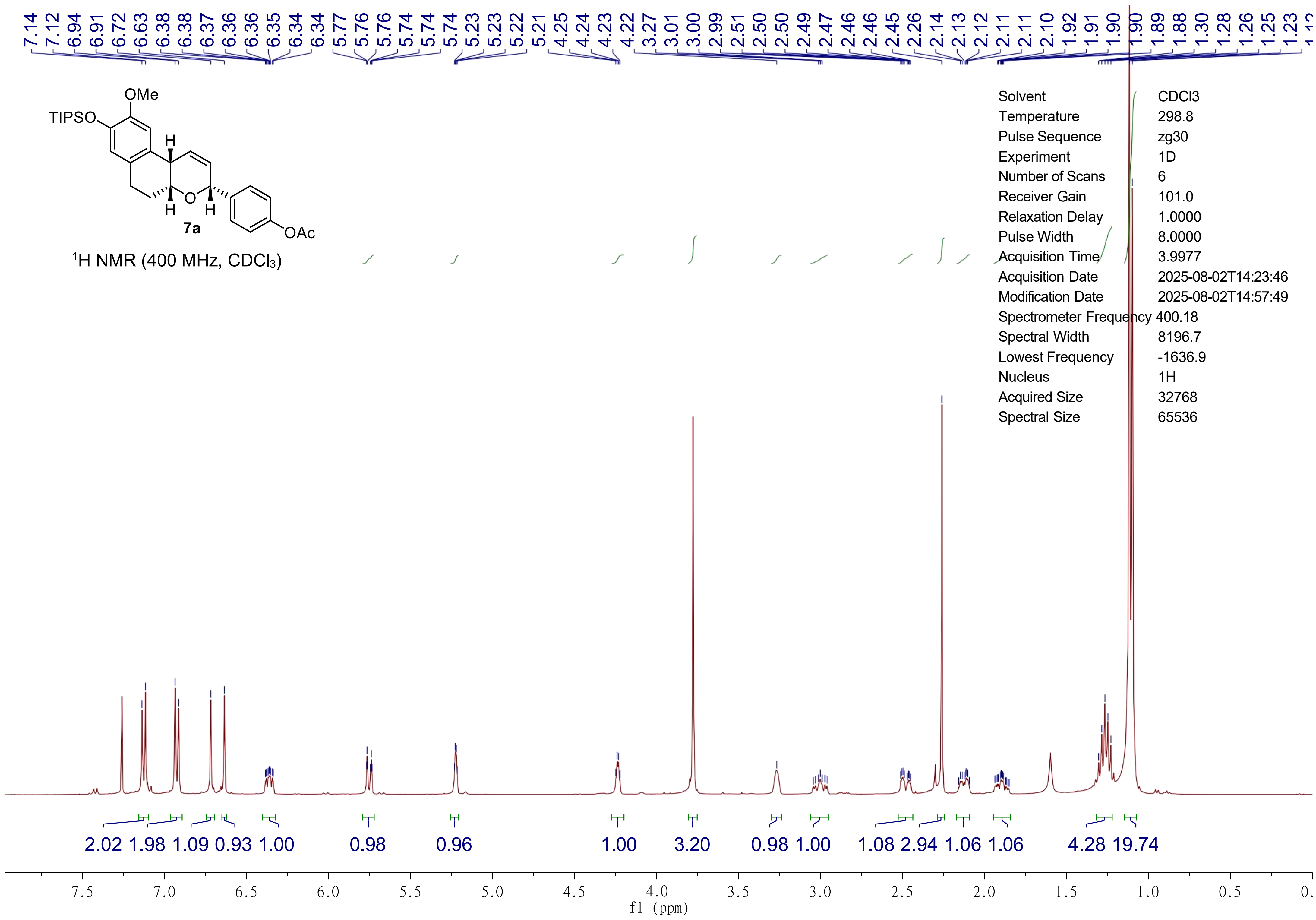

OMe
TIPSO
H
H
O
H
OAc
7a
¹H NMR (400 MHz, CDCl₃)
Solvent CDCl3
Temperature 298.8
Pulse Sequence zg30
Experiment 1D
Number of Scans 6
Receiver Gain 101.0
Relaxation Delay 1.0000
Pulse Width 8.0000
Acquisition Time 3.9977
Acquisition Date 2025-08-02T14:23:46
Modification Date 2025-08-02T14:57:49
Spectrometer Frequency 400.18
Spectral Width 8196.7
Lowest Frequency -1636.9
Nucleus 1H
Acquired Size 32768
Spectral Size 65536
7.14 7.12 6.94 6.91 6.72 6.63 6.38 6.38 6.37 6.36 6.36 6.35 6.34 6.34 5.77 5.76 5.76 5.74 5.74 5.74 5.23 5.23 5.22 5.21 4.25 4.24 4.23 4.22 3.27 3.01 3.00 2.99 2.51 2.50 2.50 2.49 2.47 2.46 2.46 2.45 2.26 2.14 2.13 2.12 2.11 2.11 2.10 1.92 1.91 1.90 1.90 1.89 1.88 1.30 1.28 1.26 1.25 1.23
2.02 1.98 1.09 0.93 1.00 0.98 0.96 1.00 3.20 0.98 1.00 1.08 2.94 1.06 1.06 4.28 19.74
7.5 7.0 6.5 6.0 5.5 5.0 4.5 4.0 3.5 3.0 2.5 2.0 1.5 1.0 0.5
f1 (ppm)

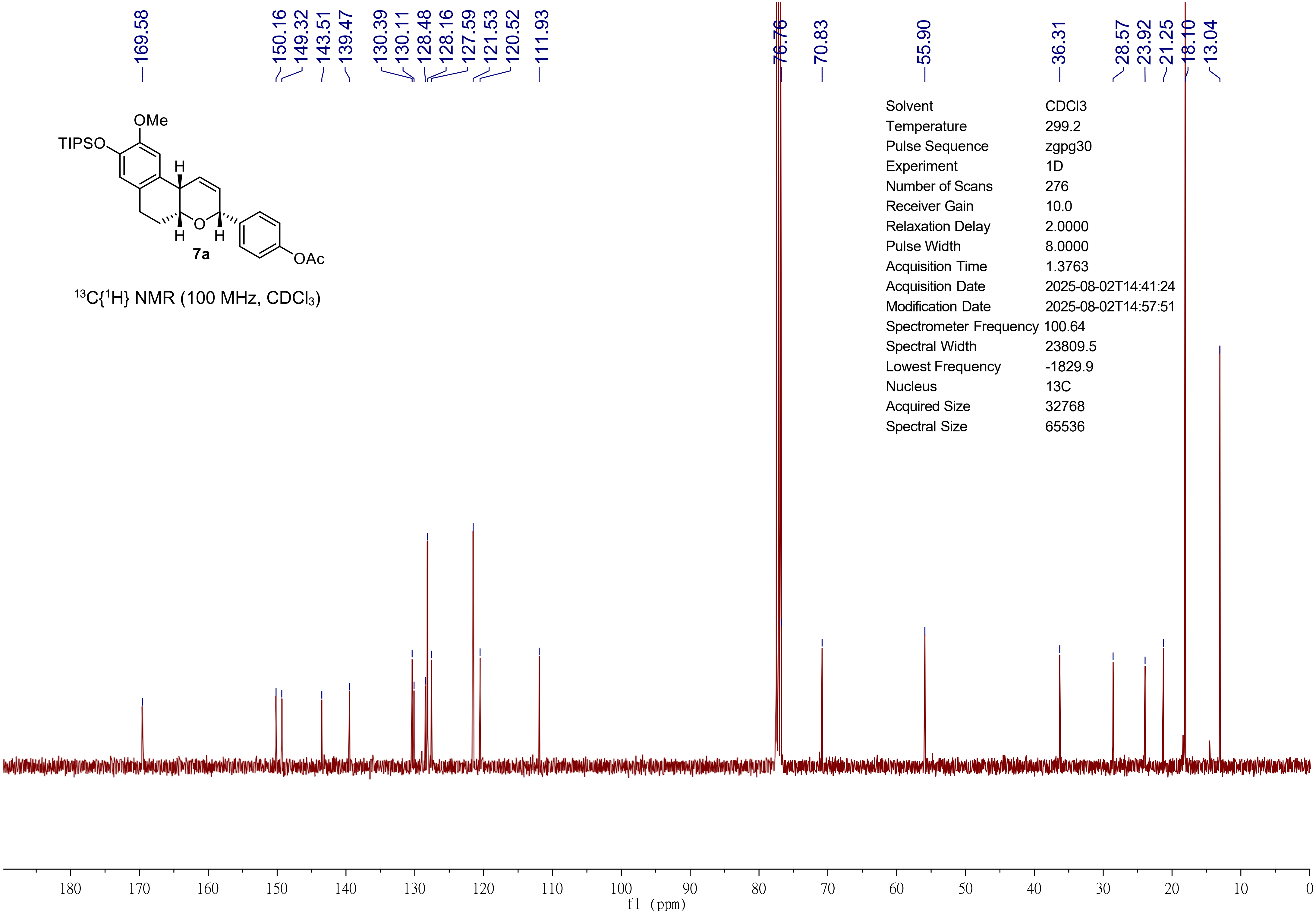


$^{13}C\{^{1}H\}$ NMR (100 MHz, $CDCl_3$)

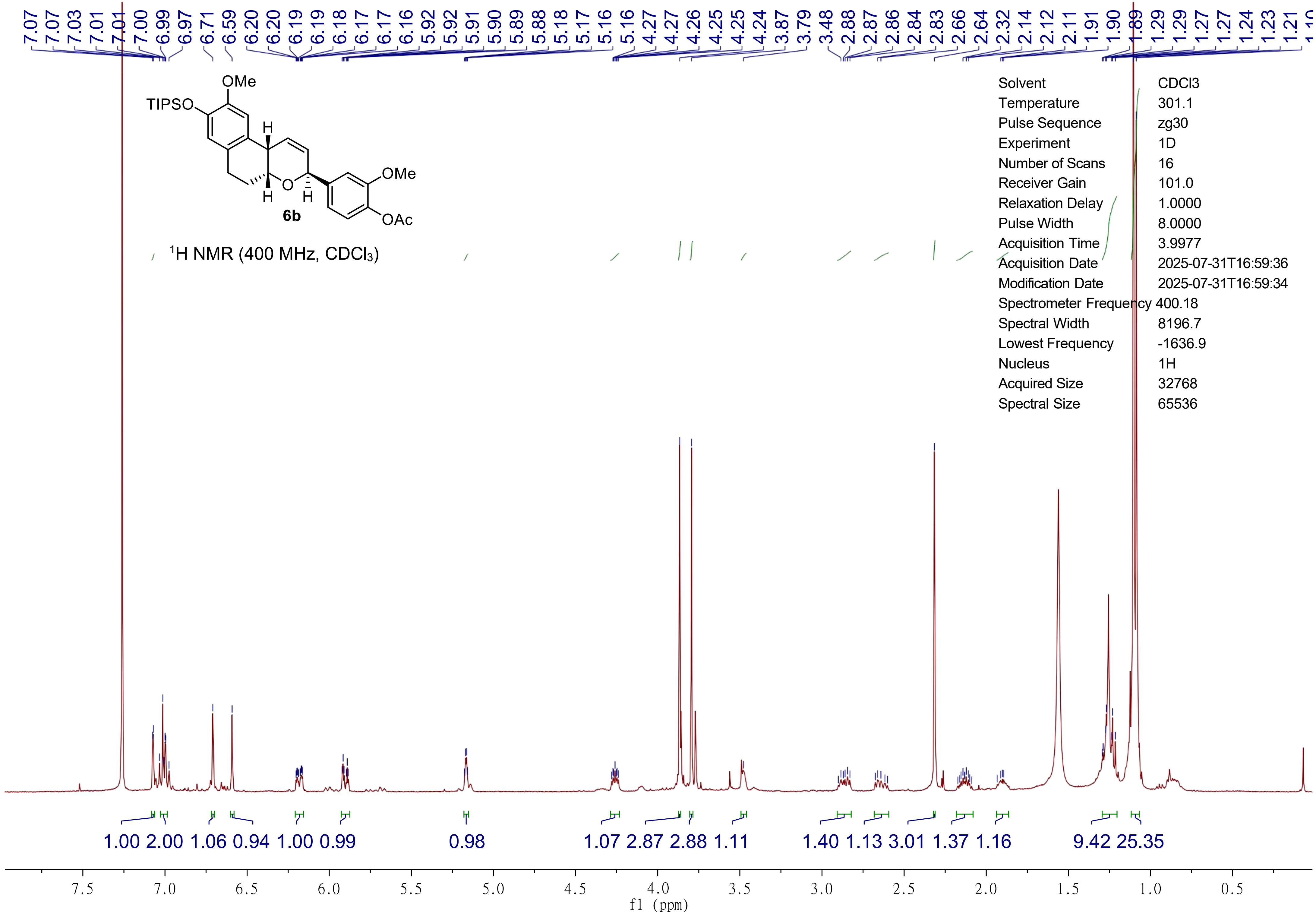

7.07 7.07 7.03 7.01 7.01 7.00 6.99 6.97 6.71 6.59 6.20 6.20 6.19 6.19 6.18 6.17 6.17 6.16 5.92 5.92 5.91 5.90 5.89 5.88 5.18 5.17 5.16 5.16 4.27 4.27 4.26 4.25 4.25 4.24 3.87 3.79 3.48 2.88 2.87 2.86 2.84 2.83 2.66 2.64 2.32 2.14 2.12 2.11 1.91 1.90 1.89 1.29 1.29 1.27 1.27 1.24 1.23 1.21
OMe
TIPSO
H
OMe
O
H
H
6b
OAc
$^1$H NMR (400 MHz, $CDCl_3$)
Solvent CDCl3
Temperature 301.1
Pulse Sequence zg30
Experiment 1D
Number of Scans 16
Receiver Gain 101.0
Relaxation Delay 1.0000
Pulse Width 8.0000
Acquisition Time 3.9977
Acquisition Date 2025-07-31T16:59:36
Modification Date 2025-07-31T16:59:34
Spectrometer Frequency 400.18
Spectral Width 8196.7
Lowest Frequency -1636.9
Nucleus 1H
Acquired Size 32768
Spectral Size 65536
1.00 2.00 1.06 0.94 1.00 0.99 0.98 1.07 2.87 2.88 1.11 1.40 1.13 3.01 1.37 1.16 9.42 25.35
7.5 7.0 6.5 6.0 5.5 5.0 4.5 4.0 3.5 3.0 2.5 2.0 1.5 1.0 0.5
f1 (ppm)

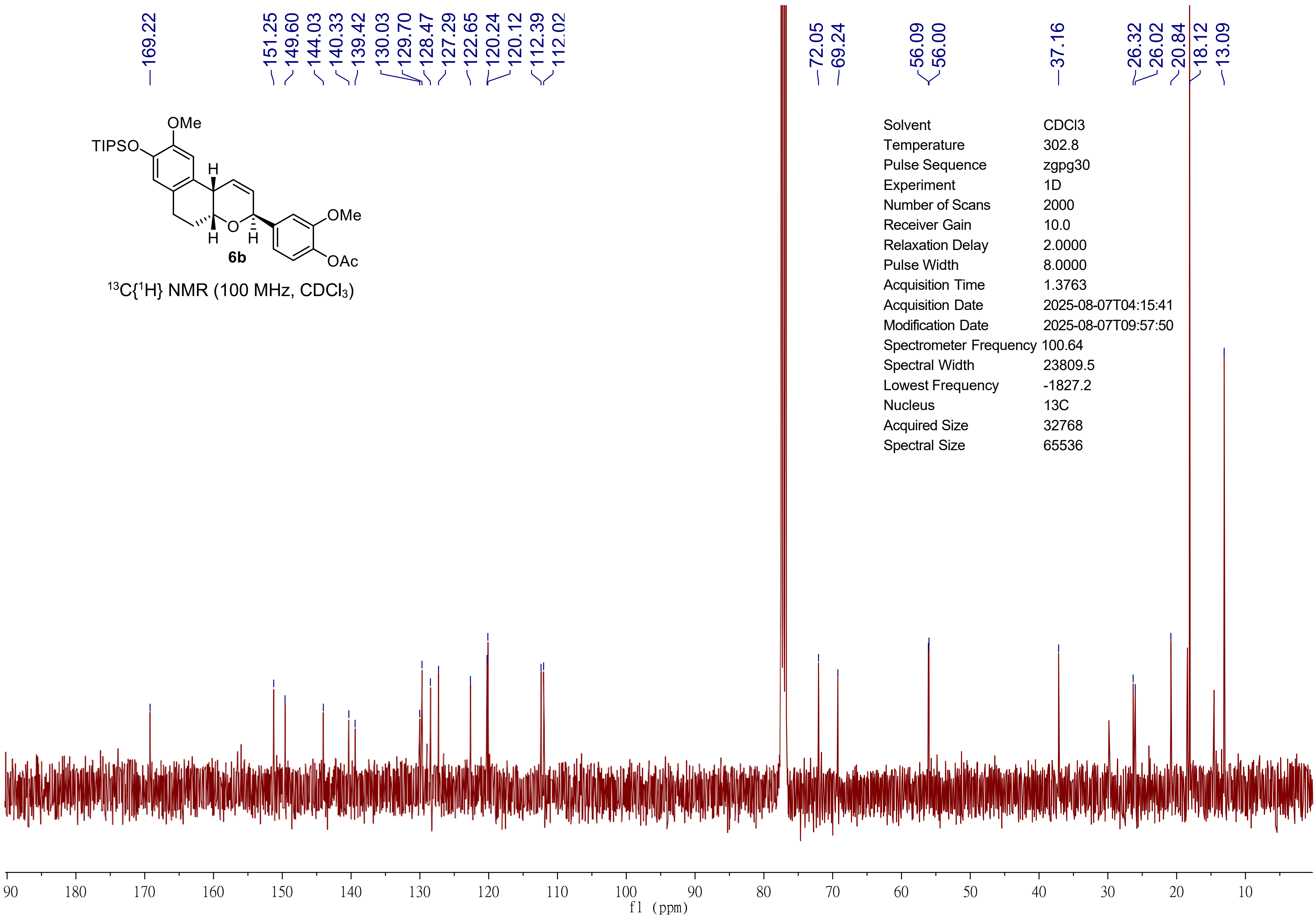
169.22
151.25
149.60
144.03
140.33
139.42
130.03
129.70
128.47
127.29
122.65
120.24
120.12
112.39
112.02
72.05
69.24
56.09
56.00
37.16
26.32
26.02
20.84
18.12
13.09
OMe
TIPSO
H
H
O
H
OMe
OAc
6b
$^{13}C\{^{1}H\}$ NMR (100 MHz, $CDCl_3$)
Solvent CDCl3
Temperature 302.8
Pulse Sequence zgpg30
Experiment 1D
Number of Scans 2000
Receiver Gain 10.0
Relaxation Delay 2.0000
Pulse Width 8.0000
Acquisition Time 1.3763
Acquisition Date 2025-08-07T04:15:41
Modification Date 2025-08-07T09:57:50
Spectrometer Frequency 100.64
Spectral Width 23809.5
Lowest Frequency -1827.2
Nucleus 13C
Acquired Size 32768
Spectral Size 65536
90 180 170 160 150 140 130 120 110 100 90 80 70 60 50 40 30 20 10
f1 (ppm)

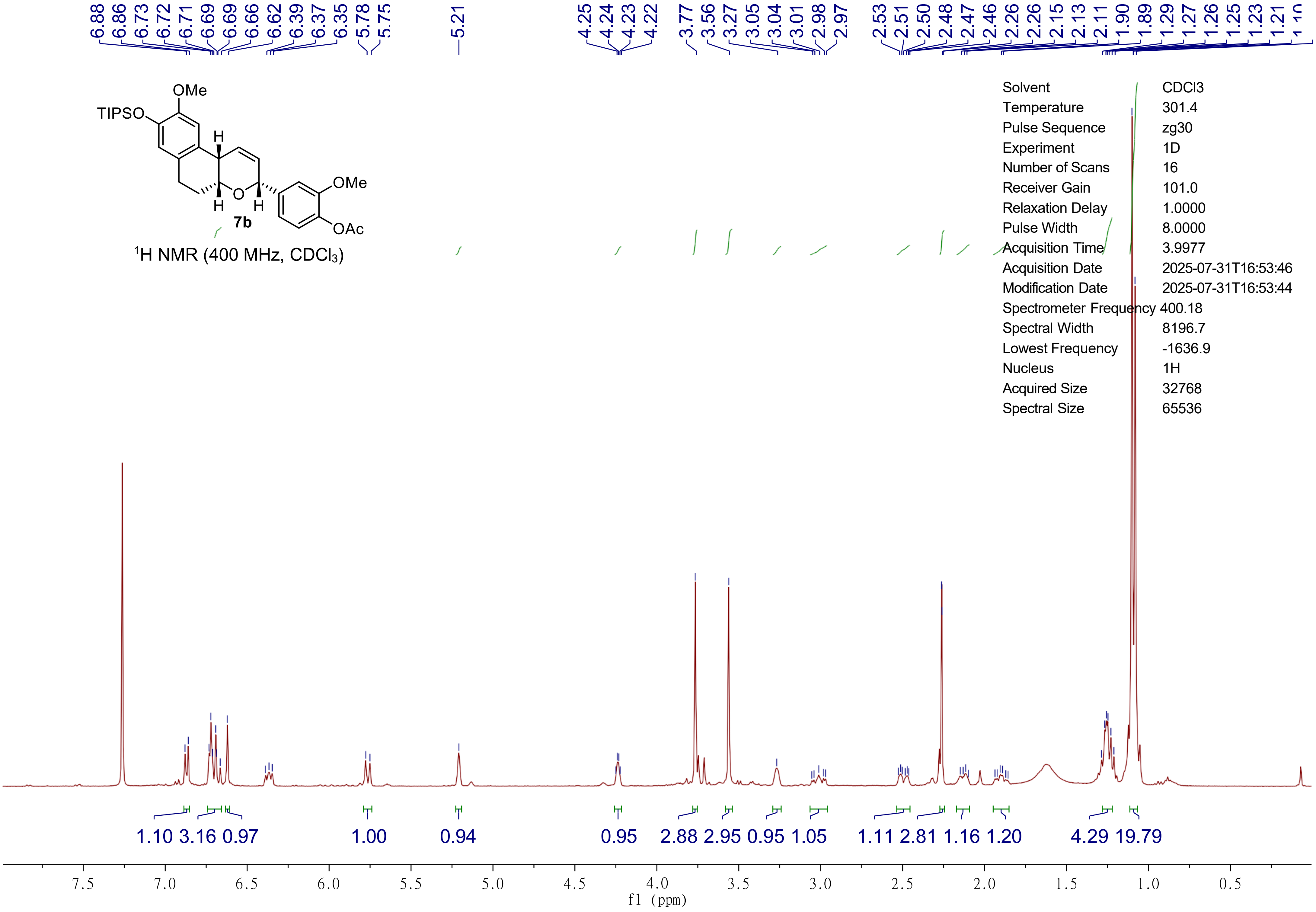

6.88 6.86 6.73 6.72 6.71 6.69 6.69 6.66 6.62 6.39 6.37 6.35 5.78 5.75
5.21
4.25 4.24 4.23 4.22 3.77 3.56 3.27 3.05 3.04 3.01 2.98 2.97
2.53 2.51 2.50 2.48 2.47 2.46 2.26 2.26 2.15 2.13 2.11 1.90 1.89 1.29 1.27 1.26 1.25 1.23 1.21
OMe
TIPSO
H
OMe
H
O
H
OAc
7b
$^1$H NMR (400 MHz, $CDCl_3$)
Solvent CDCl3
Temperature 301.4
Pulse Sequence zg30
Experiment 1D
Number of Scans 16
Receiver Gain 101.0
Relaxation Delay 1.0000
Pulse Width 8.0000
Acquisition Time 3.9977
Acquisition Date 2025-07-31T16:53:46
Modification Date 2025-07-31T16:53:44
Spectrometer Frequency 400.18
Spectral Width 8196.7
Lowest Frequency -1636.9
Nucleus 1H
Acquired Size 32768
Spectral Size 65536
1.10 3.16 0.97 1.00 0.94 0.95 2.88 2.95 0.95 1.05 1.11 2.81 1.16 1.20 4.29 19.79
7.5 7.0 6.5 6.0 5.5 5.0 4.5 4.0 3.5 3.0 2.5 2.0 1.5 1.0 0.5
f1 (ppm)

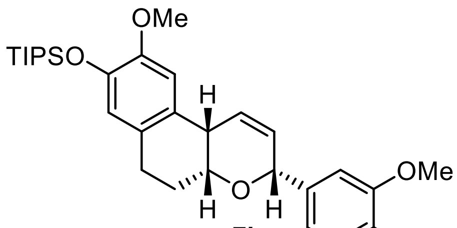


**7b**

$^{13}C\{^{1}H\}$ NMR (100 MHz, $CDCl_3$)

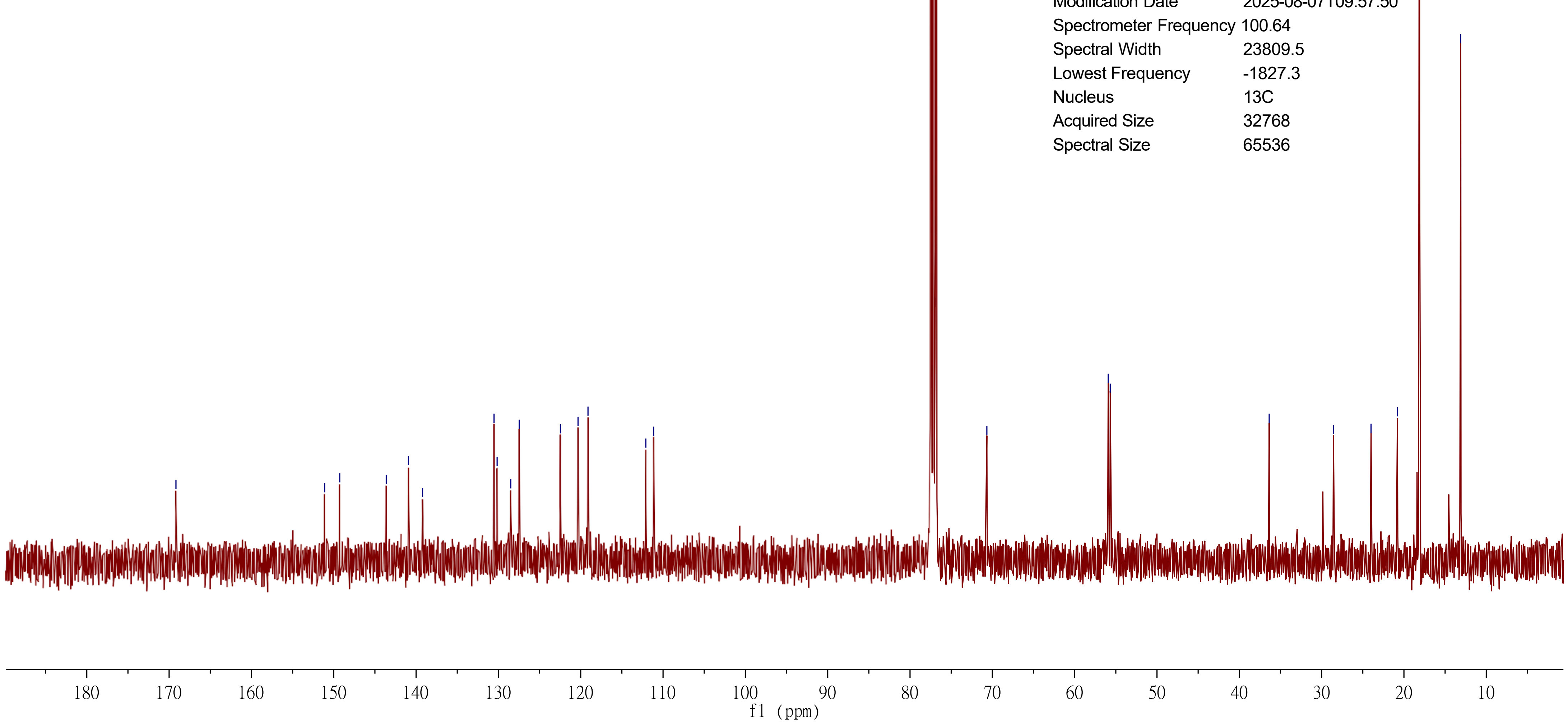


| | |
|---|---|
| Solvent | CDCl3 |
| Temperature | 302.9 |
| Pulse Sequence | zgpg30 |
| Experiment | 1D |
| Number of Scans | 2000 |
| Receiver Gain | 10.0 |
| Relaxation Delay | 2.0000 |
| Pulse Width | 8.0000 |
| Acquisition Time | 1.3763 |
| Acquisition Date | 2025-08-07T02:12:19 |
| Modification Date | 2025-08-07T09:57:50 |
| Spectrometer Frequency | 100.64 |
| Spectral Width | 23809.5 |
| Lowest Frequency | -1827.3 |
| Nucleus | 13C |
| Acquired Size | 32768 |
| Spectral Size | 65536 |

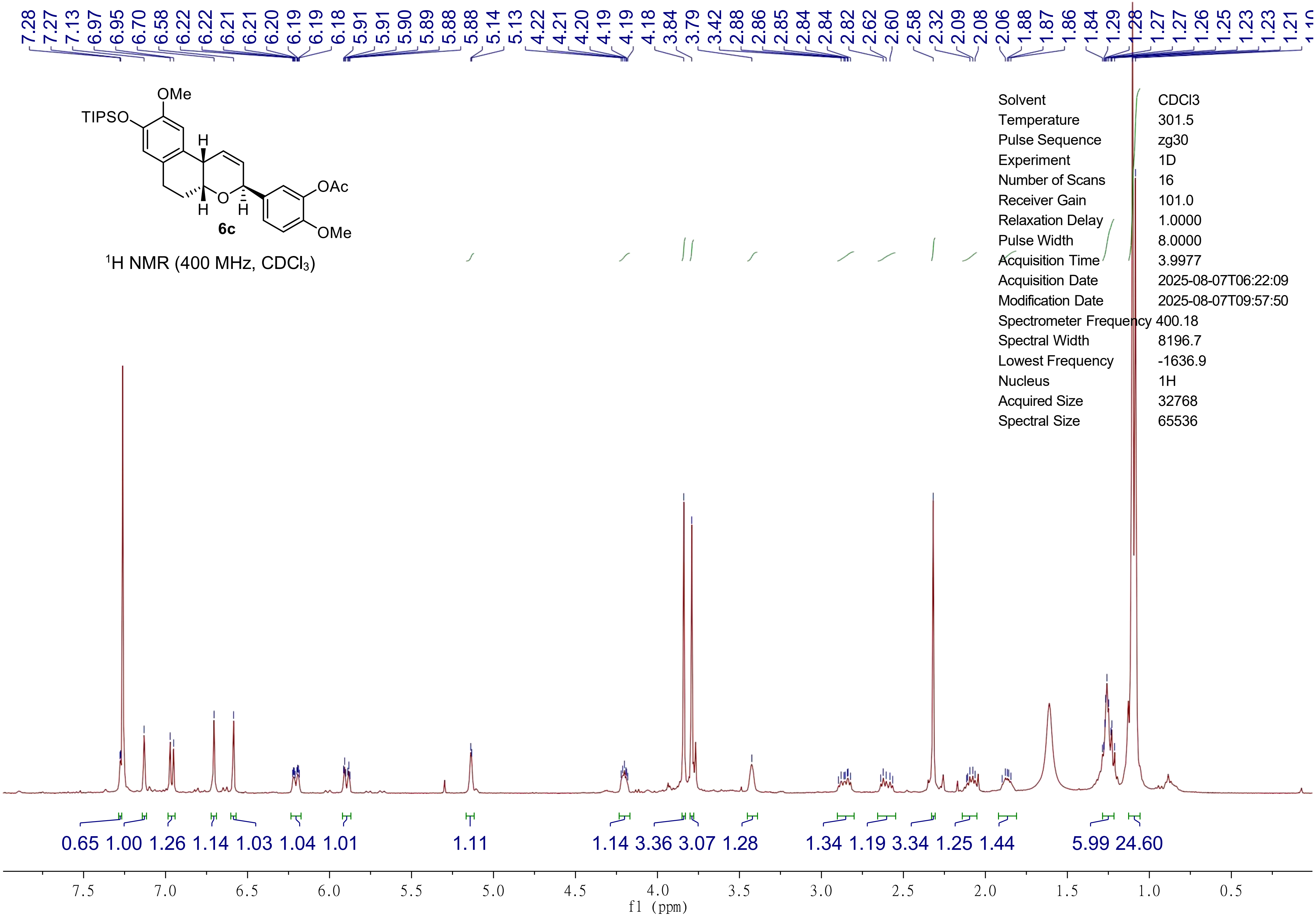
7.28 7.27 7.13 6.97 6.95 6.70 6.58 6.22 6.22 6.21 6.21 6.20 6.19 6.19 6.18 5.91 5.91 5.90 5.89 5.88 5.88 5.14 5.13 4.22 4.21 4.20 4.19 4.19 4.18 3.84 3.79 3.42 2.88 2.86 2.85 2.84 2.84 2.82 2.62 2.60 2.58 2.32 2.09 2.08 2.06 1.88 1.87 1.86 1.84 1.29 1.28 1.27 1.27 1.26 1.25 1.23 1.23 1.21
OMe
TIPSO
H
OAc
O
H
H
OMe
6c
1H NMR (400 MHz, CDCl3)
Solvent CDCl3
Temperature 301.5
Pulse Sequence zg30
Experiment 1D
Number of Scans 16
Receiver Gain 101.0
Relaxation Delay 1.0000
Pulse Width 8.0000
Acquisition Time 3.9977
Acquisition Date 2025-08-07T06:22:09
Modification Date 2025-08-07T09:57:50
Spectrometer Frequency 400.18
Spectral Width 8196.7
Lowest Frequency -1636.9
Nucleus 1H
Acquired Size 32768
Spectral Size 65536
0.65 1.00 1.26 1.14 1.03 1.04 1.01 1.11 1.14 3.36 3.07 1.28 1.34 1.19 3.34 1.25 1.44 5.99 24.60
7.5 7.0 6.5 6.0 5.5 5.0 4.5 4.0 3.5 3.0 2.5 2.0 1.5 1.0 0.5
f1 (ppm)

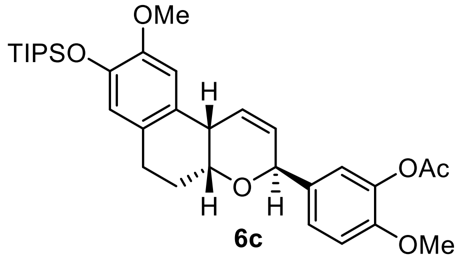


**6c**

$^{13}C\{^{1}H\}$ NMR (100 MHz, $CDCl_3$)

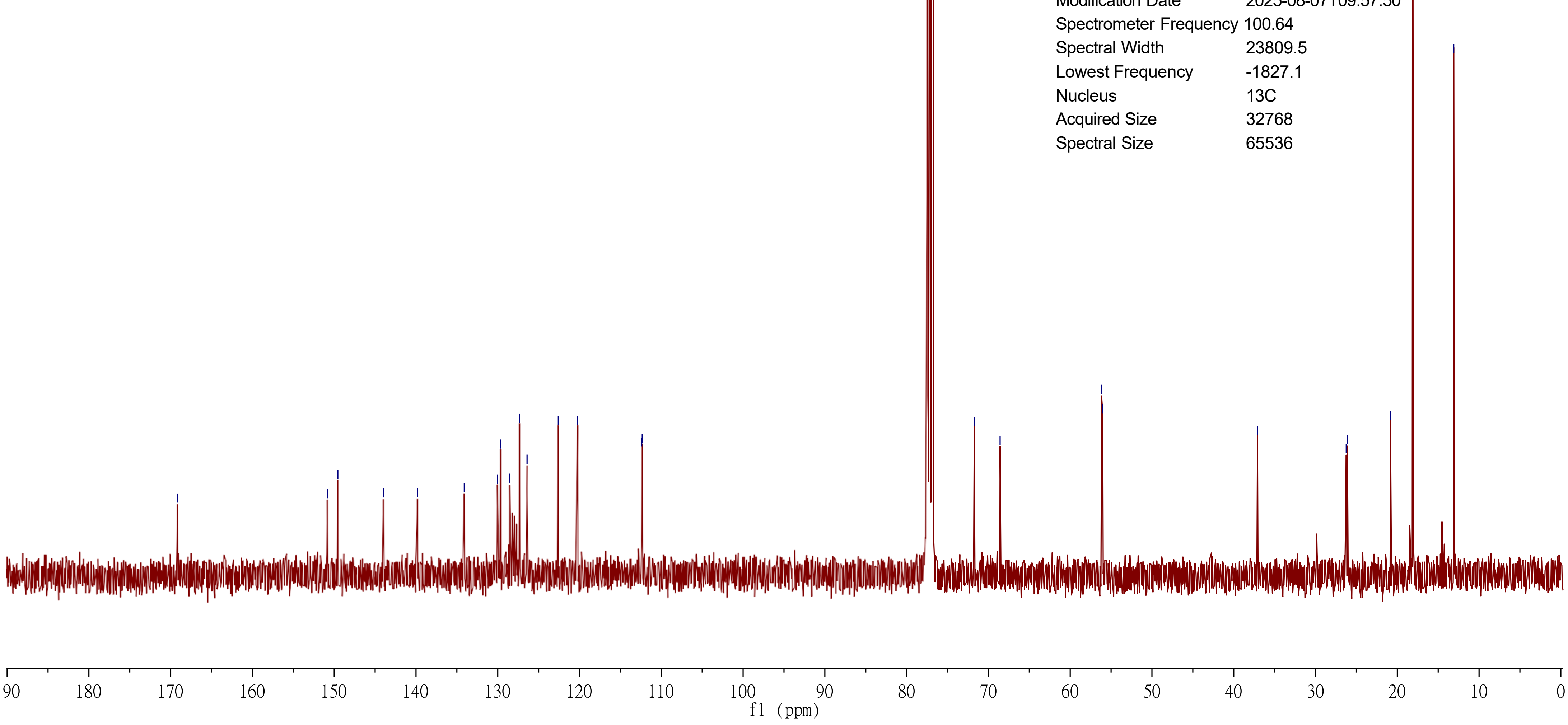

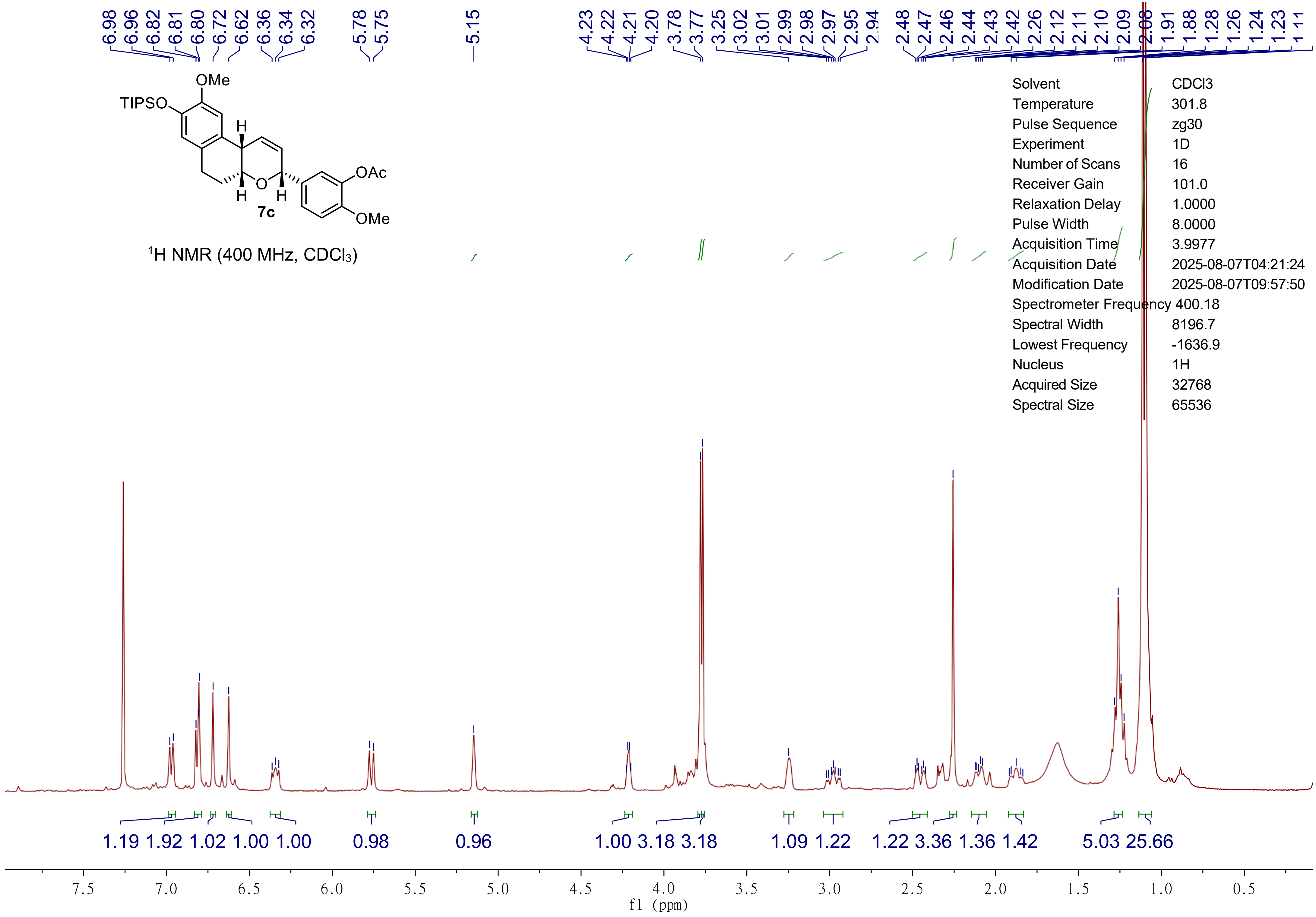
6.98 6.96 6.82 6.81 6.80 6.72 6.62 6.36 6.34 6.32 5.78 5.75 5.15 4.23 4.22 4.21 4.20 3.78 3.77 3.25 3.02 3.01 2.99 2.98 2.97 2.95 2.94 2.48 2.47 2.46 2.44 2.43 2.42 2.26 2.12 2.11 2.10 2.09 2.08 1.91 1.88 1.28 1.26 1.24 1.23 1.11
OMe
TIPSO
H
OAc
O
H
H
7c
OMe
$^{1}$H NMR (400 MHz, CDCl$_3$)
Solvent CDCl3
Temperature 301.8
Pulse Sequence zg30
Experiment 1D
Number of Scans 16
Receiver Gain 101.0
Relaxation Delay 1.0000
Pulse Width 8.0000
Acquisition Time 3.9977
Acquisition Date 2025-08-07T04:21:24
Modification Date 2025-08-07T09:57:50
Spectrometer Frequency 400.18
Spectral Width 8196.7
Lowest Frequency -1636.9
Nucleus 1H
Acquired Size 32768
Spectral Size 65536
1.19 1.92 1.02 1.00 1.00 0.98 0.96 1.00 3.18 3.18 1.09 1.22 1.22 3.36 1.36 1.42 5.03 25.66
7.5 7.0 6.5 6.0 5.5 5.0 4.5 4.0 3.5 3.0 2.5 2.0 1.5 1.0 0.5
f1 (ppm)

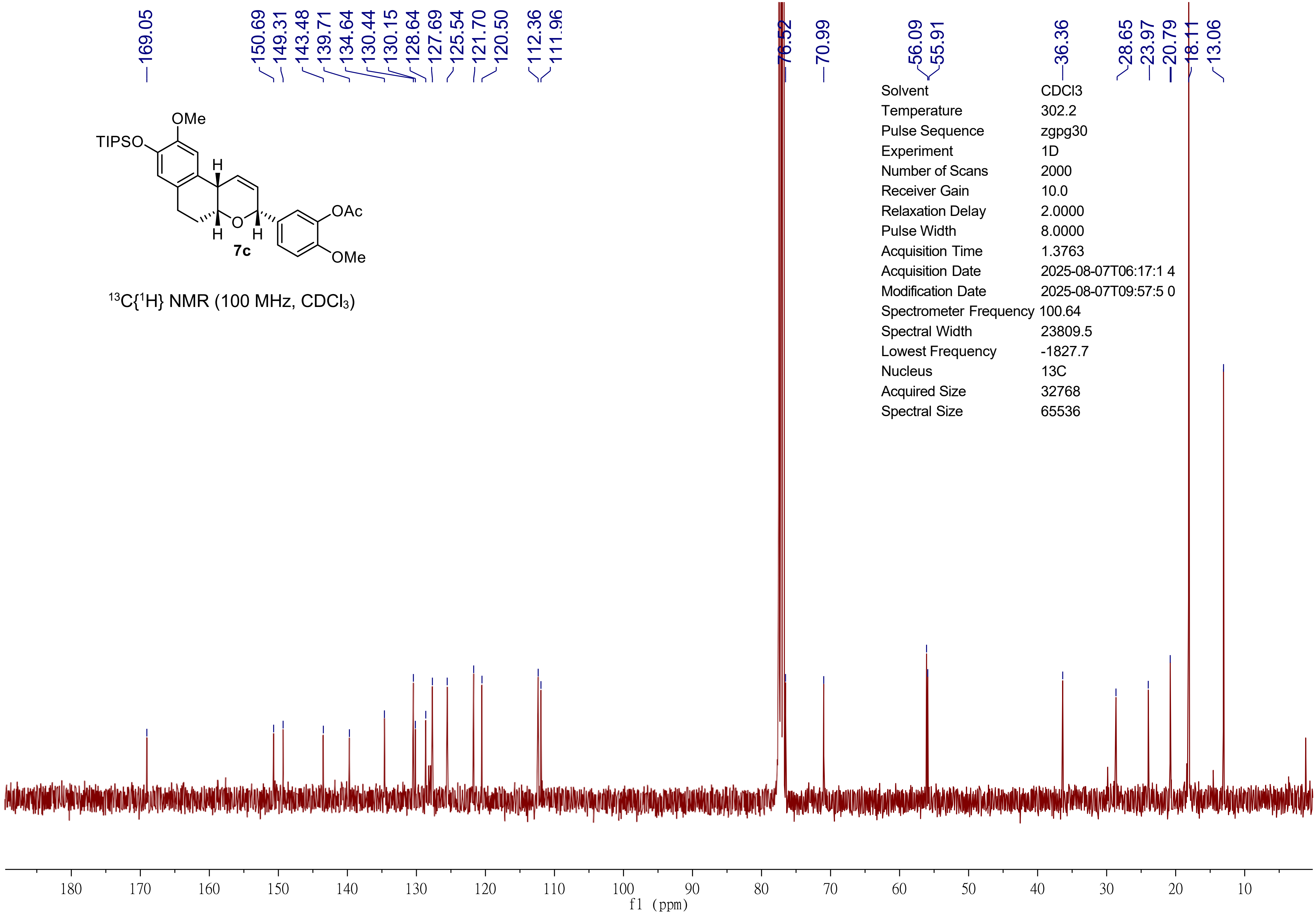
169.05
150.69
149.31
143.48
139.71
134.64
130.44
130.15
128.64
127.69
125.54
121.70
120.50
112.36
111.96
76.52
70.99
56.09
55.91
36.36
28.65
23.97
20.79
18.11
13.06
OMe
TIPSO
H
OAc
O
H
H
7c
OMe
$^{13}C\{^{1}H\}$ NMR (100 MHz, $CDCl_3$)
Solvent CDCl3
Temperature 302.2
Pulse Sequence zgpg30
Experiment 1D
Number of Scans 2000
Receiver Gain 10.0
Relaxation Delay 2.0000
Pulse Width 8.0000
Acquisition Time 1.3763
Acquisition Date 2025-08-07T06:17:1 4
Modification Date 2025-08-07T09:57:5 0
Spectrometer Frequency 100.64
Spectral Width 23809.5
Lowest Frequency -1827.7
Nucleus 13C
Acquired Size 32768
Spectral Size 65536
180
170
160
150
140
130
120
110
100
90
80
70
60
50
40
30
20
10
f1 (ppm)

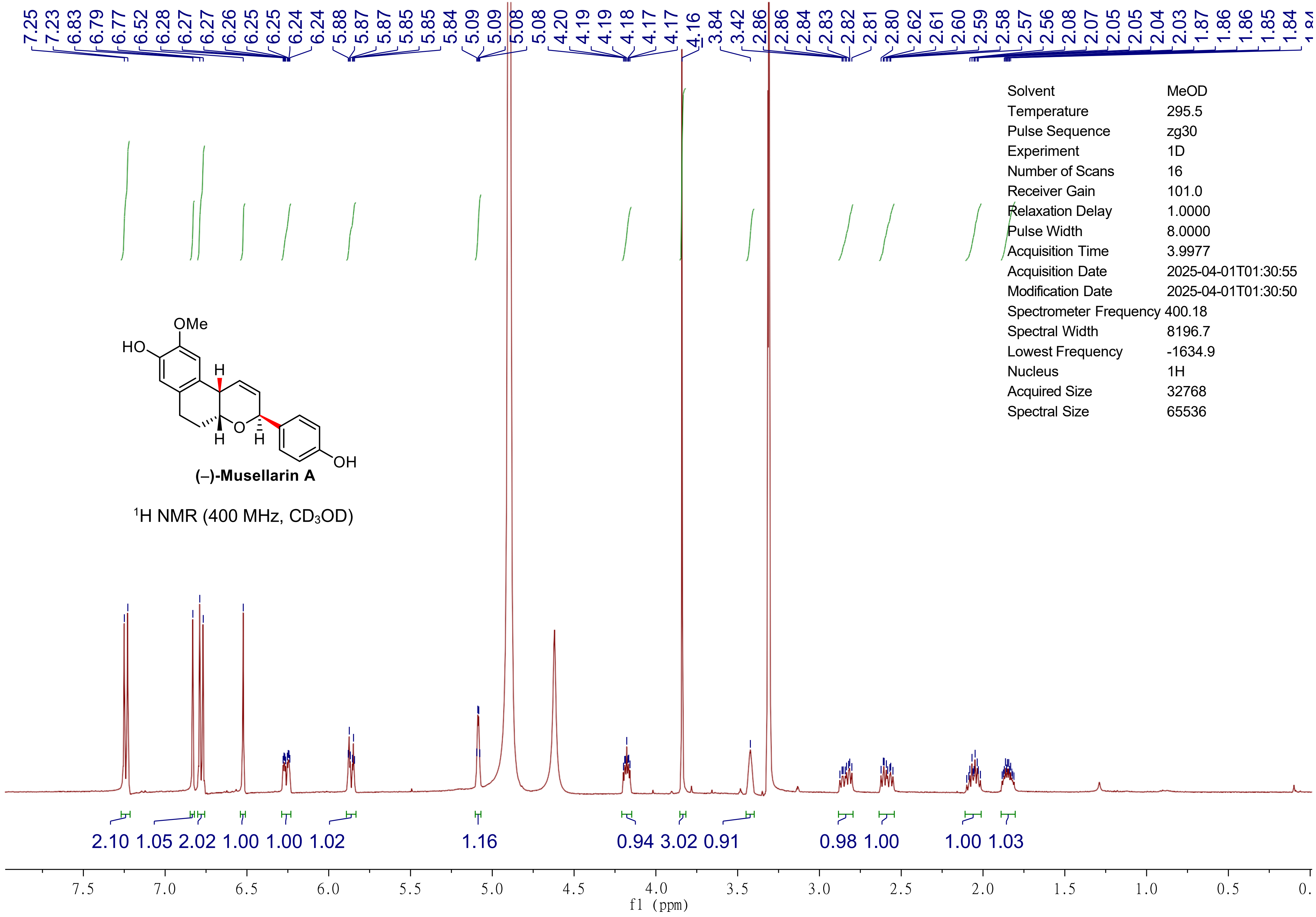
7.25 7.23 6.83 6.79 6.77 6.52 6.28 6.27 6.27 6.26 6.25 6.25 6.24 6.24 5.88 5.87 5.87 5.85 5.85 5.84 5.09 5.09 5.08 5.08 4.20 4.19 4.19 4.18 4.17 4.17 4.16 3.84 3.42 2.86 2.86 2.84 2.83 2.82 2.81 2.80 2.62 2.61 2.60 2.59 2.58 2.57 2.56 2.08 2.07 2.05 2.05 2.04 2.03 1.87 1.86 1.86 1.85 1.84
Solvent MeOD
Temperature 295.5
Pulse Sequence zg30
Experiment 1D
Number of Scans 16
Receiver Gain 101.0
Relaxation Delay 1.0000
Pulse Width 8.0000
Acquisition Time 3.9977
Acquisition Date 2025-04-01T01:30:55
Modification Date 2025-04-01T01:30:50
Spectrometer Frequency 400.18
Spectral Width 8196.7
Lowest Frequency -1634.9
Nucleus 1H
Acquired Size 32768
Spectral Size 65536
OMe
HO
H
O
H
H
OH
(–)-Musellarin A
1H NMR (400 MHz, CD3OD)
2.10 1.05 2.02 1.00 1.00 1.02 1.16 0.94 3.02 0.91 0.98 1.00 1.00 1.03
7.5 7.0 6.5 6.0 5.5 5.0 4.5 4.0 3.5 3.0 2.5 2.0 1.5 1.0 0.5 0.
f1 (ppm)

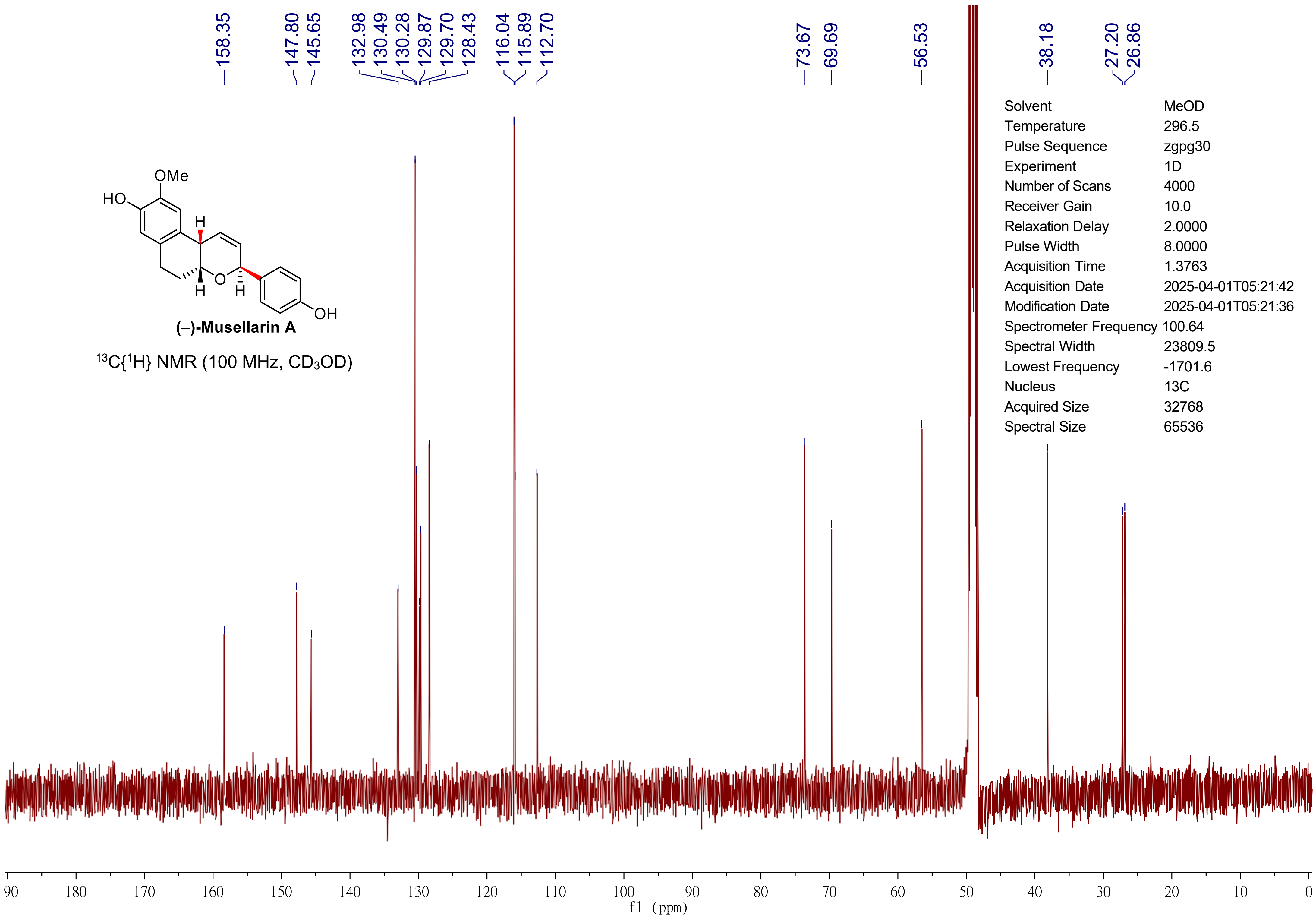

158.35
147.80
145.65
132.98
130.49
130.28
129.87
129.70
128.43
116.04
115.89
112.70
73.67
69.69
56.53
38.18
27.20
26.86
OMe
HO
H
H
O
H
OH
(–)-Musellarin A
13C{1H} NMR (100 MHz, CD3OD)
Solvent MeOD
Temperature 296.5
Pulse Sequence zgpg30
Experiment 1D
Number of Scans 4000
Receiver Gain 10.0
Relaxation Delay 2.0000
Pulse Width 8.0000
Acquisition Time 1.3763
Acquisition Date 2025-04-01T05:21:42
Modification Date 2025-04-01T05:21:36
Spectrometer Frequency 100.64
Spectral Width 23809.5
Lowest Frequency -1701.6
Nucleus 13C
Acquired Size 32768
Spectral Size 65536
190 180 170 160 150 140 130 120 110 100 90 80 70 60 50 40 30 20 10 0
f1 (ppm)

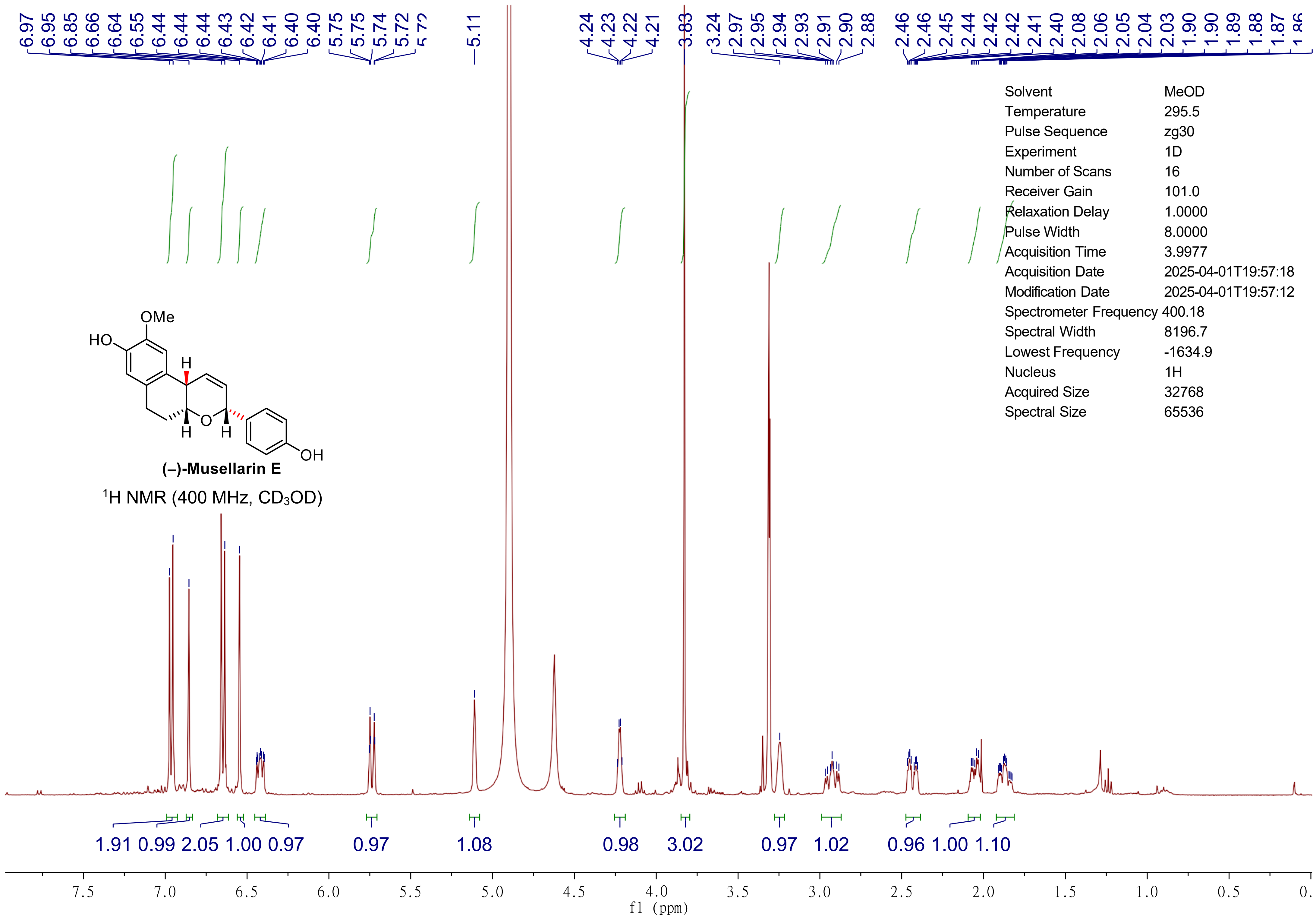

6.97 6.95 6.85 6.66 6.64 6.55 6.44 6.44 6.44 6.43 6.42 6.41 6.40 6.40 5.75 5.75 5.74 5.72
5.11
4.24 4.23 4.22 4.21 3.83 3.24 2.97 2.95 2.94 2.93 2.91 2.90 2.88
2.46 2.46 2.45 2.44 2.42 2.42 2.41 2.40 2.08 2.06 2.05 2.04 2.03 1.90 1.90 1.89 1.88 1.87
Solvent MeOD
Temperature 295.5
Pulse Sequence zg30
Experiment 1D
Number of Scans 16
Receiver Gain 101.0
Relaxation Delay 1.0000
Pulse Width 8.0000
Acquisition Time 3.9977
Acquisition Date 2025-04-01T19:57:18
Modification Date 2025-04-01T19:57:12
Spectrometer Frequency 400.18
Spectral Width 8196.7
Lowest Frequency -1634.9
Nucleus 1H
Acquired Size 32768
Spectral Size 65536
OMe
HO
H
O
H
H
OH
(–)-Musellarin E
1H NMR (400 MHz, CD3OD)
1.91 0.99 2.05 1.00 0.97 0.97 1.08 0.98 3.02 0.97 1.02 0.96 1.00 1.10
7.5 7.0 6.5 6.0 5.5 5.0 4.5 4.0 3.5 3.0 2.5 2.0 1.5 1.0 0.5 0.
f1 (ppm)

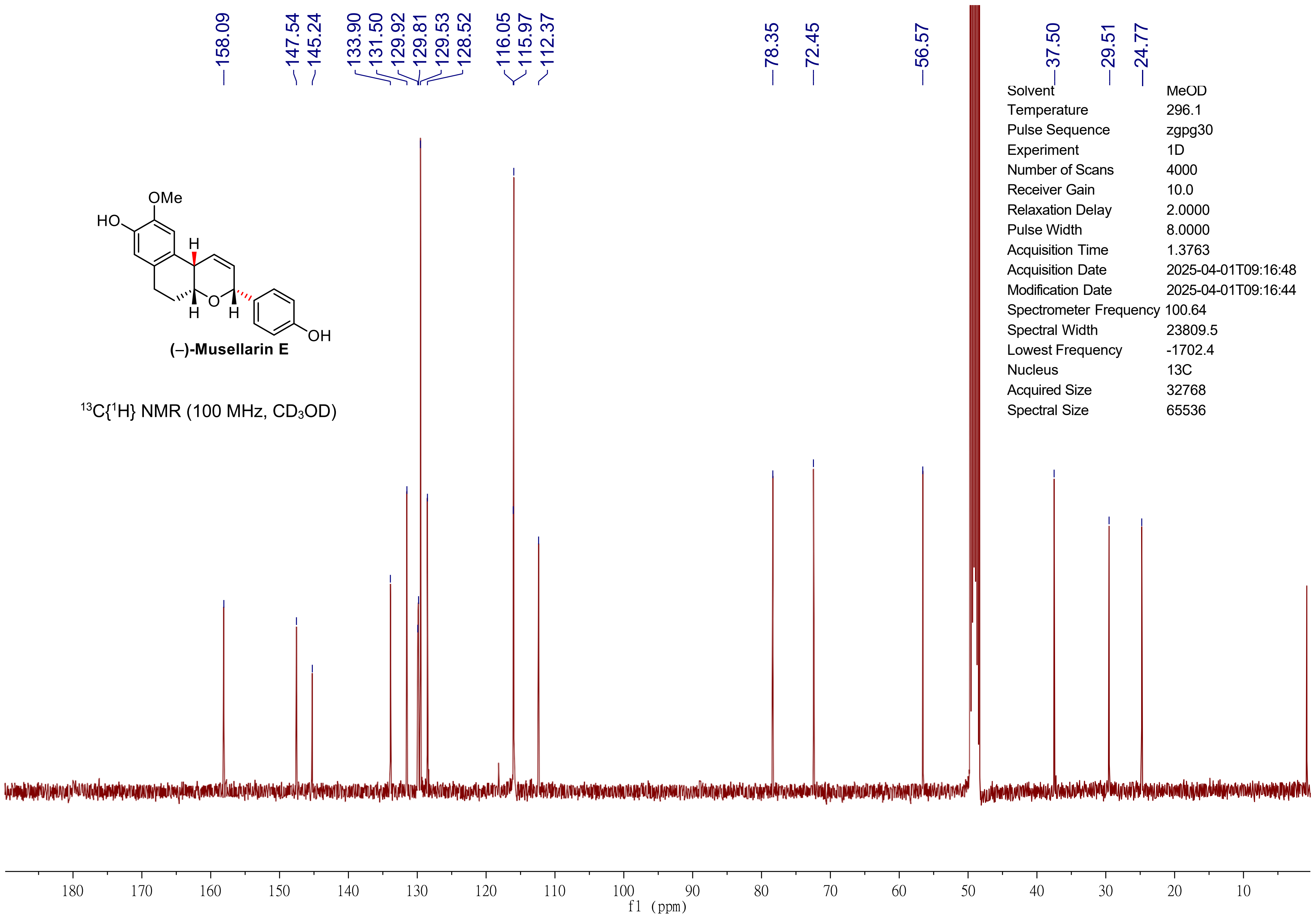
158.09
147.54
145.24
133.90
131.50
129.92
129.81
129.53
128.52
116.05
115.97
112.37
78.35
72.45
56.57
37.50
29.51
24.77
OMe
HO
H
O
H
H
OH
(–)-Musellarin E
13C{1H} NMR (100 MHz, CD3OD)
Solvent MeOD
Temperature 296.1
Pulse Sequence zgpg30
Experiment 1D
Number of Scans 4000
Receiver Gain 10.0
Relaxation Delay 2.0000
Pulse Width 8.0000
Acquisition Time 1.3763
Acquisition Date 2025-04-01T09:16:48
Modification Date 2025-04-01T09:16:44
Spectrometer Frequency 100.64
Spectral Width 23809.5
Lowest Frequency -1702.4
Nucleus 13C
Acquired Size 32768
Spectral Size 65536
180
170
160
150
140
130
120
110
100
90
80
70
60
50
40
30
20
10
f1 (ppm)

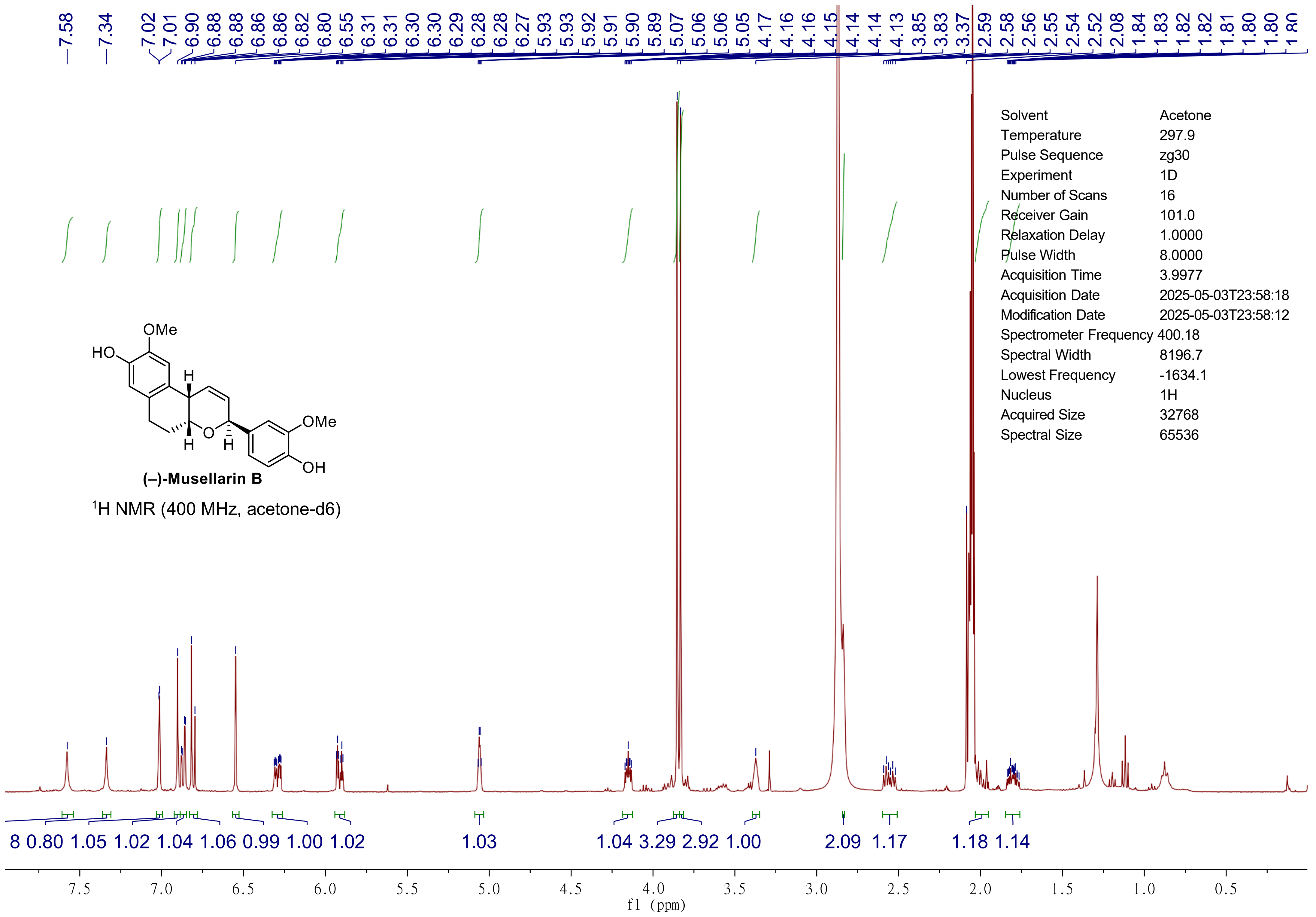


(–)-Musellarin B

$^1$H NMR (400 MHz, acetone-d6)

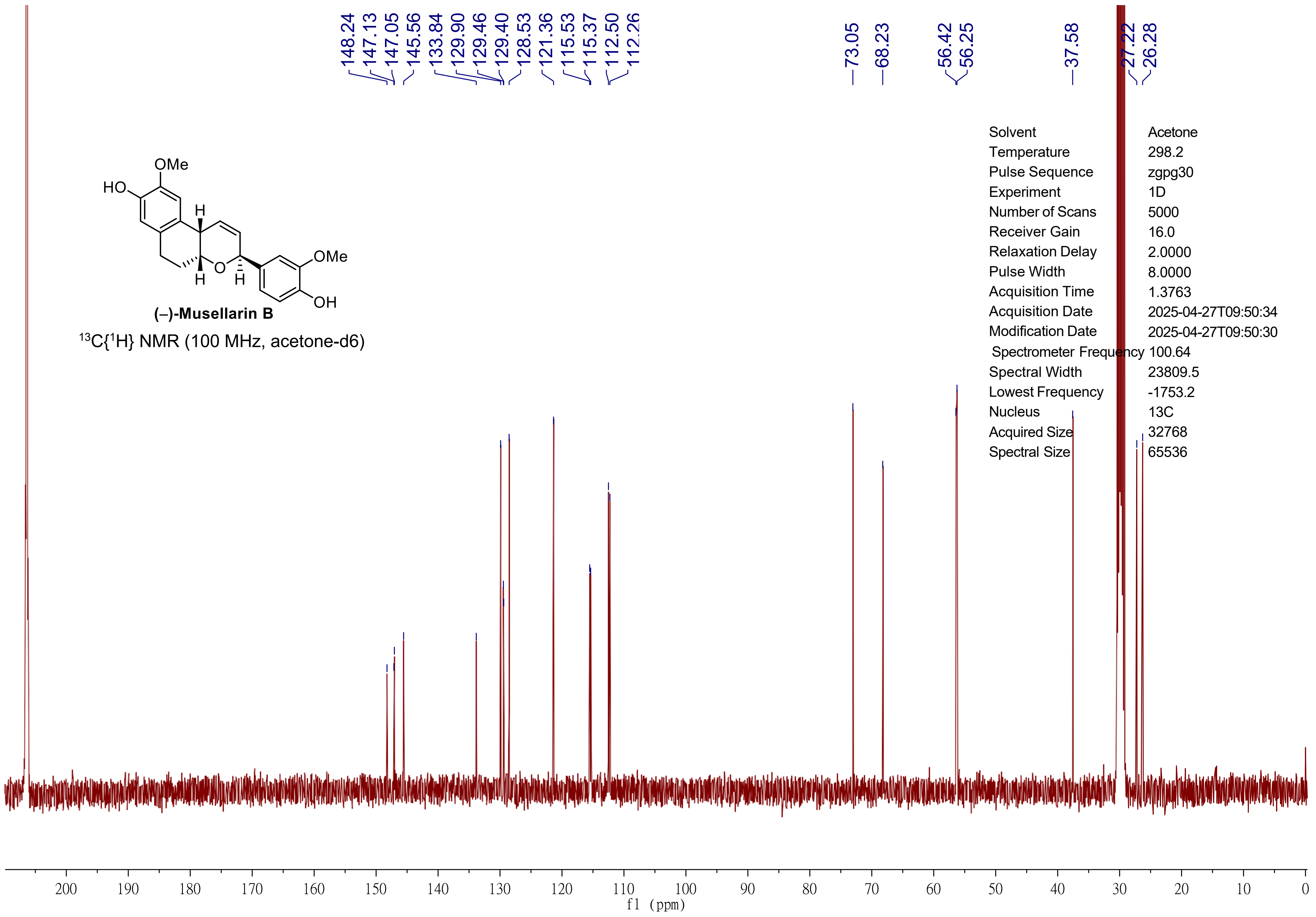


**(–)-Musellarin B**

$^{13}C\{^{1}H\}$ NMR (100 MHz, acetone-d6)

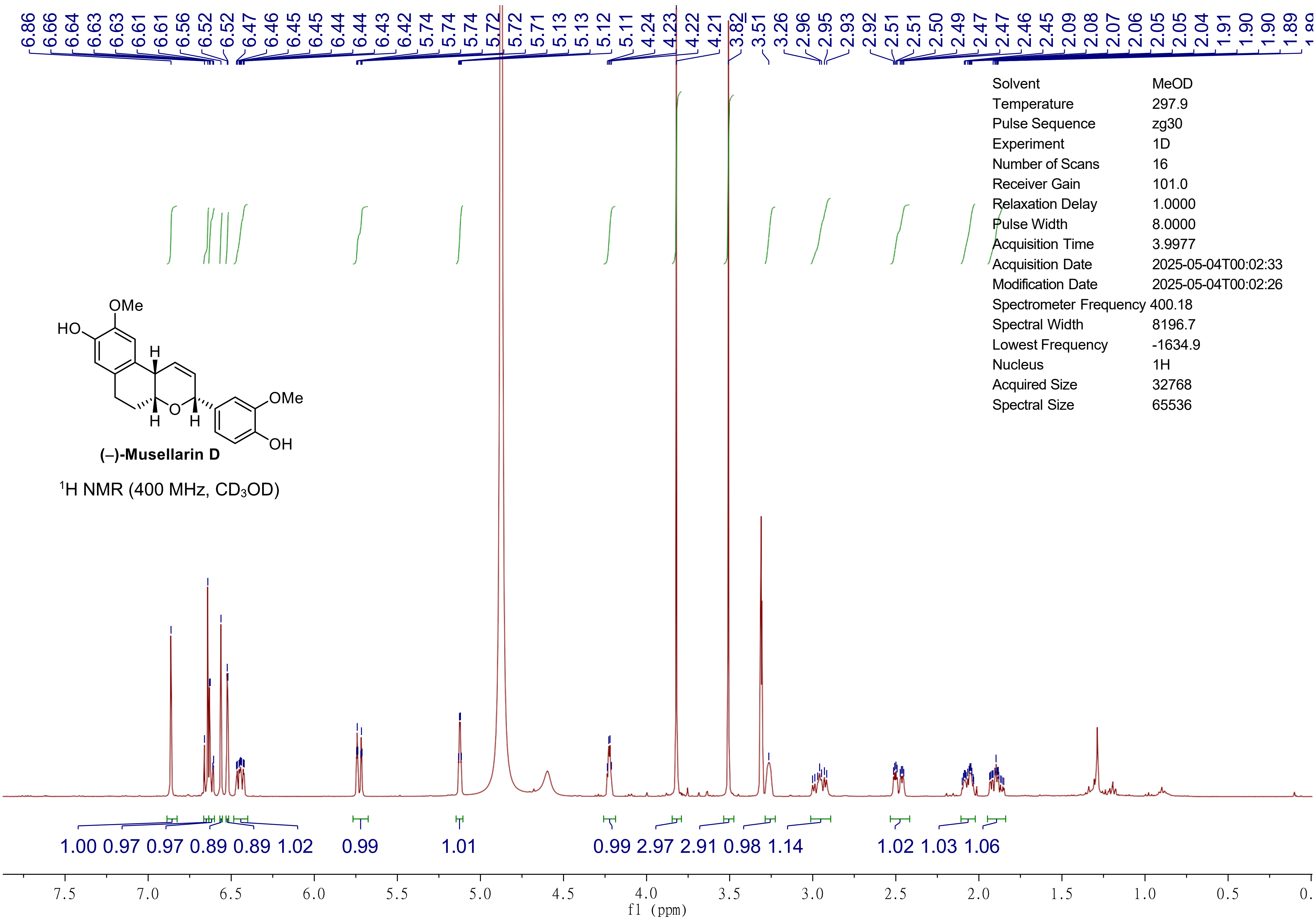

6.86 6.66 6.64 6.63 6.63 6.61 6.61 6.56 6.52 6.52 6.47 6.46 6.45 6.45 6.44 6.44 6.43 6.42 5.74 5.74 5.74 5.72 5.72 5.71 5.13 5.13 5.12 5.11 4.24 4.23 4.22 4.21 3.82 3.51 3.26 2.96 2.95 2.93 2.92 2.51 2.51 2.50 2.49 2.47 2.47 2.46 2.45 2.09 2.08 2.07 2.06 2.05 2.05 2.04 1.91 1.90 1.90 1.89
Solvent MeOD
Temperature 297.9
Pulse Sequence zg30
Experiment 1D
Number of Scans 16
Receiver Gain 101.0
Relaxation Delay 1.0000
Pulse Width 8.0000
Acquisition Time 3.9977
Acquisition Date 2025-05-04T00:02:33
Modification Date 2025-05-04T00:02:26
Spectrometer Frequency 400.18
Spectral Width 8196.7
Lowest Frequency -1634.9
Nucleus 1H
Acquired Size 32768
Spectral Size 65536
OMe
HO
H
H
O
H
OMe
OH
(–)-Musellarin D
1H NMR (400 MHz, CD3OD)
1.00 0.97 0.97 0.89 0.89 1.02 0.99 1.01 0.99 2.97 2.91 0.98 1.14 1.02 1.03 1.06
7.5 7.0 6.5 6.0 5.5 5.0 4.5 4.0 3.5 3.0 2.5 2.0 1.5 1.0 0.5 0.
f1 (ppm)

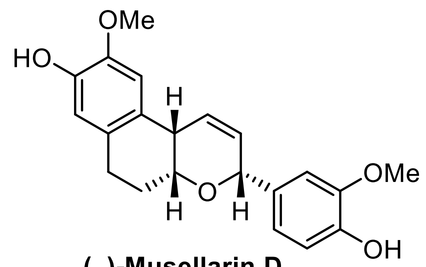


**(–)-Musellarin D**

$^{13}C\{^1H\}$ NMR (100 MHz, $CD_3OD$)

| | |
|---|---|
| Solvent | MeOD |
| Temperature | 298.5 |
| Pulse Sequence | zgpg30 |
| Experiment | 1D |
| Number of Scans | 10000 |
| Receiver Gain | 10.0 |
| Relaxation Delay | 2.0000 |
| Pulse Width | 8.0000 |
| Acquisition Time | 1.3763 |
| Acquisition Date | 2025-05-04T09:37:25 |
| Modification Date | 2025-05-04T09:37:18 |
| Spectrometer Frequency | 100.64 |
| Spectral Width | 23809.5 |
| Lowest Frequency | -1702.5 |
| Nucleus | 13C |
| Acquired Size | 32768 |
| Spectral Size | 65536 |

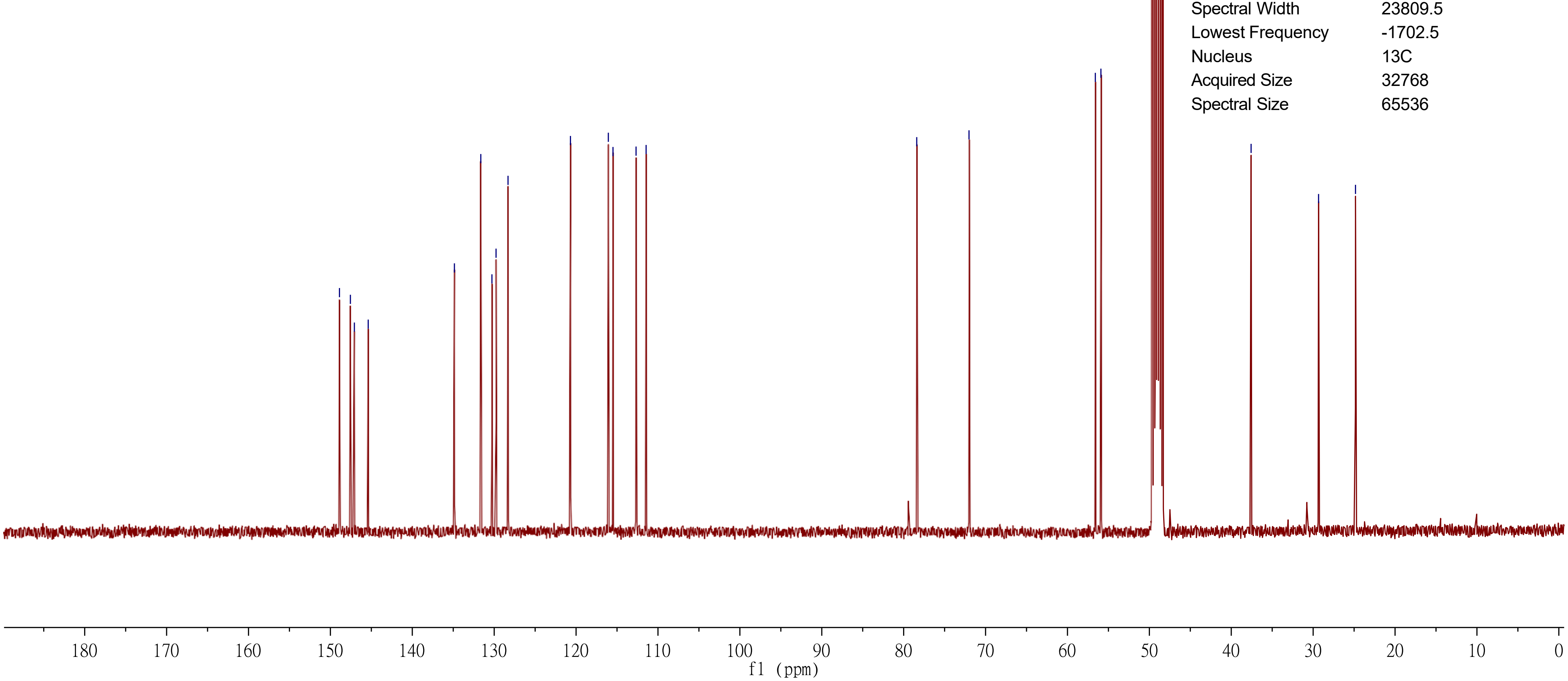

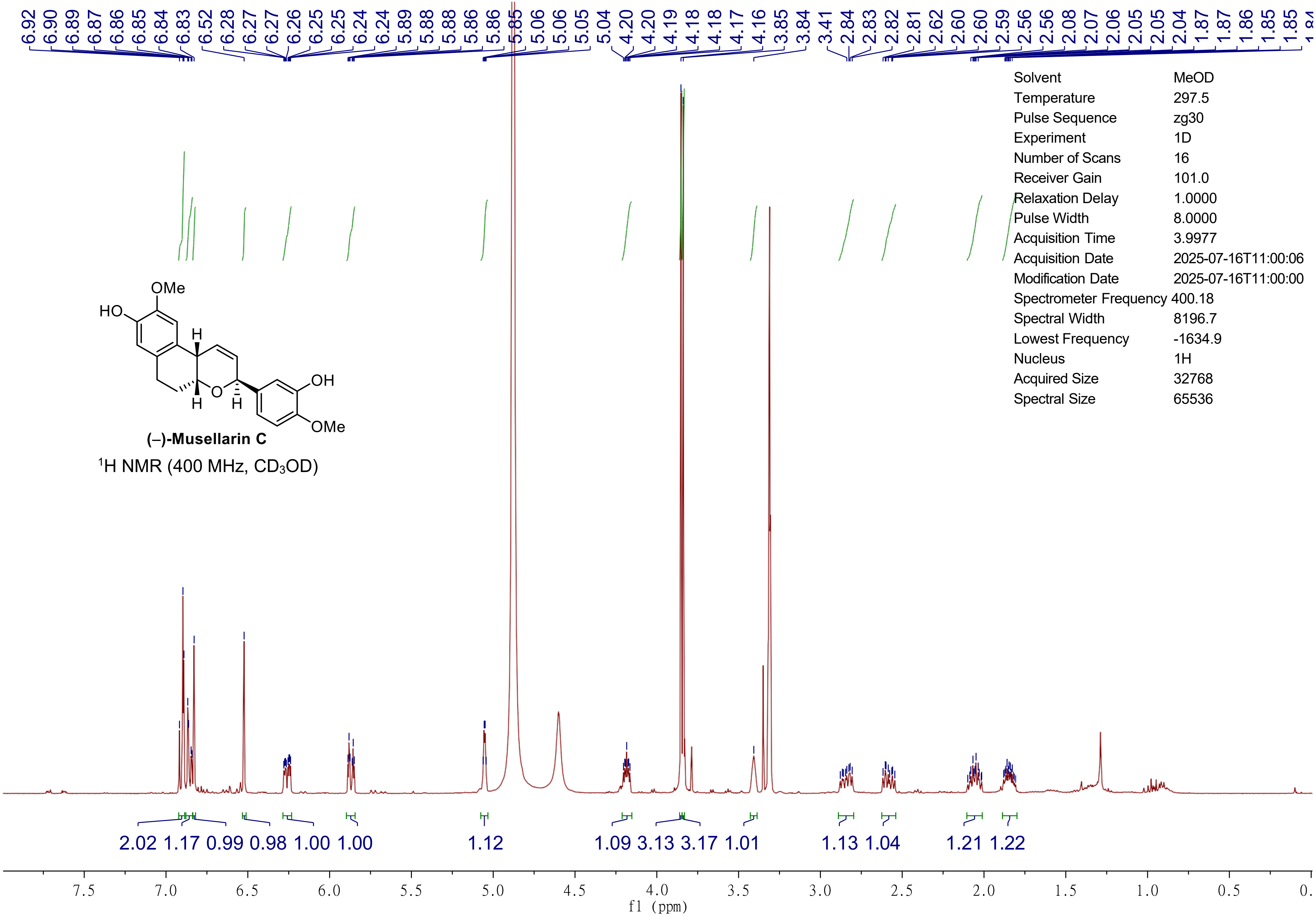

OMe
HO
H
OH
O
H
H
OMe
(–)-Musellarin C
1H NMR (400 MHz, CD3OD)
Solvent MeOD
Temperature 297.5
Pulse Sequence zg30
Experiment 1D
Number of Scans 16
Receiver Gain 101.0
Relaxation Delay 1.0000
Pulse Width 8.0000
Acquisition Time 3.9977
Acquisition Date 2025-07-16T11:00:06
Modification Date 2025-07-16T11:00:00
Spectrometer Frequency 400.18
Spectral Width 8196.7
Lowest Frequency -1634.9
Nucleus 1H
Acquired Size 32768
Spectral Size 65536
6.92 6.90 6.89 6.87 6.86 6.85 6.84 6.83 6.52 6.28 6.27 6.27 6.26 6.25 6.25 6.24 6.24 5.89 5.88 5.88 5.86 5.86 5.85 5.06 5.06 5.05 5.04 4.20 4.20 4.19 4.18 4.18 4.17 4.16 3.85 3.84 3.41 2.84 2.83 2.82 2.81 2.62 2.60 2.60 2.59 2.56 2.56 2.08 2.07 2.06 2.05 2.05 2.04 1.87 1.87 1.86 1.85 1.85
2.02 1.17 0.99 0.98 1.00 1.00 1.12 1.09 3.13 3.17 1.01 1.13 1.04 1.21 1.22
7.5 7.0 6.5 6.0 5.5 5.0 4.5 4.0 3.5 3.0 2.5 2.0 1.5 1.0 0.5
f1 (ppm)

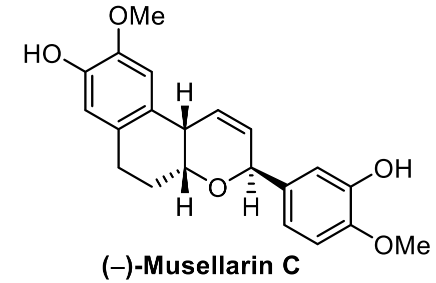


**(–)-Musellarin C**

$^{13}C\{^{1}H\}$ NMR (100 MHz, $CD_3OD$)

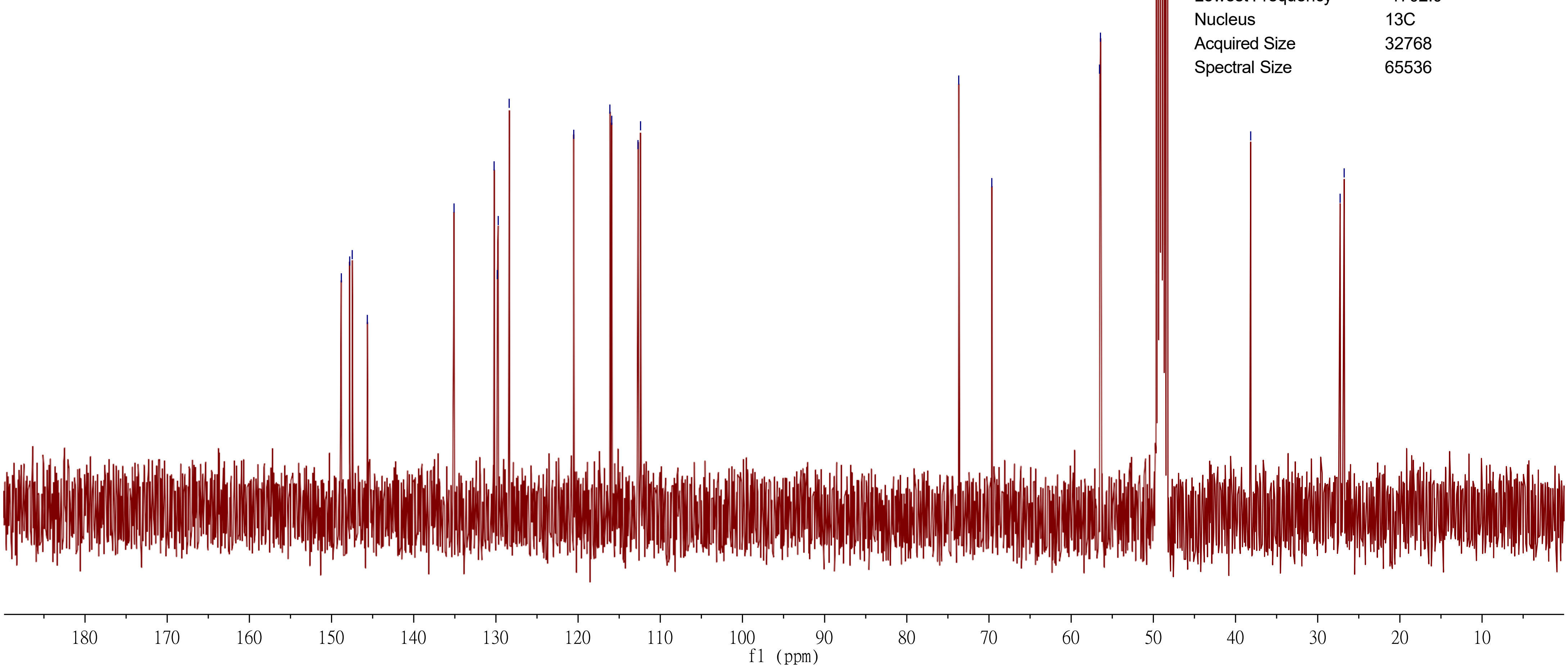


| | |
|---|---|
| Solvent | MeOD |
| Temperature | 298.5 |
| Pulse Sequence | zgpg30 |
| Experiment | 1D |
| Number of Scans | 705 |
| Receiver Gain | 10.0 |
| Relaxation Delay | 2.0000 |
| Pulse Width | 8.0000 |
| Acquisition Time | 1.3763 |
| Acquisition Date | 2025-07-16T11:41:59 |
| Modification Date | 2025-07-16T11:41:52 |
| Spectrometer Frequency | 100.64 |
| Spectral Width | 23809.5 |
| Lowest Frequency | -1702.0 |
| Nucleus | 13C |
| Acquired Size | 32768 |
| Spectral Size | 65536 |

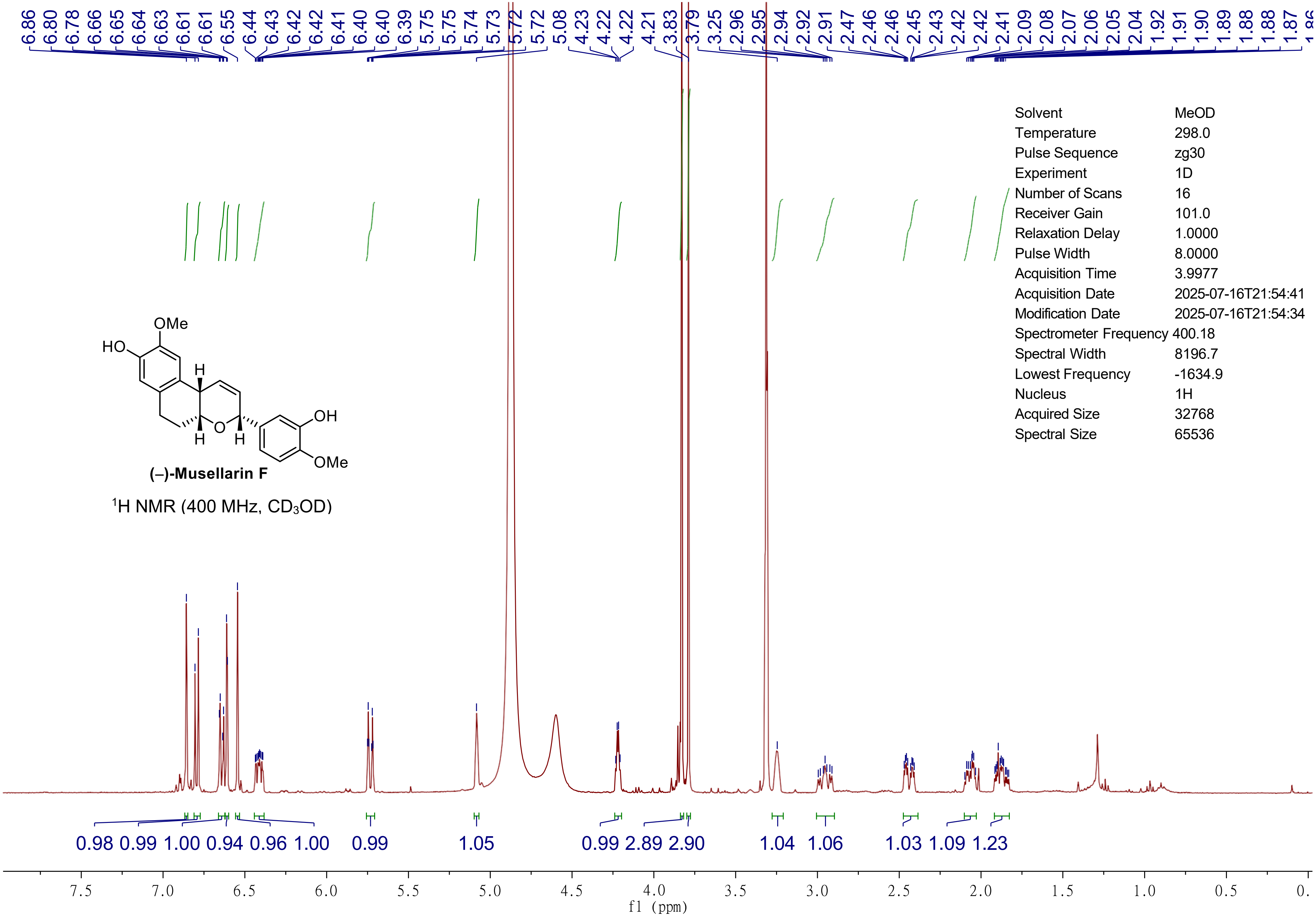
6.86 6.80 6.78 6.66 6.65 6.64 6.63 6.61 6.61 6.55 6.44 6.43 6.42 6.42 6.41 6.40 6.40 6.39 5.75 5.75 5.74 5.73 5.72 5.72 5.08 4.23 4.22 4.22 4.21 3.83 3.79 3.25 2.96 2.95 2.94 2.92 2.91 2.47 2.46 2.46 2.45 2.43 2.42 2.42 2.41 2.09 2.08 2.07 2.06 2.05 2.04 1.92 1.91 1.90 1.89 1.88 1.88 1.87
Solvent MeOD
Temperature 298.0
Pulse Sequence zg30
Experiment 1D
Number of Scans 16
Receiver Gain 101.0
Relaxation Delay 1.0000
Pulse Width 8.0000
Acquisition Time 3.9977
Acquisition Date 2025-07-16T21:54:41
Modification Date 2025-07-16T21:54:34
Spectrometer Frequency 400.18
Spectral Width 8196.7
Lowest Frequency -1634.9
Nucleus 1H
Acquired Size 32768
Spectral Size 65536
OMe
HO
H
OH
O
H
H
OMe
(–)-Musellarin F
$^{1}$H NMR (400 MHz, $CD_3OD$)
0.98 0.99 1.00 0.94 0.96 1.00 0.99 1.05 0.99 2.89 2.90 1.04 1.06 1.03 1.09 1.23
7.5 7.0 6.5 6.0 5.5 5.0 4.5 4.0 3.5 3.0 2.5 2.0 1.5 1.0 0.5 0.
f1 (ppm)

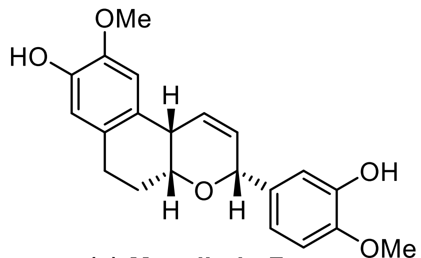


**(–)-Musellarin F**

$^{13}C\{^{1}H\}$ NMR (100 MHz, $CD_3OD$)

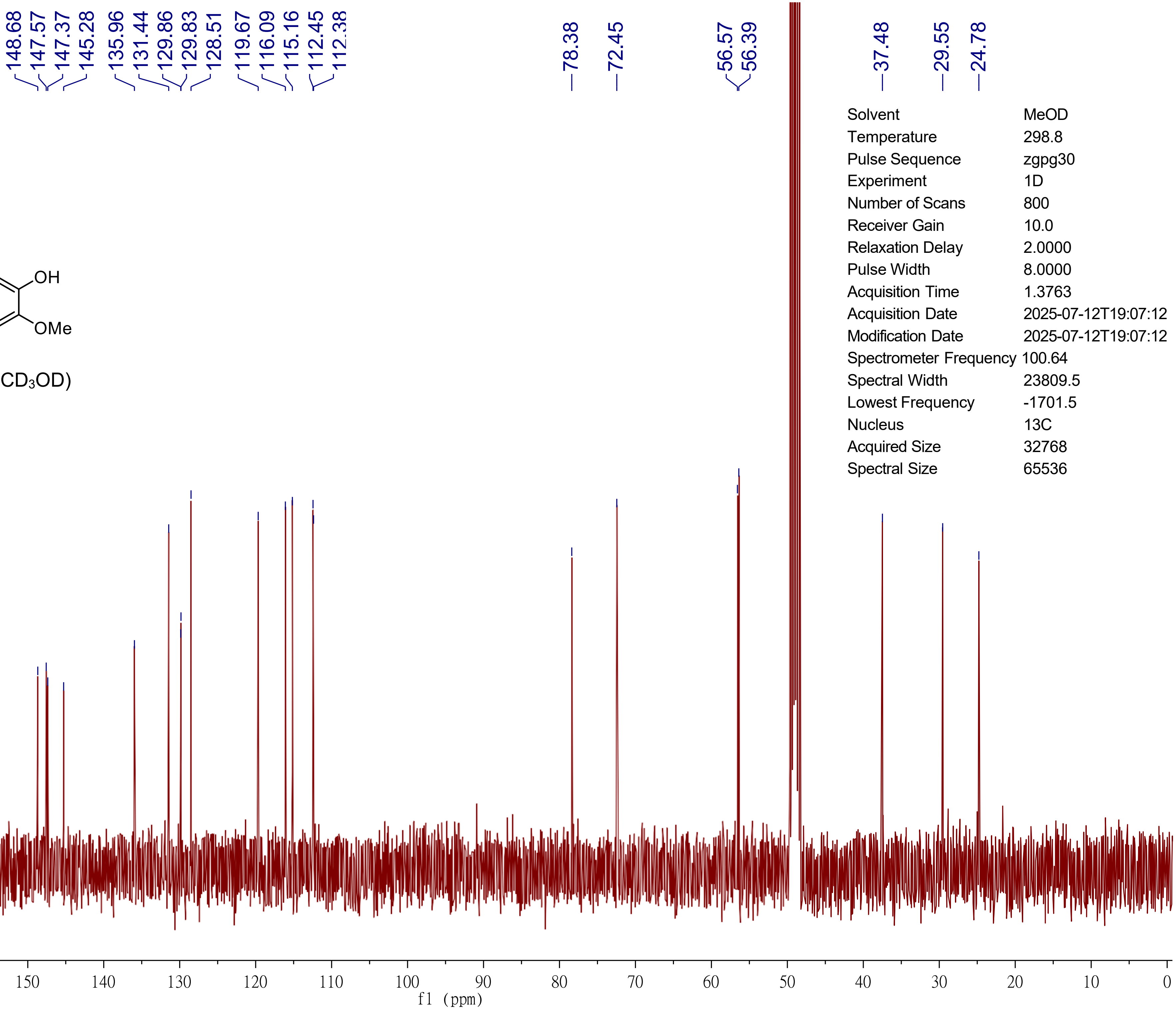

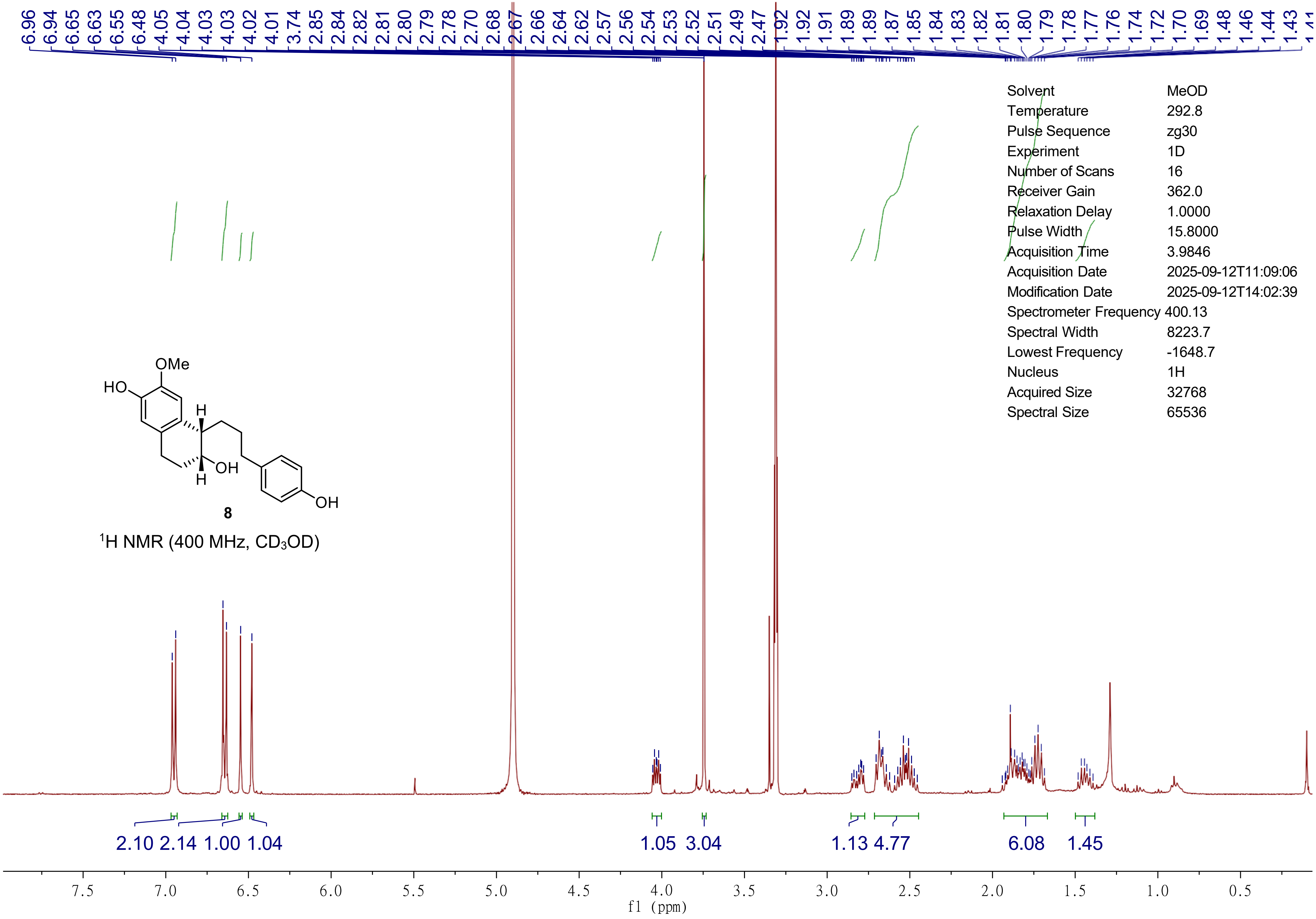


| | |
|---|---|
| Solvent | MeOD |
| Temperature | 292.8 |
| Pulse Sequence | zg30 |
| Experiment | 1D |
| Number of Scans | 16 |
| Receiver Gain | 362.0 |
| Relaxation Delay | 1.0000 |
| Pulse Width | 15.8000 |
| Acquisition Time | 3.9846 |
| Acquisition Date | 2025-09-12T11:09:06 |
| Modification Date | 2025-09-12T14:02:39 |
| Spectrometer Frequency | 400.13 |
| Spectral Width | 8223.7 |
| Lowest Frequency | -1648.7 |
| Nucleus | 1H |
| Acquired Size | 32768 |
| Spectral Size | 65536 |



**8**

$^1$H NMR (400 MHz, $CD_3OD$)

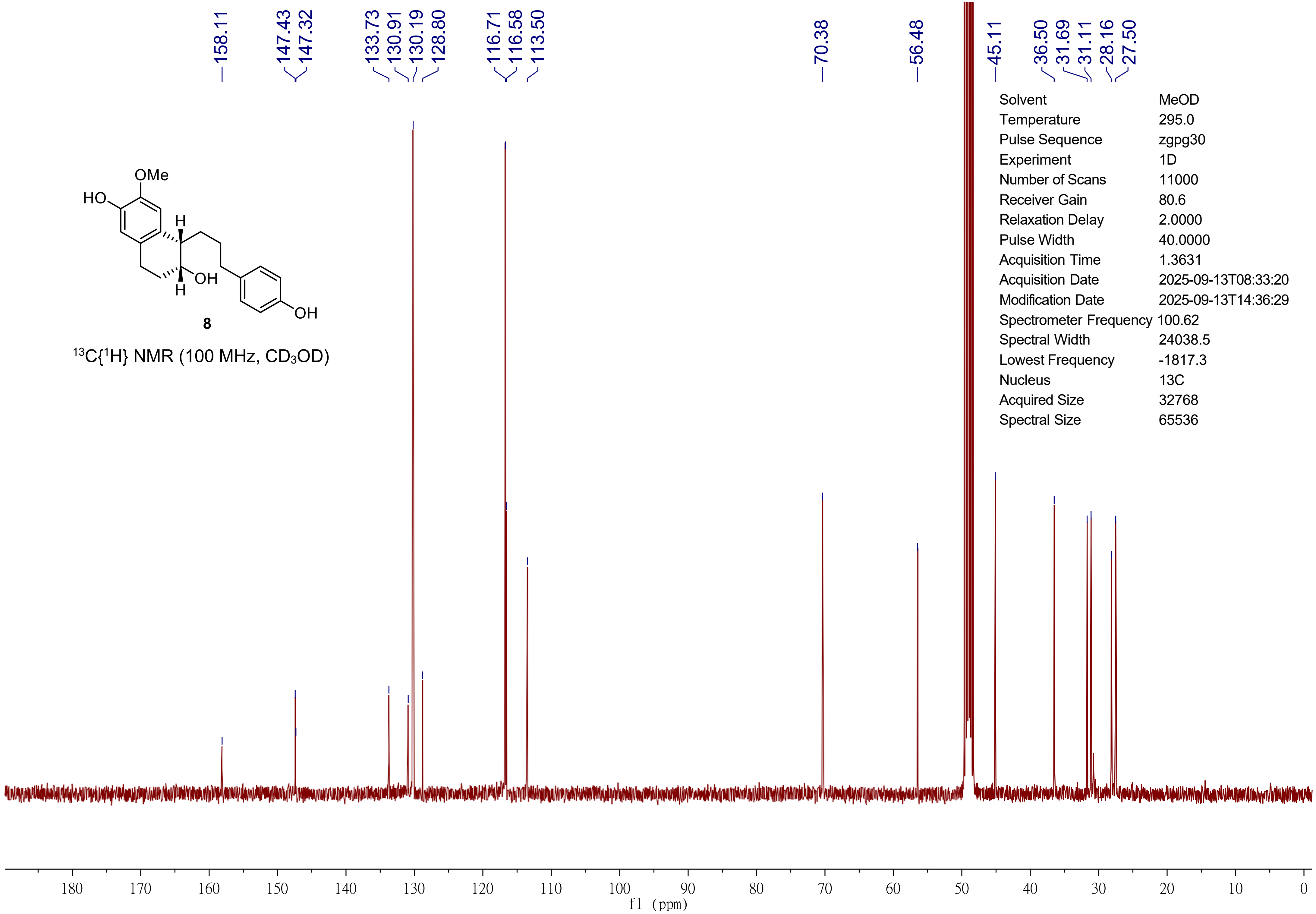

158.11
147.43
147.32
133.73
130.91
130.19
128.80
116.71
116.58
113.50
70.38
56.48
45.11
36.50
31.69
31.11
28.16
27.50
Solvent MeOD
Temperature 295.0
Pulse Sequence zgpg30
Experiment 1D
Number of Scans 11000
Receiver Gain 80.6
Relaxation Delay 2.0000
Pulse Width 40.0000
Acquisition Time 1.3631
Acquisition Date 2025-09-13T08:33:20
Modification Date 2025-09-13T14:36:29
Spectrometer Frequency 100.62
Spectral Width 24038.5
Lowest Frequency -1817.3
Nucleus 13C
Acquired Size 32768
Spectral Size 65536
OMe
HO
H
OH
H
OH
8
$^{13}C\{^{1}H\}$ NMR (100 MHz, $CD_3OD$)
180 170 160 150 140 130 120 110 100 90 80 70 60 50 40 30 20 10 0
f1 (ppm)

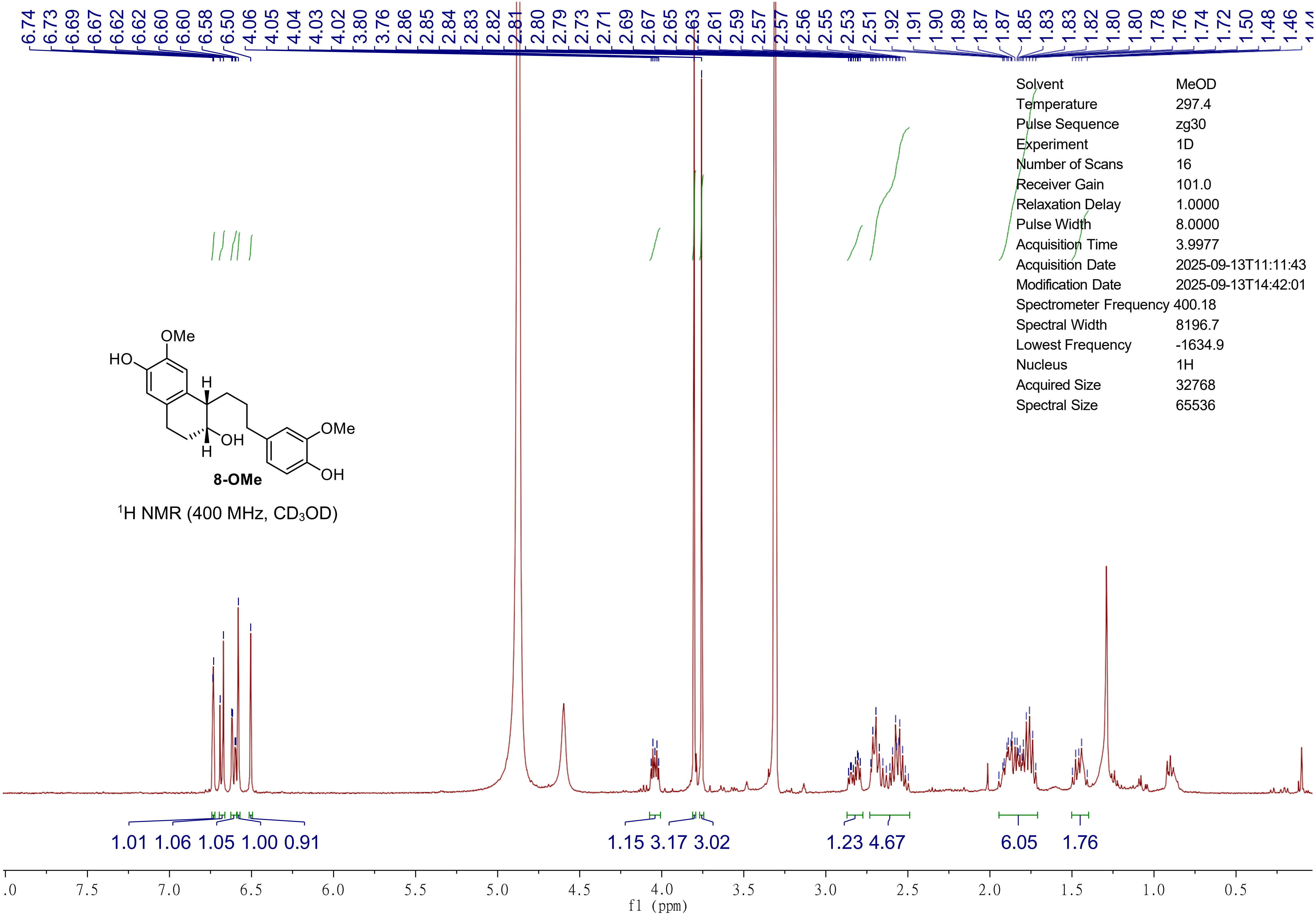
6.74 6.73 6.69 6.67 6.62 6.62 6.60 6.60 6.58 6.50 4.06 4.05 4.04 4.03 4.02 3.80 3.76 2.86 2.85 2.84 2.83 2.82 2.81 2.80 2.79 2.73 2.71 2.69 2.67 2.65 2.63 2.61 2.59 2.57 2.57 2.56 2.55 2.53 2.51 1.92 1.91 1.90 1.89 1.87 1.87 1.85 1.83 1.83 1.82 1.80 1.80 1.78 1.76 1.74 1.72 1.50 1.48 1.46
Solvent MeOD
Temperature 297.4
Pulse Sequence zg30
Experiment 1D
Number of Scans 16
Receiver Gain 101.0
Relaxation Delay 1.0000
Pulse Width 8.0000
Acquisition Time 3.9977
Acquisition Date 2025-09-13T11:11:43
Modification Date 2025-09-13T14:42:01
Spectrometer Frequency 400.18
Spectral Width 8196.7
Lowest Frequency -1634.9
Nucleus 1H
Acquired Size 32768
Spectral Size 65536
OMe
HO
H
OMe
OH
H
OH
8-OMe
1H NMR (400 MHz, CD3OD)
1.01 1.06 1.05 1.00 0.91
1.15 3.17 3.02
1.23 4.67
6.05
1.76
7.5 7.0 6.5 6.0 5.5 5.0 4.5 4.0 3.5 3.0 2.5 2.0 1.5 1.0 0.5
f1 (ppm)

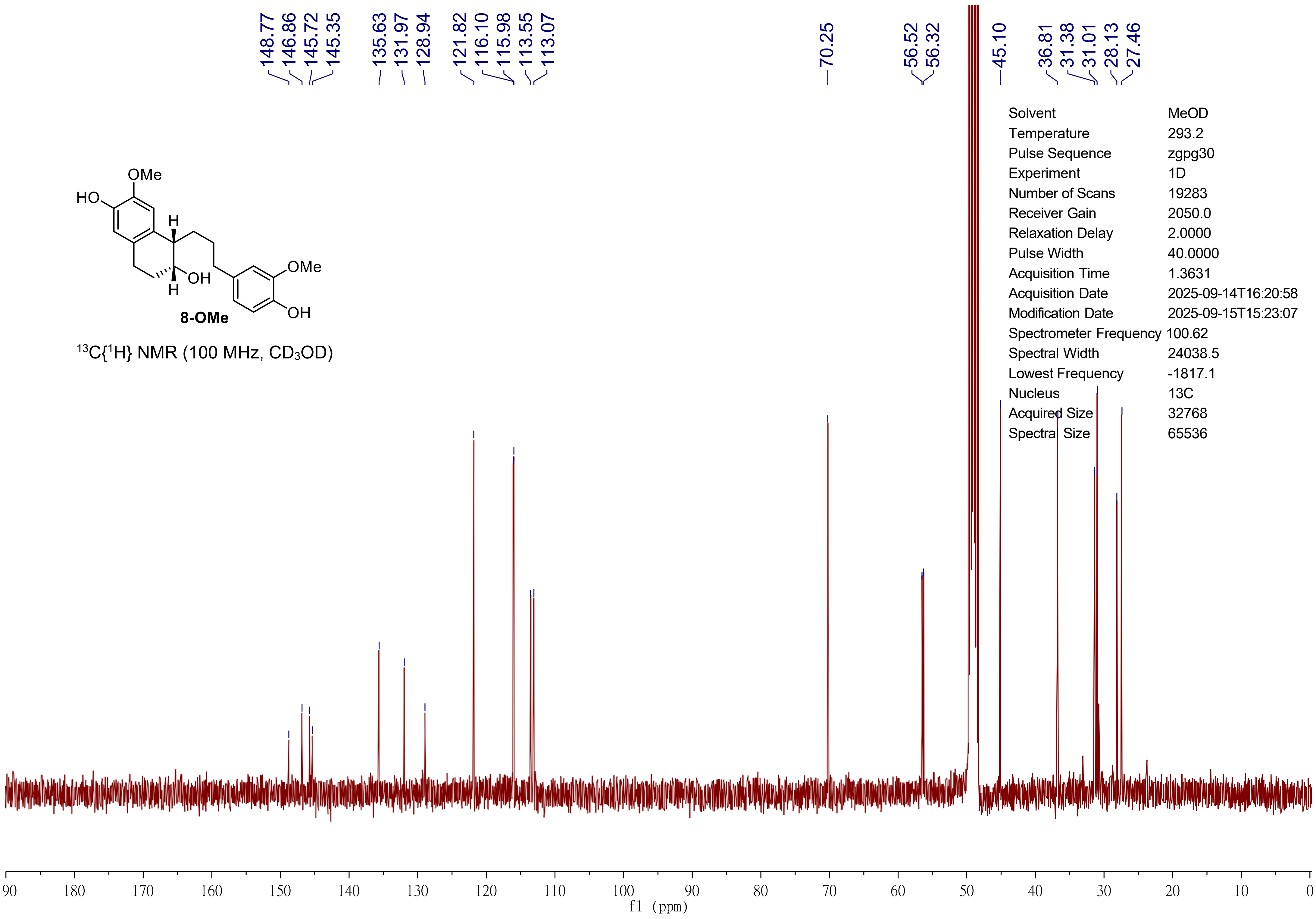

148.77
146.86
145.72
145.35
135.63
131.97
128.94
121.82
116.10
115.98
113.55
113.07
70.25
56.52
56.32
45.10
36.81
31.38
31.01
28.13
27.46
OMe
HO
H
OMe
OH
H
OH
8-OMe
13C{1H} NMR (100 MHz, CD3OD)
Solvent MeOD
Temperature 293.2
Pulse Sequence zgpg30
Experiment 1D
Number of Scans 19283
Receiver Gain 2050.0
Relaxation Delay 2.0000
Pulse Width 40.0000
Acquisition Time 1.3631
Acquisition Date 2025-09-14T16:20:58
Modification Date 2025-09-15T15:23:07
Spectrometer Frequency 100.62
Spectral Width 24038.5
Lowest Frequency -1817.1
Nucleus 13C
Acquired Size 32768
Spectral Size 65536
90 180 170 160 150 140 130 120 110 100 90 80 70 60 50 40 30 20 10 0
f1 (ppm)

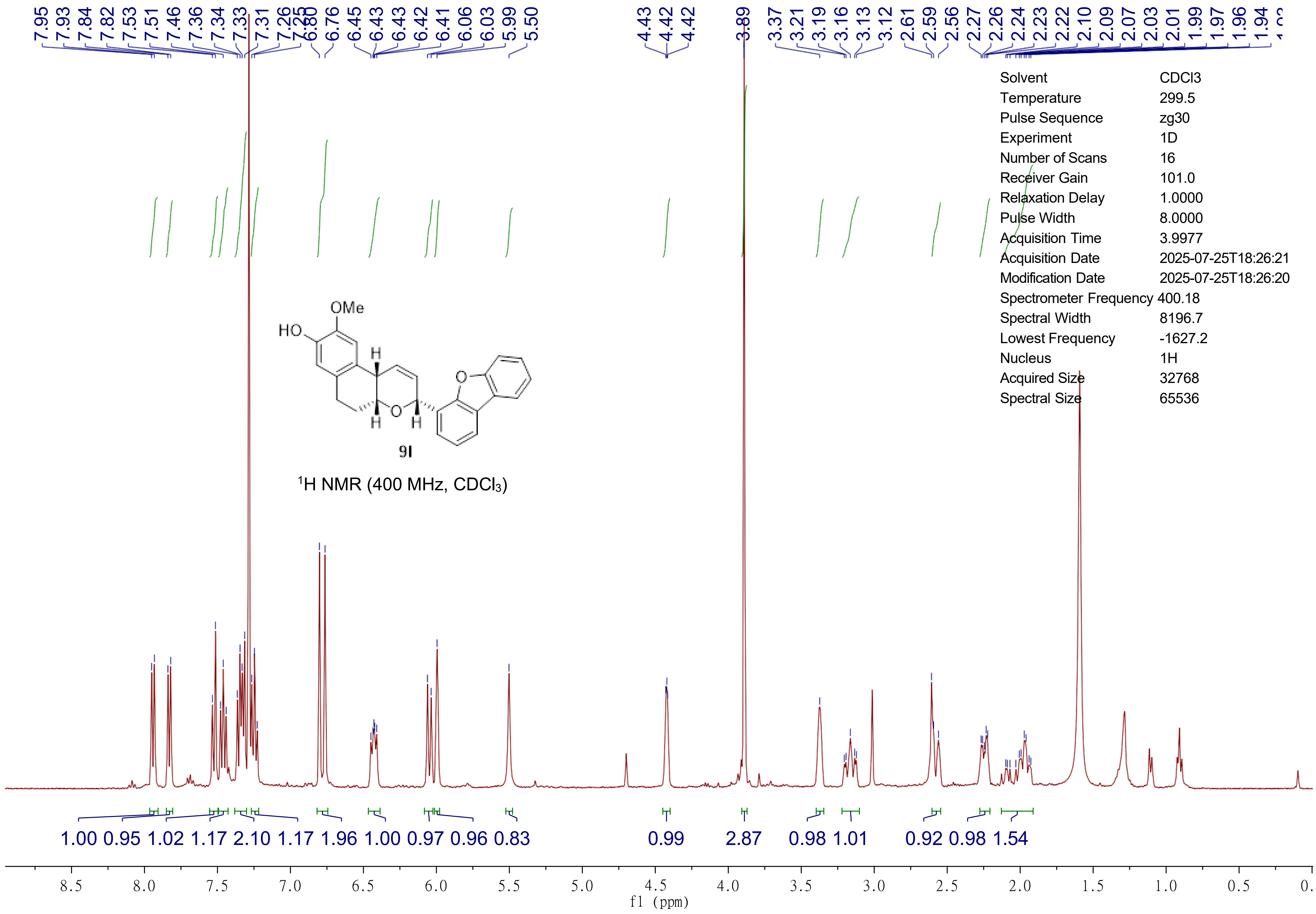
7.95 7.93 7.84 7.82 7.53 7.51 7.46 7.36 7.34 7.33 7.31 7.26 7.25 6.80 6.76 6.45 6.43 6.43 6.42 6.41 6.06 6.03 5.99 5.50 4.43 4.42 4.42 3.89 3.37 3.21 3.19 3.16 3.13 3.12 2.61 2.59 2.56 2.27 2.26 2.24 2.23 2.22 2.10 2.09 2.07 2.03 2.01 1.99 1.97 1.96 1.94
Solvent CDCl3
Temperature 299.5
Pulse Sequence zg30
Experiment 1D
Number of Scans 16
Receiver Gain 101.0
Relaxation Delay 1.0000
Pulse Width 8.0000
Acquisition Time 3.9977
Acquisition Date 2025-07-25T18:26:21
Modification Date 2025-07-25T18:26:20
Spectrometer Frequency 400.18
Spectral Width 8196.7
Lowest Frequency -1627.2
Nucleus 1H
Acquired Size 32768
Spectral Size 65536
OMe
HO
H
O
H
H
O
9l
1H NMR (400 MHz, CDCl3)
1.00 0.95 1.02 1.17 2.10 1.17 1.96 1.00 0.97 0.96 0.83 0.99 2.87 0.98 1.01 0.92 0.98 1.54
8.5 8.0 7.5 7.0 6.5 6.0 5.5 5.0 4.5 4.0 3.5 3.0 2.5 2.0 1.5 1.0 0.5 0.
f1 (ppm)

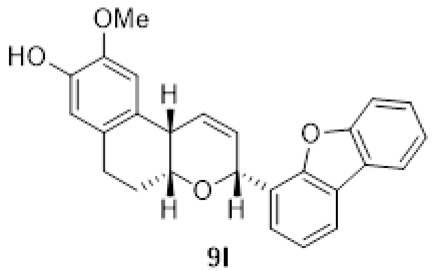


**9l**

$^{13}C\{^{1}H\}$ NMR (100 MHz, $CDCl_3$)

| | |
|---|---|
| Solvent | CDCl3 |
| Temperature | 302.5 |
| Pulse Sequence | zgpg30 |
| Experiment | 1D |
| Number of Scans | 709 |
| Receiver Gain | 10.0 |
| Relaxation Delay | 2.0000 |
| Pulse Width | 8.0000 |
| Acquisition Time | 1.3763 |
| Acquisition Date | 2025-07-25T19:09:04 |
| Modification Date | 2025-07-25T19:09:04 |
| Spectrometer Frequency | 100.64 |
| Spectral Width | 23809.5 |
| Lowest Frequency | -1827.7 |
| Nucleus | 13C |
| Acquired Size | 32768 |
| Spectral Size | 65536 |

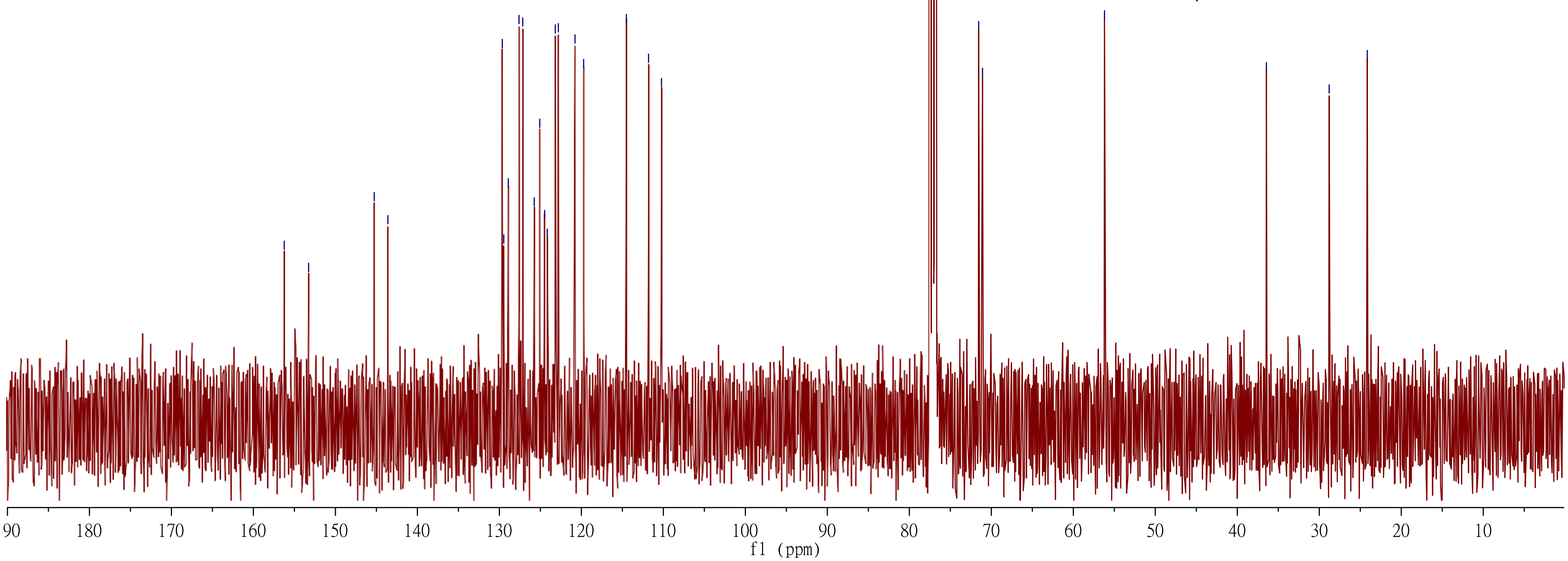

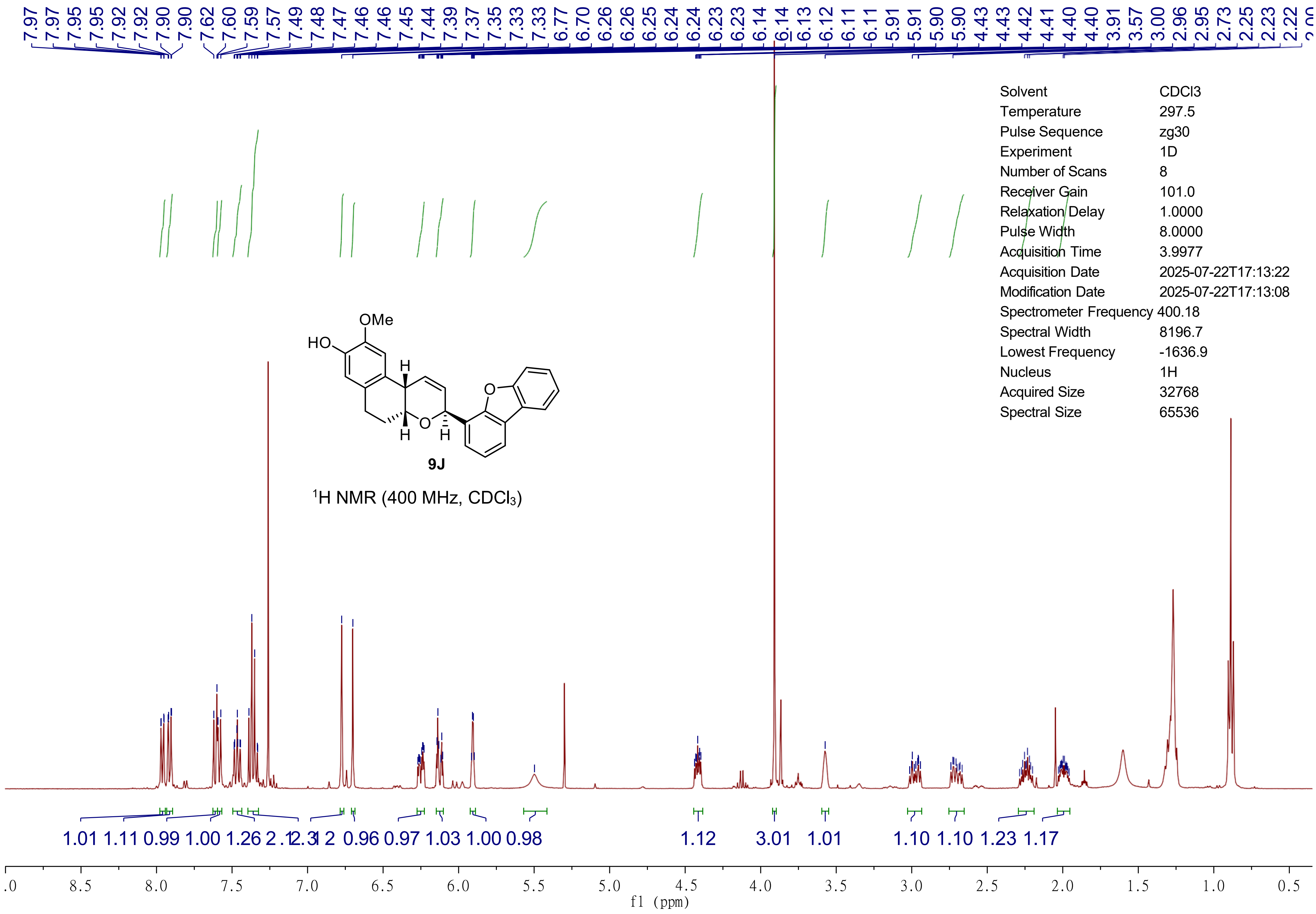
7.97 7.97 7.95 7.95 7.92 7.92 7.90 7.90 7.62 7.60 7.59 7.57 7.49 7.48 7.47 7.46 7.46 7.45 7.44 7.39 7.37 7.35 7.33 7.33 6.77 6.70 6.26 6.26 6.25 6.24 6.24 6.23 6.23 6.14 6.14 6.13 6.12 6.11 6.11 5.91 5.91 5.90 5.90 4.43 4.43 4.42 4.41 4.40 4.40 3.91 3.57 3.00 2.96 2.95 2.73 2.25 2.23 2.22
Solvent CDCl3
Temperature 297.5
Pulse Sequence zg30
Experiment 1D
Number of Scans 8
Receiver Gain 101.0
Relaxation Delay 1.0000
Pulse Width 8.0000
Acquisition Time 3.9977
Acquisition Date 2025-07-22T17:13:22
Modification Date 2025-07-22T17:13:08
Spectrometer Frequency 400.18
Spectral Width 8196.7
Lowest Frequency -1636.9
Nucleus 1H
Acquired Size 32768
Spectral Size 65536
OMe
HO
H
O
H
H
O
9J
$^{1}$H NMR (400 MHz, $CDCl_3$)
1.01 1.11 0.99 1.00 1.26
0.96 0.97 1.03 1.00 0.98
1.12
3.01 1.01
1.10 1.10 1.23 1.17
8.5 8.0 7.5 7.0 6.5 6.0 5.5 5.0 4.5 4.0 3.5 3.0 2.5 2.0 1.5 1.0 0.5
f1 (ppm)

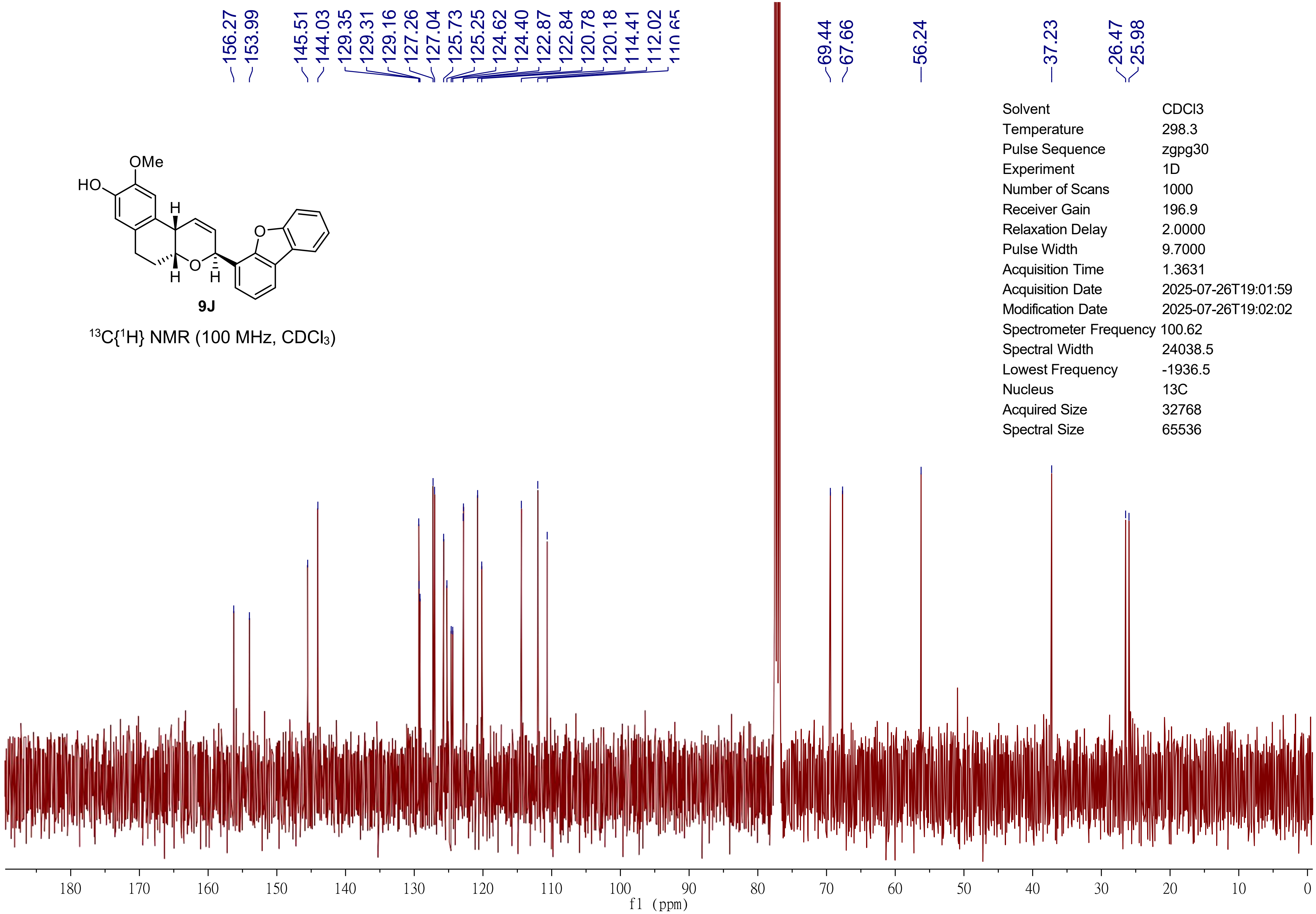


**9J**

$^{13}C\{^{1}H\}$ NMR (100 MHz, $CDCl_3$)
